# Can explainable AI classify shrike (Laniidae) eggs by uncovering species-wide pigmentation patterns?

**DOI:** 10.1371/journal.pone.0321532

**Published:** 2025-05-02

**Authors:** Paweł Pstrokoński, Łukasz Roszkowiak, Anna Korzyńska, Wojciech Wójcik, Martin Päckert, Joanna Rosenberger, Dominika Mierzwa-Szymkowiak, Magdalena Sepkowska, Jan Lontkowski, Marek Słupek, Krzysztof Damaziak

**Affiliations:** 1 Department of Animal Breeding, Institute of Animal Sciences, Warsaw University of Life Sciences, Warsaw, Poland; 2 Laboratory of Processing and Analysis of Microscopic Images, Nalecz Institute of Biocybernetics and Biomedical Engineering PAS, Warsaw, Poland; 3 Senckenberg Natural History Collections Dresden, Dresden, Germany; 4 Division of Poultry Breeding, Faculty of Biology, Institute of Animal Breeding, Wroclaw University of Environmental and Life Sciences, Wroclaw, Poland; 5 Museum and Institute of Zoology, Polish Academy of Sciences, Warsaw, Poland; 6 Count Antoni Ostrowski Museum in Tomaszow Mazowiecki, Tomaszow Mazowiecki, Poland; 7 Museum of Natural History, Wroclaw University, Wroclaw, Poland; 8 Museum of Jacek Malczewski in Radom, Radom, Poland; The University of Tokyo: Tokyo Daigaku, JAPAN

## Abstract

The complex patterns on bird eggs, characterized by their replicability, distinctiveness, and intricacy, play significant roles in avian biology, including camouflage, protection from brood parasites, protecting embryos, nest identification, strengthening eggshells, and female sexual selection. The genus *Lanius*, known for its distinctive pigmentation patterns, shows considerable variability within species, making it an intriguing but poorly understood group. We applied Explainable AI (XAI) methods to uncover pigmentation patterns that represent species-wide identification signatures. To do this, we used Convolutional Neural Networks (CNNs) to classify shrike eggs and explore potential correlations between egg identification and eggshell patterns. Our CNN model achieved over 95% accuracy in predicting species, but identifying specific discriminative features proved difficult, as the model only highlighted general trends. This method could help organize collections and verify species affiliation in global ornithological collections, which often face challenges such as missing or illegible labels. CNNs can enhance species identification and improve the accuracy of ornithological studies. Despite some challenges, the potential applications of this research in avian biology and museum collections are promising. It offers new insights into the role of eggshell patterns in avian evolutionary strategies. This approach not only enriches our understanding of egg pigmentation but also contributes to advancements in studies spanning from ecology to biomedical research.

## Introduction

Like humans, color is crucial for birds because they rely on visual stimuli. However, birds can see beyond the human visual spectrum, perceiving ultraviolet light and experiencing a richer array of colors. It is not only feather color that matters; the complex patterns on bird eggs also play a vital role in their biology. These patterns serve several key functions, such as camouflaging the eggs from predators [1–3], preventing brood parasitism [4–7], and protecting the embryo from solar radiation, water loss, and infections [8,9]. They also help birds locate their nests in colonies [10,11], strengthen the eggshell structure [12], and contribute to play a role in sexual selection by indicating female health and signalling genetic quality [13–15]. According to Stoddard et al. [6], the theory of egg pattern signatures focuses on strategies shaped by host-parasite interactions. The theory should feature three characteristics: (I) replicability, meaning low variation within a clutch, (II) distinctiveness, meaning high variation between clutches, and (III) complexity, meaning the signature is difficult to reproduce.

Intraspecies variability in eggshell color and pigmentation can have various causes, making shrikes an interesting but poorly known group of birds. The family includes two genera: *Lanius*, with 32 species, and *Eurocephalus*, with 2 species [16,17]. All shrikes lay eggs with distinctive pigmentation, with pigment spots arranged in a ring around the blunt end of the egg. Despite a common pattern within species, pigmentation variation is among the highest in the dozens of bird species studied by Miksik et al. [18]. Another notable observation is that shrikes were once common hosts for cuckoos in Europe, but this parasitism began to decline in the 1960s [19,20]. Although several attempts have been made to determine the reasons for the cuckoo’s withdrawal as a shrike parasite, the potential role of changes in eggshell color has not been explored, despite being observed in other bird species [6,21,22]. In addition, shrikes tend to nest close to one another, but only within a single species. For example, the population of red-backed shrikes (*Lanius collurio*) is increasing in Central Europe, while the ranges of lesser gray shrikes (*Lanius minor*) and woodchat shrikes (*Lanius senator*) are shrinking [23,24]. This may be related not only to food availability and varying tolerance to thermal conditions but also to breeding competition, including brood parasitism between species.

Based on the findings of Stoddard et al. [6] regarding the presence of individual maternal signatures on the eggshell surface, we hypothesize that this phenomenon may have served as a defensive tool to eliminate shrikes as hosts for the cuckoo and to defend against interspecies and intraspecies parasitism within the genus *Lanius* [25]. This may have contributed to the current distribution of shrike species in Europe. Studying eggshell pigmentation presents challenges, mainly due to the lack of tools to accurately quantify the information hidden in a female’s signature, which birds interpret in ways that are still unknown.

Despite existing challenges, researchers have recently focused on analyzing bird egg spottiness using various approaches and appliances. Surmacki et al. [26] found high repeatability in red-backed shrike clutches, suggesting that these traits may be shaped by natural selection. Using principal component analysis, they identified three key egg features: curvature, pigmentation intensity, and contrast. Our study builds on this by using Explainable AI (XAI) techniques to explore whether these consistent pigmentation features can be systematically used to identify species accurately across different *Lanius* species.

Stoddard et al. [6] developed a new tool, NaturePatternMatch, to check if common cuckoo (*Cuculus canorus*) hosts have evolved distinct, recognizable signatures on their eggs. The NaturePatternMatch tool uses the SIFT algorithm, a computer vision technique that detects, describes, and matches local features within images. However, the algorithm has a limitation because it requires manual parameter configuration. Although Stoddard et al. [6] invested significant effort into manual parameterization, it does not ensure generalizability. In contrast, our approach automates this process as much as possible.

Later, Gómez and Liñán-Cembrano [27] presented SpotEgg, a computing infrastructure for analyzing eggshell coloration and spottiness. SpotEgg extracts a wide range of features, as defined by its creators, encompassing geometrical properties, spottiness metrics, fractal dimension, and coloration attributes. It calculates these features for both whole regions of interest and individual spots. While this approach shows promise for gaining valuable insights, its reliance on Matlab licensing and the labor-intensive process of feature examination limit its utility.

Since then, both tools have been used in various studies [28,29]. Nevertheless, neither method allows for the explicit identification of species-specific discriminative features on the eggshell surface, as both rely on relative comparisons of image pairs. This limitation is further compounded by the lack of advanced tools to quantify eggshell signature information, leaving a gap in our understanding of how birds perceive these visual signals.

In this study, we aimed to apply XAI methods to uncover pigmentation patterns that serve as species-wide identification signatures. We proposed using Convolutional Neural Networks (CNNs) to identify shrike eggs, exploring their potential for rapid and efficient recognition. Furthermore, we applied XAI techniques to investigate correlations between shrike egg identification and the patterns found on their eggshells. We chose CNNs to analyze eggshell pigmentation variability because researchers have found this technology essential in various scientific fields, including disease lesion detection [30], image reconstruction [31–33], gene expression analysis [34,35], and satellite imagery processing [36,37].

To sum up, this study offers the following contributions: (I) We provide a comprehensive dataset of digital images of shrike eggs sourced from diverse locations; (II) We implement a machine learning approach to classify egg images using various image sizes; (III) We demonstrate explainability through Shapley Additive Explanations (SHAP) and Gradient-weighted Class Activation Mapping (Grad-CAM) methods to provide insight into the decision-making process of the proposed machine learning model.

Our main goal with this comprehensive evaluation framework is to uncover the complex interactions between species classification, pigmentation intensity, and egg shape. We aim to reveal patterns and relationships that go beyond simple visual inspection. This approach not only enriches our understanding of egg pigmentation but also contributes to advancements in studies spanning from ecology to biomedical research, where similar pattern recognition techniques are applied to analyze complex biological structures.

## Results

First, we compared the proposed augmentation strategies while training CNN models for the four-class classification task. The results in Table 1 show that medium augmentation provides only a slight improvement in numerical results based on the evaluation performed on the test set. We used 3-fold cross-validation and obtained consistent results, as shown by the mean and standard deviation values in Table 1.

**Table 1:** Comparison of tested augmentation schemes based on several classification performance metrics. The values represent the mean and standard deviation from a 3-fold cross-validation.

Augmentation	Flips	Rotation	Random crop	Brightness & contrast modification	Precision	Recall	Accuracy
None	No	No	No	No	0.92 (SD = 0.03)	0.91 (SD = 0.02)	0.92 (SD = 0.02)
Simple	Yes	Limited	No	No	0.94 (SD = 0.04)	0.92 (SD = 0.02)	0.94 (SD = 0.03)
Medium	Yes	Full	Yes	No	0.94 (SD = 0.03)	0.93 (SD = 0.03)	0.95 (SD = 0.03)
Heavy	Yes	Full	Yes	Yes	0.92 (SD = 0.04)	0.94 (SD = 0.03)	0.94 (SD = 0.05)

Second, we validated the idea of balancing the dataset by undersampling the majority class (red-backed shrike) and oversampling the minority classes using augmentation schemes. We compare the raw dataset, the dataset with only augmentation (oversampling), and the balanced dataset in Table 2. We applied a 5-fold cross-validation approach in this experiment. The balanced dataset produced the best results, proving the value of this approach.

**Table 2:** Impact of dataset balancing with only oversampling (w/aug) and the use of both undersampling and oversampling (balanced). The values represent the mean and standard deviation from a 5-fold cross-validation.

				F1-score			
	Precision	Recall	Accuracy	Red-backed shrike	Lesser gray shrike	Great gray shrike	Woodchat shrike
**VGG16**	0.926 (SD = 0.005)	0.920 (SD = 0.010)	0.934 (SD = 0.005)	0.956 (SD = 0.005)	0.894 (SD = 0.015)	0.966 (SD = 0.005)	0.874 (SD = 0.015)
**w/aug**	0.928 (SD = 0.015)	0.918 (SD = 0.018)	0.932 (SD = 0.008)	0.948 (SD = 0.008)	0.908 (SD = 0.029)	0.970 (SD = 0.007)	0.856 (SD = 0.025)
**Balanced**	0.957 (SD = 0.015)	0.957 (SD = 0.015)	0.957 (SD = 0.015)	0.950 (SD = 0.010)	0.960 (SD = 0.026)	0.983 (SD = 0.006)	0.927 (SD = 0.023)

Third, we analyzed the impact of rescaling the input images on the classification results. We tested image sizes of 256 × 256, 128 × 128, and 64 × 64 pixels and compared the results in Table 3, showing the best-performing model values.

**Table 3:** Classification metric results for each class with different input image sizes.

Shrike species	Input image size (pixels)								
	256 × 256			128 × 128			64 × 64		
	Accuracy	0.97		Accuracy	0.95		Accuracy	0.95	
	Precision	Recall	F1-score	Precision	Recall	F1-score	Precision	Recall	F1-score
Red-backed shrike	0.97	0.94	0.96	0.91	0.97	0.94	0.96	0.94	0.95
Lesser gray shrike	0.99	0.98	0.99	0.97	0.96	0.96	0.93	0.98	0.95
Great gray shrike	0.99	1	0.99	0.99	0.98	0.99	0.99	0.98	0.98
Woodchat shrike	0.93	0.96	0.95	0.94	0.89	0.91	0.93	0.92	0.92

**Fig 1:**
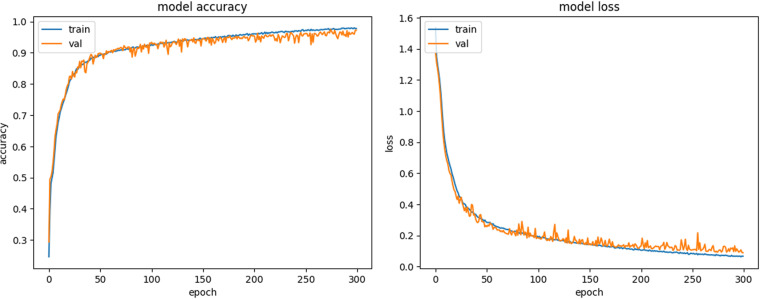
Example of VGG16 model training, showing model accuracy and loss for the train (blue line) and validation (orange line) subsets.

**Fig 2:**
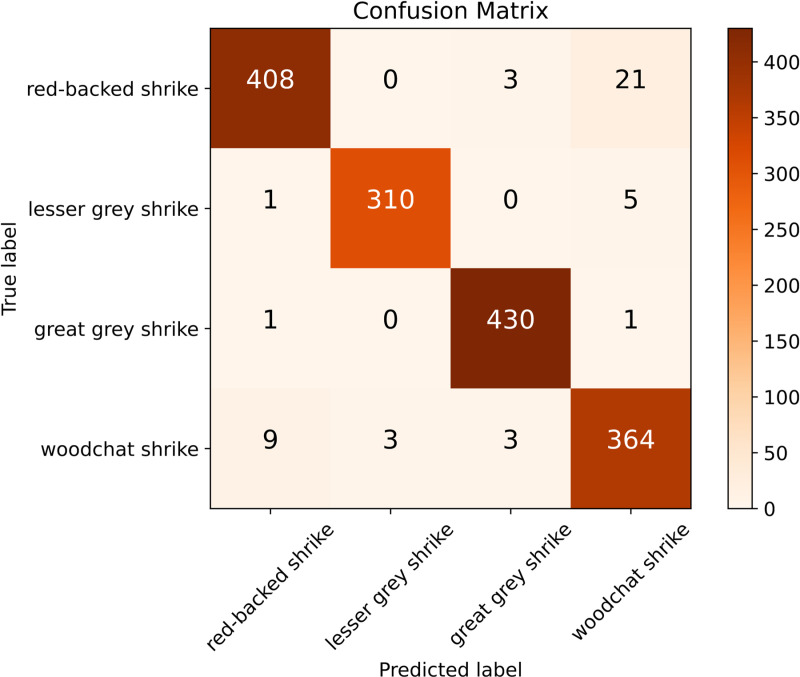
Confusion matrix for the test set classification, displaying the number of correctly and incorrectly predicted examples for each class. The most common errors involve misclassifying red-backed shrike as woodchat shrike, while the second most frequent mistake is predicting lesser gray shrike instead of woodchat shrike. Correct classifications appear along the diagonal, highlighting the model’s strong accuracy for each species.

We present the example of training scenario details in Figs 1 and 2.

### Qualitative results

In this study, we assess Grad-CAM results to evaluate model performance, since other approaches, such as quantitative metrics and localization accuracy, are not applicable or feasible. Quantitative metrics like IoU require ground truth object masks. Similarly, localization accuracy metrics rely on annotated object locations, which is not feasible in this study but could be generated using specialized tools [38]. Our experiment focuses on evaluating model performance by analyzing the correlation between Grad-CAM activations and model confidence scores for correct and incorrect predictions. This approach offers the most relevant and informative way to assess Grad-CAM’s effectiveness. We specifically analyze correct predictions to determine whether the regions highlighted by Grad-CAM align with the most discriminative features that influence the model’s predictions. This assessment provides a complementary perspective that may support the evaluation of SHAP values.

**Fig 3:**
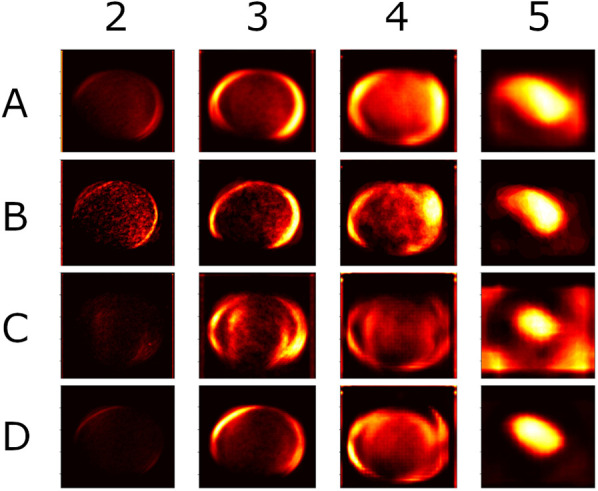
Aggregated Grad-CAM heatmaps across distinct classes representing various species. Each row corresponds to a specific species (A: red-backed shrike; B: lesser gray shrike; C: great gray shrike; D: woodchat shrike), while each column illustrates accumulations derived from different levels of the VGG16 network. From left to right, these levels include the 2nd convolution block, 3rd convolution block, 4th convolution block, and the final convolution block.

In addition to generating typical Grad-CAM heatmaps for individual instances, we created aggregated images across different classes to represent various species (Fig 3). Aggregating Grad-CAM results consolidates insights from multiple samples or classes, improving interpretability, robustness, and generalization. This approach reduces noise, minimizes bias, and helps identify overarching patterns.

### Quantitative results

**Fig 4:**
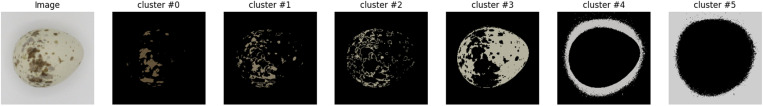
Example of color clustering employing the K-means clustering algorithm to identify regions with varying pigmentation intensities.

We primarily evaluate SHAP results by visualizing SHAP values overlaid onto input images (Fig 4). Typically, distinct regions representing each class become apparent based on positive values. Adjusting the scale for each example is crucial because the maximum value can vary significantly across instances. This variability across samples makes it challenging to generalize SHAP values across different datasets or populations. Moreover, intricate models often yield SHAP values that are harder to interpret due to their complexity, complicating quantitative evaluation. To address these challenges, we propose a method for quantitatively assessing SHAP results by focusing on the major impact of positive SHAP values. Our approach calculates the mean values of positive SHAP values within specific regions of interest, which in this study represent various pigmentation intensities. We segmented these regions from the images using a color segmentation technique with the K-means clustering algorithm. We used a set number of six clusters to calculate binary masks, selecting four clusters, as the last one represented the background.

We also incorporated automatically estimated egg boundary regions, which we then dilated for inclusion in the evaluation process. In addition to calculating mean values, we counted the number of pixels with values falling within the second (Q2) and third quartiles (Q3), which correspond to over 50% and over 75% of the maximum value, respectively (Table 4). Using these metrics, we aimed to explore the correlation between each species, the intensity of pigmentation, and the shape of the egg based on the border region.

**Table 4:** Major impact of SHAP positive values for each cluster and edge is based on the count of pixels with values falling within the second (Q2) and third (Q3) quartiles, corresponding to over 50% and 75% of the maximal value, respectively.

Quartile	Q2					Q3				
Clusters	Cluster_0	Cluster_1	Cluster_2	Cluster_3	Edges	Cluster_0	Cluster_1	Cluster_2	Cluster_3	Edges
Red-backed shrike	578	630	746	364	437	427	459	516	226	294
Lesser gray shrike	445	367	602	230	376	225	421	147	251	232
Great gray shrike	846	628	462	162	290	524	409	345	109	182
Woodchat shrike	514	407	334	113	214	307	209	129	45	110

## Discussion

In examining our deep learning model’s decision-making through XAI techniques, we found that integrating both Grad-CAM and SHAP DeepExplainer was crucial. Grad-CAM enabled us to visually identify the key image regions influencing predictions, improving our understanding of the model’s attention mechanisms. At the same time, SHAP DeepExplainer offered detailed insights into the contributions of individual pixels or regions. By using these XAI tools, we increased transparency and tackled the interpretability challenges of complex deep learning architectures.

### Data collection

Our dataset is notably larger than those in similar studies, containing 2236 eggs from 438 clutches across four bird species [26,39,40]. Our dataset also spans a wide geographic area, including Albania, Algeria, Belarus, France, Georgia, Germany, Greece, Hungary, Israel, Lithuania, Poland, Romania, Russia, Slovakia, Spain, Tunisia, Turkmenistan, and the West Bank. This extensive dataset supports XAI methods, enhances the validity of our results, and allows for a broader analysis of pigmentation patterns across multiple species.

### Species classification

We employed the VGG16 model, a well-established CNN, in various training scenarios. Initially, we explored three augmentation strategies to assess their influence on the model’s performance. We incorporated rotation into the augmentation process to enhance the model’s ability to handle objects at various angles. Similarly, we used random cropping to improve the model’s resilience to changes in object placement. We also adjusted contrast and brightness to enhance the model’s accuracy and promote more consistent predictions. We observed consistent results across all augmentation methods, demonstrating the model’s strong ability to generalize. For future experiments, we chose heavy augmentation, as it is likely to produce the most invariant model.

Next, we addressed the common challenge of imbalanced class distributions in biological and biomedical datasets. We experimented with oversampling techniques, using augmentation and balanced sampling, which involved oversampling minority classes and undersampling majority classes. The results highlighted the positive impact of balancing the dataset on evaluation metrics.

Third, we trained and tested the model using images of different sizes. We used three dimensions: 256 × 256 pixels, which represented the largest size with intricate details; 128 × 128 pixels for the second input size; and 64 × 64 pixels for the third size, which significantly reduced the detail. The smallest size provided a coarse representation of the patterned egg image, highlighting only the major features. Despite this reduction in detail, our results remained consistently strong, with only minor decreases in predictions for certain classes. This suggests that the features crucial for classification do not rely solely on fine details or a small number of pixels from high-quality images. Our findings demonstrate that discriminative patterns persist even when resizing images to much smaller dimensions.

### Grad-CAM heatmaps

We applied the Grad-CAM procedure separately to both Block 5 and Block 4 of the VGG16 architecture, which allowed us to identify and interpret the specific contributions of features at different levels of abstraction. By examining the class activation maps produced from these distinct blocks, we gained insights into the hierarchical importance of various image regions for the model’s predictions.

Examining the aggregated heatmaps generated from the final convolution block (Block 5) reveals a significant impact, primarily at the central and blunt end of the egg. Notably, the great gray shrike (the species in row C of Fig 3) exhibits a strong influence on the peripheral area of the images in the final convolution block, which warrants further investigation. Heatmaps from the middle blocks (Blocks 3 and 4) show increased focus on the peripheral areas along the boundary of the egg. Block 4 places slightly more attention on the blunt end. In contrast, the results from Block 2 show visible texture, but no major impact is distinguishable.

Exploring the separate results can provide additional insights into which structures, such as spots, texture, or color, have the greatest influence on the classification process. This analysis aims to identify the specific features that drive the model’s decision-making, helping us better understand the network’s behavior.

### Red-backed shrike

Based on Block 4 and Block 5, we can infer that the model focuses on both ends of the egg. In Block 4, the attention focuses on the sharp end of the egg, while Block 5 focuses on the opposite, blunt end. Block 5 indicates regions with spots, mainly within the characteristic ring surrounding the blunt end. Surmacki et al. also highlighted the area known as the “crown” in their study on the relationship between red-backed shrike eggshell patterns and egg size [26]. Their findings suggest that features like the crown pattern and pigmentation traits, including contrast differences between the two ends of the egg, have low reproducibility. Miksik et al. note that protoporphyrin IX levels vary significantly in red-backed shrike eggs, potentially influencing XAI’s attention [18]. Positive SHAP values appear in areas corresponding to spots in the egg’s central region, with the greatest impact linked to spots with moderate contrast, rather than the darkest. We highlight the importance of these positive SHAP values, as high-contrast spots may result from environmental factors. The red-backed shrike usually lays five eggs, one per day, exposing each egg to different environmental influences during its formation [41]. SHAP’s focus on spots of moderate contrast suggests that some patterns may be independent of environmental influences and could serve as unique identifiers for the female.

Conversely, SHAP results reveal a positive impact concentrated near the object’s border, indicating the importance of shape in the decision-making process. DeepExplainer consistently identifies specific spots, which are typically located within the major impact area of the Block 4 Grad-CAM heatmap.

### Lesser gray shrike

In most samples from the lesser gray shrike species, SHAP values concentrate in the egg’s peripheral area, suggesting that the overall shape plays a crucial role in this class. This finding was further supported by quantifying pixels with positive values. Additionally, SHAP values show that regions tend to have more impact than specific spots, indicating a distinctive overall pattern rather than individual spots for this species. More pixels with positive values appeared in cluster_2 than in clusters with lower numbers, further confirming this observation. While Grad-CAM results for this species may seem inconsistent, they consistently focus on the patterned part of the egg, emphasizing its general significance over other features. We suggest that in lesser gray shrikes, individual-specific signatures are encoded in the spatial arrangement of pigment patches, similar to the signatures described for common crane (*Grus grus*) eggs [42]. The eggs of the lesser gray shrike have a distinctive greenish background that sets them apart from other *Lanius* species. This makes it easy to identify lesser gray shrike eggs in oological collections and likely prevents birds from accepting parasitic eggs—unless it involves intraspecies parasitism. In such cases, the female’s unique blot pigment pattern in specific areas of the egg may determine whether it is accepted or rejected from the nest.

### Great gray shrike

SHAP results strongly suggest that the egg’s background color significantly influences the classification of the great gray shrike. The darkest pigmentation spots also have a significant impact, both observable and quantifiable. Overall, all SHAP results consistently highlight that the most patterned and dark-spotted areas are crucial in classifying this species.

In the Grad-CAM evaluation, the model focuses slightly more on the blunt end of the egg. However, the overall trend shows attention across the entire object, without pinpointing any specific region, further confirming the impact of the eggshell’s background color.

The great gray shrike is the largest species in the genus *Lanius*. Compared to other members of the genus, it builds the highest and best-concealed nests and displays remarkable courage in actively defending them [43]. Consequently, we hypothesize that this species faces the least evolutionary pressure to develop complex individual egg signatures. Instead, the egg’s background color may be sufficient for intraspecies communication between the sexes.

### Woodchat shrike

The Grad-CAM heatmaps for this class show a remarkable focus, concentrating on small areas of the eggs. This intensity matches the SHAP results, where only a few regions with strong values appear in the analysis. Intriguingly, even in eggs with many spots, the model seems to base its decision on just a few. Further investigation is needed to determine whether size, shape, or color plays a key role in this context.

Studies on red-backed shrike egg characteristics suggest that environmental factors primarily influence egg size and shape, rather than shell pigmentation [26]. Consequently, eggshell pigmentation may be a heritable trait, making it more susceptible to evolutionary changes compared to environmental factors. This trait could play a key role in avoiding brood parasitism and, as such, may be shaped by natural selection. However, aside from studies on red-backed shrike eggs, similar relationships have not been explored in other *Lanius* species. Our study, which includes four species from this genus, suggests that lesser gray shrikes and great gray shrikes focus on developing unique eggshell backgrounds. In contrast, red-backed shrikes and woodchat shrikes depend on specific pigment patterns and spatial arrangements to identify their eggs. These differences may result from various biological factors specific to each species, such as nesting periods, individual body mass, nest height and concealment, and the presence or abundance of predators and nest parasites in breeding habitats.

### Misclassifications

The most common misclassifications occurred between red-backed shrike and woodchat shrike species. We observed frequent misclassifications in both directions. The CNN assigned probabilities for these misclassified instances ranging from 51% to 98%. This range reflects varying levels of model confidence, from uncertain decisions (around 51%) to highly confident yet incorrect classifications (up to 98%).

Grad-CAM heatmaps show focused attention on the spotted areas of the eggs. The SHAP results reveal a noticeable mix of positive and negative values, which contrasts with the true positive classification XAI results, where SHAP values show more consistency. In the misclassified samples, we observe that specific spots have a major impact, highlighting their importance over the shape of the border or the characteristics of the blunt or pointy ends. Conversely, it appears that the model considered the shape of the spots rather than their color when making decisions.

The classifier’s second most common error is predicting lesser gray shrike for images of woodchat shrike. Grad-CAM heatmaps show that the model focuses on patterned areas near the blunt end in these cases. In most instances, positive SHAP values highlight specific spots as key to the classification. The ambiguity of these spots likely caused the misclassification, as the classifier’s probabilities ranged from 53% to 75%.

### Insights and prospects

As image complexity increases, interpretation becomes much more difficult. The gap in complexity between simple image classification tasks and the detailed analyses required for specific biological questions is vast. In general image interpretation, SHAP DeepExplainer provides clear insights into how individual pixels or features influence model decisions. For instance, in tasks like distinguishing between storks and herons, SHAP easily shows that positive values link to areas with long red beaks and legs, helping to explain the model’s predictions. This simple correlation simplifies interpretation, offering a clear path to understanding why the model makes certain classifications. In contrast to simpler tasks, analyzing the contributions of individual features in more complex scenarios requires deeper analysis.

In this study, we utilized a CNN to develop a model for classifying shrike eggs from museum collections, unlike Chen et al., who applied CNNs to identify poultry eggs [40]. We did not use egg dimensions, as Surmacki et al. did [26], though we collected these measurements and plan to include them in future papers. Inspired by another stimulating research [6], we aimed to address the challenge of identifying individual maternal signatures on the eggshell surface, similar to Gómez et al.’s work with the Eurasian coot (*Fulica atra*) using SpotEgg, an image processing software [39]. The authors concluded that evaluating egg phenotypes requires considering all factors—color, pattern, shape, and size—simultaneously. At the same time, Surmacki et al. [26] pointed out the repetitive nature of egg patterns, along with variations in egg size.

We used machine learning models with explainability techniques to identify the specific features that distinguish each species. While the model performed well in predicting species, we found it difficult to identify clear characteristic features. Nonetheless, our investigation revealed general trends, such as the model’s focus on dark or light spots and peripheral areas of the egg. To improve our study, we plan to experiment with handcrafted features specific to characteristic areas of the egg. With further development, our approach could not only enhance our understanding of how birds perceive eggs but also help organize oological collections. In the future, CNNs are expected to verify the species affiliation of eggs in oological collections worldwide. However, researchers have primarily focused on brood parasitism [7,44,45], CNNs are increasingly used for egg classification and detecting parasitic eggs. Recently, Marini et al. [46] conducted an extensive inventory of about 5 million eggs (roughly 1.97 million egg sets) stored in museums worldwide. These collections, built over the past 250 years, peaked in the 19th and early 20th centuries. Afterward, egg collection became restricted and eventually banned in many regions. Today, analyzing eggs from existing collections offers significant and unique research opportunities [47,48]. However, many stored eggs or egg sets lack clear or legible labels. For example, in Europe, many stored eggs need reidentification (verification) due to lost catalogs from the war period. CNNs can improve oological studies by enhancing identification accuracy and increasing the number of observations for specific bird species.

## Conclusion

We conclude the present results of the investigation indicate that the VGG16 deep learning classification model can distinguish between the four *Lanius* species. The model achieved an accuracy of 0.957 ± 0.015 with input images of size 256 × 256 pixels, which is promising. However, we aim to refine the model further by incorporating a broader spectrum of species during training. Unfortunately, our attempt to utilize XAI methods to uncover pigmentation patterns for species-wide identification signatures produced inconclusive results. In the future, we hope to identify correlations between features associated with individual maternal signatures on the eggshell surface. To achieve this, we plan to apply a hybrid approach that integrates classical image processing techniques, which focus on hand-crafted and more comprehensible features, with deep learning methods. Overall, our approach offers a new way to study how animals perceive signals encoded in eggs.

## Materials and methods

In this study, we aimed to apply XAI methods to uncover pigmentation patterns that represent species-wide identification signatures. We established a classification model using a CNN to classify four species of the shrike family (Laniidae): red-backed shrike (*Lanius collurio*), lesser gray shrike (*L. minor*), great gray shrike (*L. excubitor*), and woodchat shrike (*L. senator*). We obtained the image dataset from several museum collections. Collecting eggshell data from bird species is challenging due to ethical considerations that often restrict or prevent sampling from wildlife populations. Consequently, ethical review boards rarely approve large-scale data collection from live specimens, making museum collections a vital resource for avian egg research. Furthermore, we augmented and resized digital images to train the classifier. We selected the VGG16 machine learning model for the classification task, and it produced promising results. Its compatibility and ease of use allowed us to apply XAI techniques, including Grad-CAM and SHAP, to interpret the model’s decisions.

### Data collection, digital photography, and shrike species

We photographed a total of 2236 eggs from 438 nests across four species of the shrike family, Laniidae. This included 1304 eggs from 267 nests of the red-backed shrike (*L. collurio*), 214 eggs from 41 nests of the lesser gray shrike (*L. minor*), 376 eggs from 67 nests of the great gray shrike (*L. excubitor*), and 342 eggs from 62 nests of the woodchat shrike (*L. senator*). We excluded any nest (clutch) with unclear details, such as uncertain collection year, location, or maternal lineage. This approach also prevented analyzing multiple clutches from the same female.

**Fig 5:**
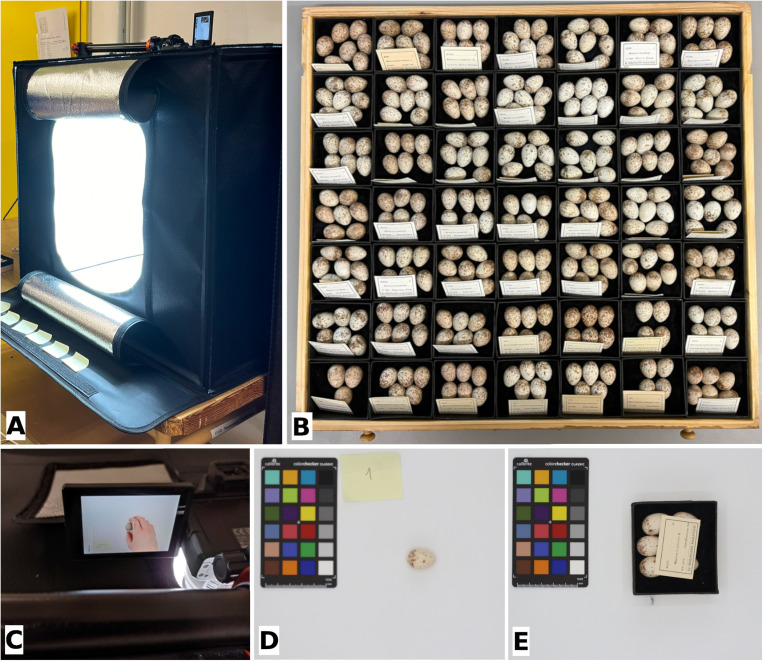
Stages of taking photographs. Presentation of (A) mounted equipment during one of the photo sessions; (B) drawer with great gray shrike eggs from the Senckenberg Natural History Collections Dresden, Germany; (C) photographing procedure of a single shrike egg; (D) single red-backed shrike egg with the standardized color chart Calibrite® ColorChecker Classic Mini and a label showing the clutch number; and (E) full clutch of red-backed shrike eggs with the standardized color chart Calibrite® ColorChecker Classic Mini and a genuine collection label.

We took photographs at six sites housing oological collections: five in Poland and one in Germany. These sites are: (1) Świętokrzyski National Park, Bodzentyn, Poland; (2) Count Antoni Ostrowski Museum in Tomaszow Mazowiecki, Poland; (3) Museum and Institute of Zoology Polish Academy of Sciences Research Station, Palmiry, Poland; (4) Museum of Natural History, University of Wrocław, Poland; (5) Jacek Malczewski Museum in Radom, Poland; and (6) Senckenberg Natural History Collections Dresden, Germany (Fig 5b). Table 5 shows the number of clutches and eggs per species. All eggs used in this study were blown eggs stored in closed cabinets, with no access to light, which prevented any changes in shell color. After removing the eggs from the display case, we measured the mass, length, and width of each shell before proceeding with the photography.

**Table 5:** Number of clutches and eggs (in brackets) per species for all six oology collections.

Shrike species	Localization of oology collections						Total for species
	Bodzentyn^1^	Tomaszow Mazowiecki^2^	Palmiry^3^	Wroclaw^4^	Radom^5^	Dresno^6^	
Red-backed shrike	25 (88)	108 (520)	27 (127)	49 (221)	9 (47)	50 (301)	**268 (1304)**
Lesser gray shrike	–	1 (5)	6 (28)	10 (36)	1 (5)	23 (140)	**41** **(214)**
Great gray shrike	–	1 (2)	–	12 (51)	2 (5)	52 (318)	**67** **(376)**
Woodchat shrike	–	3 (14)	1 (2)	7 (27)	1 (2)	50 (297)	**62** **(342)**
**Total for place**	**25 (88)**	**113 (541)**	**34 (157)**	**78 (335)**	**13 (59)**	**175 (1056)**	

^1^The Swietokrzyski National Park oology collection includes Shrike specimens originating from Poland.

^2^The Count Antoni Ostrowski Museum in Tomaszow Mazowiecki oology collection includes Shrike specimens originating from Poland.

^3^The Museum and Institute of Zoology Polish Academy of Sciences Research Station oology collection includes Shrike specimens originating from Belarus and Poland.

^4^The Museum of Natural History, University of Wroclaw, oology collection includes Shrike specimens originating from Albania, Poland, Spain, and Tunisia.

^5^The Jacek Malczewski Museum in Radom oology collection includes Shrike specimens originating from Belarus, Lithuania, and Poland.

^6^The Senckenberg Natural History Collections Dresden oology collection includes Shrike specimens originating from Algeria, France, Georgia, Germany, Greece, Hungary, Israel, Romania, Russia, Slovakia, Spain, Turkmenistan, and the West Bank.

We took all photos using a Canon® EOS M50 Mark II camera with a Canon® Zoom Lens EF-M 15–45 mm and a Hoya® CIR-PL UV filter. We used the same settings for each photo: aperture f/11, shutter speed 1/60, and ISO 200. Before photographing, we placed the eggs in a shadeless tent, the GlareOne® LED Cube 60 (Fig 5a), along with a standardized color chart, the Calibrite® ColorChecker Classic Mini (Fig 5d,e). We used a tripod and remote control for stability during the process. Each egg was photographed twice: after the first shot, we rotated the egg 180 degrees. In total, we took 4472 photos (2 photos for each of the 2236 eggs).

### Dataset formation

We used our method to automatically create an image database from the collected photographs. Although we took the images following a repetitive scheme, preprocessing [49,50] was essential for AI-driven analysis. We applied an automated method to extract the eggs, cropping them out and organizing them into file-folder structures that efficiently fed the images to the AI model. We manually pruned the dataset to remove artifacts, eggs marked with tags, extraneous objects, and cracked eggs. Ultimately, we curated a dataset of 3260 images, ready for use in CNN training.

### Deep learning model and implementation

**Fig 6:**
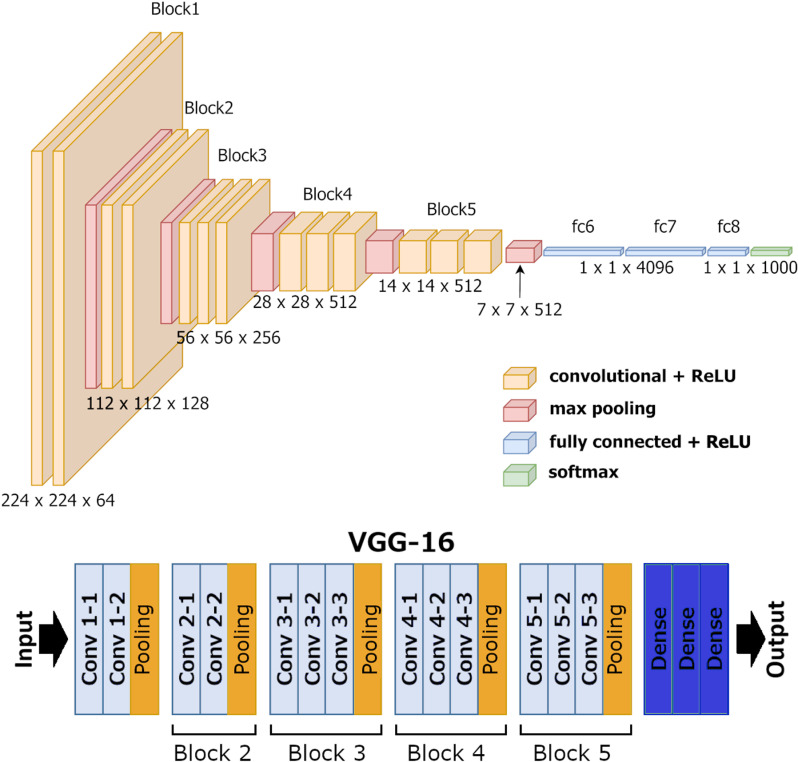
VGG16 model architecture with explicit designations of Blocks from 2 to 5.

In our experiments, we used the widely recognized VGG16 model [51], adapting its input and output layers to meet our specific objectives (Fig 6). This architecture has proven efficient in a variety of applications, from microscopy scans [52–54] to satellite images [55]. Notably, we initialized the model’s weights randomly. For optimization, we primarily used the stochastic gradient descent (SGD) optimizer with a learning rate of 1e-6 and a momentum value of 0.9. We chose the “categorical crossentropy” loss function, a standard option for classification tasks. We set the split for train/validation/test subsets to 0.5/0.2/0.3. Throughout all experiments, we used a consistent batch size of 8.

We implemented the model, along with the training and inference processes, in Python using the Keras framework with TensorFlow as the backend. The open-source nature of the model made it easily accessible, and we obtained it from the publicly available GitHub repository [56]. This Python-based implementation provided flexibility and leveraged Keras and TensorFlow’s capabilities for efficient neural network operations. The VGG16 architecture, proven effective in image classification tasks, was modified to meet our specific classification goals. We used SGD with the categorical cross-entropy loss function, following established practices in training deep neural networks.

### Data augmentation

In situations with very few examples, data augmentation can improve the quality of the results. In our study, we used the Albumentations package [57] to modify the images. To systematically introduce diversity, we applied three distinct augmentation strategies: simple, medium, and heavy.

#### Simple.

The simple augmentation involved only vertical and horizontal flips, with image rotation restricted to 180 degrees. This type of augmentation has a minimal impact on image content since it does not affect the pixel values. Nevertheless, it can enhance the model’s training capabilities by making it agnostic to the left-right orientation of the egg.

#### Medium.

In addition to simple augmentation, this strategy included further distortions such as image rotation and random cropping. These transformations applied multiple geometric changes, altering the position of objects within the images.

#### Heavy.

Along with simple and medium augmentations, the heavy option provided additional distortions in brightness and contrast to make the model agnostic to light conditions and automatic camera white balance adjustments. The Albumentation functions used in the heavy augmentation option included: VerticalFlip (*p* = 0.5), HorizontalFlip (*p* = 0.5), Rotate (limit=[0,180], *p* = 0.5), RandomSizedCrop (*p* = 0.5), RandomBrightnessContrast (*p* = 0.5), and RandomGamma (*p* = 0.5).All of the above strategies improve the generalization of knowledge. However, we designed each strategy to cater to different levels of augmentation intensity, allowing us to explore the impact of augmentation on model performance across a range of complexities.

Furthermore, we used augmentation techniques to balance the dataset. Notably, we applied the most augmentation to the least populated class to address the imbalance effectively. This approach is crucial for maintaining high accuracy and avoiding misclassifications, especially in applications where each class or category holds equal importance.

The open-source Albumentations package provided a solid foundation for applying these augmentation techniques efficiently. By using these strategies, we enhanced the flexibility of our experimental setup, improving the model’s ability to learn from a limited set of examples. This augmentation process was crucial in overcoming data scarcity challenges, making our trained model more robust and better at generalizing.

### XAI (Explainable Artificial Intelligence)

**XAI** (**Ex**plainable **A**rtificial **I**ntelligence) aims to demystify complex machine learning models by making their decision-making processes understandable to humans [58]. In our study, we used XAI methods, such as Grad-CAM and SHAP, to clarify how our model reaches conclusions. These methods highlight influential regions in images, enabling researchers and users to interpret and trust the model’s outputs. This promotes transparency in artificial intelligence systems.

### Grad-CAM (Gradient-weighted Class Activation Mapping)

**Grad-CAM** (**Grad**ient-weighted **C**lass **A**ctivation **M**apping) [59] is a powerful XAI technique that visualizes the regions of an image that strongly influence a deep learning model’s decision. Grad-CAM operates on the final convolutional layer of a CNN, just before the global average pooling and fully connected layers. This layer retains spatial information essential for understanding the model’s decision-making. The process starts by computing the gradients of the target class with respect to the feature maps of the convolutional layer. These gradients represent the importance of each feature map in the decision for the target class. By averaging the gradients for each feature map, we obtain the importance weight for each channel. Next, we compute a weighted combination of the feature maps using these importance weights. This generates a class activation map that highlights the regions of the input image that most influenced the model’s prediction for the target class. To create a visual interpretation, we rescale the class activation map and overlay it onto the original input image. This overlay emphasizes the critical areas that influenced the model’s decision, providing a clear and interpretable visual representation of the model’s attention. Grad-CAM does not require any architectural changes to the original model, making it a versatile and widely applicable XAI tool. Its transparency and ability to highlight relevant image regions make it valuable in various domains, such as medical imaging, where understanding the model’s focus is crucial for building trust and insight into decision-making. Overall, Grad-CAM helps bridge the gap between the powerful, complex nature of deep learning models and the need for human-understandable explanations in AI systems.

**Fig 7:**
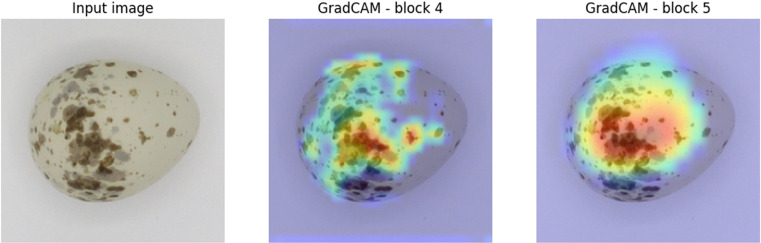
Example of Grad-CAM heatmaps from Blocks 4 and 5, with overlaid major impact (leftmost image), indicating the border of an area in the third quartile (over 75% of the maximum value) shown (Block 4—yellow, Block 5—red). The rightmost image shows the major impact overlaid on the SHAP result.

In our study, we generated Grad-CAM heatmaps from two key blocks within the VGG16 architecture: Block 4 and Block 5 (Fig 7). These blocks play crucial roles as pivotal stages in the model’s hierarchical feature extraction process. We focused Grad-CAM visualization on these later blocks because they capture high-level features that are essential for understanding the model’s decision-making. We intentionally omitted earlier blocks, as they capture low-level features that may not offer meaningful insights into the specific regions influencing the model’s predictions. For Grad-CAM, the final convolutional layer (Block 5) is crucial for capturing high-level features before global average pooling and the fully connected layers. Similarly, Block 4, an intermediate convolutional layer, retains essential spatial information that contributes to the model’s decision-making process.

### SHAP DeepExplainer (SHapley Additive exPlanations)

**Fig 8:**
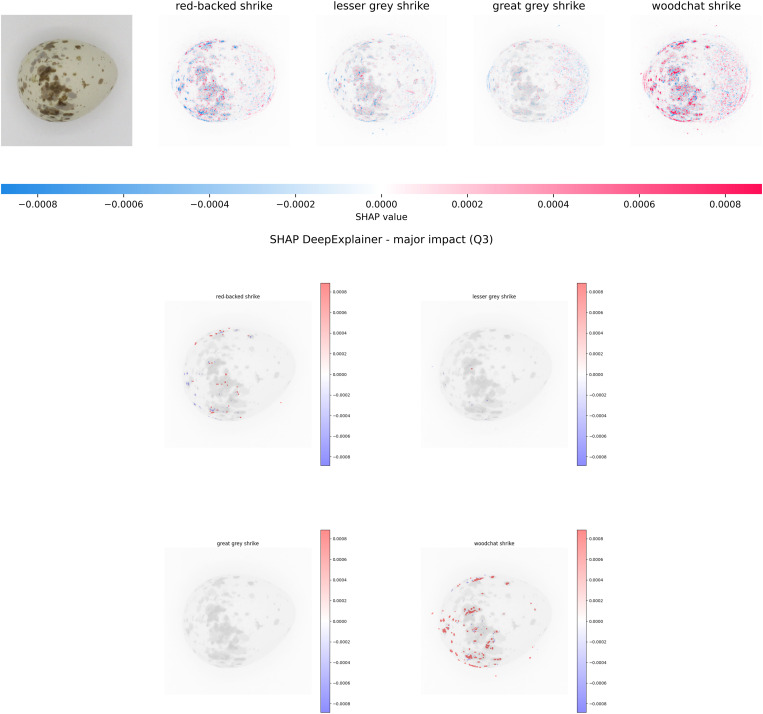
Example of SHAP DeepExplainer results with Shapley values for all considered classes, correctly classified as woodchat shrike. Alongside the standard output, we show the major impact—pixels with values in the third quartile—for the same example.

**SHAP DeepExplainer** (**SH**apley **A**dditive ex**P**lanations) [60] is a pivotal method in XAI that explains how machine learning models make decisions. SHAP values, based on Shapley values from cooperative game theory, assign importance to each “player” in a coalition (example in Fig 8). In image analysis, each pixel in an image acts as a player. The SHAP DeepExplainer is a specialized implementation within the SHAP framework, designed for deep learning models, particularly those with CNN architectures commonly used in image analysis. It helps us understand the contributions of individual image regions in deep neural networks by providing Shapley values for each pixel in an image. To compute these values, the algorithm systematically perturbs combinations of pixels in the input image and observes how the model’s output changes. The algorithm calculates the Shapley values based on the average marginal contributions of each pixel across all possible combinations. These values represent each pixel’s contribution to the difference between the model’s prediction for a specific instance and the average prediction across all cases. Positive values indicate a positive impact, while negative values suggest a negative influence.

SHAP’s adaptability allows it to break down a model’s decision for a given image instance. This approach enhances transparency and interpretability, providing insights into how specific regions influence predictions.

In our experiments, we focused on positive Shapley values with a positive impact in images with correctly predicted classes, where each class represented one of the four *Lanius* species. We treated the number of pixels with values in the second and third quartiles as a distinctive feature. Additionally, we calculated the pixels around the dilated edge with positive values.

### Evaluation metrics

Evaluation is essential to determine the accuracy of classifying examples from the test set. We used four metrics to evaluate the model: Precision, Recall, Accuracy, and F1 score. Precision measures the ratio of true positives (TP) to all predicted positives: Precision = TP/(TP + FP). Recall shows the probability of correct classification: Recall = TP/(TP + FN). The F1 score is the harmonic mean of Precision and Recall. Accuracy represents the fraction of correct classifications: Accuracy = (TP + TN)/(TP + TN + FP + FN). For all metrics, values closer to 1 indicate better results, where TP = True positive, FP = False positive, TN = True negative, and FN = False negative.

## Supporting information

S1 File Class predictions. (ZIP)

## References

[1] Stoddard MC, Marshall KLA, Kilner RM. Imperfectly camouflaged avian eggs: artefact or adaptation? Avian Biol Res. 2011;4(4):196–213.

[2] Stoddard MC, Kupán K, Eyster HN, Rojas-Abreu W, Cruz-López M, Serrano-Meneses MA, et al. Camouflage and clutch survival in plovers and terns. Sci Rep. 2016;6:32059. 27616020 10.1038/srep32059 PMC5018847

[3] Troscianko J, Wilson-Aggarwal J, Stevens M, Spottiswoode CN. Camouflage predicts survival in ground-nesting birds. Sci Rep. 2016;6(1):19966. 26822039 10.1038/srep19966 PMC4731810

[4] Yang C, Liang W, Cai Y, Shi S, Takasu F, Møller AP, et al. Coevolution in action: disruptive selection on egg colour in an avian brood parasite and its host. PLOS ONE. 2010;5(5):e10816. doi: 10.1371/journal.pone.0010816 20520815 PMC2877083

[5] Stevens M, Troscianko J, Spottiswoode CN. Repeated targeting of the same hosts by a brood parasite compromises host egg rejection. Nat Commun. 2013;(1):2475. doi: 10.1038/ncomms3475 24064931 PMC3791459

[6] Stoddard MC, Kilner RM, Town C. Pattern recognition algorithm reveals how birds evolve individual egg pattern signatures. Nat Commun. 2014;5(1):4117. doi: 10.1038/ncomms5117 24939367

[7] Dixit T, Lund J, Fulford AJC, Apostol AL, Chen K-C, Tong W, et al. Chase-away evolution maintains imperfect mimicry in a brood parasite-host system despite rapid evolution of mimics. Nat Ecol Evol. 2023;7(12):1978–82. doi: 10.1038/s41559-023-02232-4 37872417 PMC10697838

[8] Maurer G, Portugal SJ, Cassey P. Review: an embryo’s eye view of avian eggshell pigmentation. J Avian Biol. 2011;42(6):494–504.

[9] Chen X, Li X, He Z, Hou Z, Xu G, Yang N, et al. Comparative study of eggshell antibacterial effectivity in precocial and altricial birds using *Escherichia coli*. PLOS ONE. 2019;14(7):e0220054. doi: 10.1371/journal.pone.0220054 31339918 PMC6655735

[10] Birkhead TR. Behavioural adaptations to high density nesting in the common guillemot *Uria aalge*. Anim Behav. 1978;26:321–31.

[11] Quach L, Miller AE, Hogan BG, Stoddard MC. Egg patterns as identity signals in colonial seabirds: a comparison of four alcid species. J Exp Zool B Mol Dev Evol. 2021;336(8):595–605. doi: 10.1002/jez.b.22945 32400035

[12] Rosenberger J, Lukaszewicz E, Kowalczyk A, Rzonca Z. Capercaillie (*Tetrao urogallus*) eggshell pigmentation, maculation and thickness. Ornis Fenn. 2018; 95(4):160–70.

[13] Moreno J, Osorno JL. Avian egg colour and sexual selection: does eggshell pigmentation reflect female condition and genetic quality? Ecol Lett. 2003;6(9):803–6.

[14] Reynolds SJ, Martin GR, Cassey P. Is sexual selection blurring the functional significance of eggshell coloration hypotheses? Anim Behav. 2009;78(1):209–15.

[15] Hodges KE, Mortimer NT, Vrailas-Mortimer AD, Sakaluk SK, Thompson CF. Connecting the dots: avian eggshell pigmentation, female condition and paternal provisioning effort. Biol J Linn Soc. 2020; 30(1):114–27. 10.1093/biolinnean/blaa002 PMC7199457 32394988

[16] Gill F, Donsker D, Rasmussen P. IOC World Bird List (v14.1). 2021

[17] McCullough JM, Hruska JP, Oliveros CH, Moyle RG, Andersen MJ. Ultraconserved elements support the elevation of a new avian family, Eurocephalidae, the white-crowned shrikes. Ornithology. 2023;140(3):ukad025.

[18] Miksik I, Holáň V, Deyl Z. Quantification and variability of eggshell pigment content. Comp Biochem Physiol A Physiol. 1994;109(3):769–72.

[19] Takasu F. Co-evolutionary dynamics of egg appearance in avian brood parasitism. Evol Ecol Res. 2003;5(3):345–62.

[20] Lovászi P, Moskát C. Break-down of arms race between the red-backed shrike (*Lanius collurio*) and common cuckoo (*Cuculus canorus*). Behaviour. 2004;141(2):245–62.

[21] Spottiswoode CN, Stevens M. How to evade a coevolving brood parasite: egg discrimination versus egg variability as host defences. Proc R Soc B Biol Sci. 2011;278(1724):3566–73. 10.1098/rspb.2011.0401 PMC3189372 21490019

[22] Lahti DC. Persistence of egg recognition in the absence of cuckoo brood parasitism: pattern and mechanism. Evolution. 2006;60(1):157–68. 16568640

[23] Morelli F, Benedetti Y, Callaghan CT. Ecological specialization and population trends in European breeding birds. Glob Ecol Conserv. 2020; 22:e00996.

[24] Kuczyński L, Antczak M, Czechowski P, Grzybek J, Jerzak L, Zabłocki P, et al. A large scale survey of the great grey shrike *Lanius* excubitor in Poland: breeding densities, habitat use and population trends. Ann Zool Fenn. 2010;47(1):67–78.

[25] Peer BD, McIntosh CE, Kuehn MJ, Rothstein SI, Fleischer RC. Complex biogeographic history of *Lanius* shrikes and its implications for the evolution of defenses against avian brood parasitism. Condor. 2011;113(2):385–94. doi: 10.1525/cond.2011.100066

[26] Surmacki A, Kuczyński L, Tryjanowski P. Eggshell patterning in the red-backed shrike *Lanius collurio*: relation to egg size and potential function. Acta Ornithol. 2006;41(2):145–51.

[27] Gómez J, Liñán‐Cembrano G. SpotEgg: an image‐processing tool for automatised analysis of colouration and spottiness. J Avian Biol. 2017;48(4):502–12.

[28] L’Herpiniere KL, O’Neill LG, Russell AF, Duursma DE, Griffith SC. Unscrambling variation in avian eggshell colour and patterning in a continent-wide study. R Soc Open Sci. 2019;6(1):181269. doi: 10.1098/rsos.181269 30800374 PMC6366205

[29] Johnson EW, McRae SB. Interclutch variability in egg characteristics in two species of rail: Is maternal identity encoded in eggshell patterns? PLOS ONE. 2022;17(1):e0261868. doi: 10.1371/journal.pone.0261868 35025922 PMC8758195

[30] Lakhani P, Sundaram B. Deep learning at chest radiography: automated classification of pulmonary tuberculosis by using convolutional neural networks. Radiology. 2017;284(2):574–82. doi: 10.1148/radiol.2017162326 28436741

[31] Huang WC, Peters MS, Ahlebæk MJ, Frandsen MT, Eriksen RL, Jørgensen B. The application of convolutional neural networks for tomographic reconstruction of hyperspectral images. Displays. 2022;74:102218.

[32] Kim KH, Choi SH, Park SH. Improving arterial spin labeling by using deep learning. Radiology. 2018;287(2):658–66. doi: 10.1148/radiol.2017171154 29267145

[33] Chen MC, Ball RL, Yang L, Moradzadeh N, Chapman BE, Larson DB, et al. Deep learning to classify radiology free-text reports. Radiology. 2018;286(3):845–52. doi: 10.1148/radiol.2017171115 29135365

[34] Ravindran U, Gunavathi C. A survey on gene expression data analysis using deep learning methods for cancer diagnosis. Prog Biophys Mol Biol. 2023;177:1–13. doi: 10.1016/j.pbiomolbio.2022.08.004 35988771

[35] Babichev S, Liakh I, Kalinina I. Applying the deep learning techniques to solve classification tasks using gene expression data. IEEE Access. 2024;12:28437–48.

[36] Temenos A, Temenos N, Kaselimi M, Doulamis A, Doulamis N. Interpretable deep learning framework for land use and land cover classification in remote sensing using SHAP. IEEE Geosci Remote Sens Lett. 2023;20:1–5. doi: 10.1109/lgrs.2023.3251652

[37] Adegun AA, Viriri S, Tapamo JR. Review of deep learning methods for remote sensing satellite images classification: experimental survey and comparative analysis. J Big Data. 2023; 10(1):93.

[38] Korzyńska A, Roszkowiak L, Zak J, Siemion K. A review of current systems for annotation of cell and tissue images in digital pathology. Biocybern Biomed Eng. 2021;41(4):1436–53.

[39] Gómez J, Gordo O, Minias P. Egg recognition: The importance of quantifying multiple repeatable features as visual identity signals. PLOS ONE. 2021;16(3):e0248021. doi: 10.1371/journal.pone.0248021 33661988 PMC7932075

[40] Chen Z, He P, He Y, Wu F, Rao X, Pan J, et al. Eggshell biometrics for individual egg identification based on convolutional neural networks. Poult Sci. 2023;102(4):102540. doi: 10.1016/j.psj.2023.102540 36863120 PMC10006506

[41] Tryjanowski P. A long-term comparison of laying date and clutch size in the red-backed shrike (*Lanius collurio*) in Silesia, southern Poland. Acta Zool Hung. 2002;48(2):101–6.

[42] Höltje H, Mewes W, Haase M, Ornés AS. Genetic evidence of female specific eggshell colouration in the common crane (*Grus grus*). J Ornithol. 2016;157(2):609–17.

[43] Fan L, Gao L, Zhu Z, Zhang X, Zhang W, Zhang H, et al. The grey-backed shrike parents adopt brood survival strategy in both the egg and nestling phases. Avian Res. 2021;12(1):11.

[44] Nhidi W, Aoun NB, Ejbali R. Deep learning-based parasitic egg identification from a slender-billed gull’s nest. IEEE Access. 2023;11:37194–202. doi: 10.1109/access.2023.3267083

[45] Spottiswoode CN, Stevens M. Visual modeling shows that avian host parents use multiple visual cues in rejecting parasitic eggs. Proc Natl Acad Sci U S A. 2010;107(19):8672–6. doi: 10.1073/pnas.0910486107 20421497 PMC2889299

[46] Marini MÂ, Hall L, Bates J, Steinheimer FD, McGowan R, Silveira LF, et al. The five million bird eggs in the world’s museum collections are an invaluable and underused resource. The Auk 2020; 137(4):ukaa036.

[47] Stoddard MC, Yong EH, Akkaynak D, Sheard C, Tobias JA, Mahadevan L. Avian egg shape: form, function, and evolution. Science. 2017;356(6344):1249–54. doi: 10.1126/science.aaj1945 28642430

[48] Birkhead TR, Thompson JE, Biggins JD, Montgomerie R. The evolution of egg shape in birds: selection during the incubation period. Ibis. 2019;161(3):605–18. doi: 10.1111/ibi.12658

[49] Uchida S. Image processing and recognition for biological images. Dev Growth Differ. 2013;55(4):523–49. doi: 10.1111/dgd.12054 23560739 PMC3746120

[50] Neuman U, Korzyńska A, Lopez C, Lejeune M, Roszkowiak Ł, Bosch R. Equalisation of archival microscopic images from immunohistochemically stained tissue sections. Biocybern Biomed Eng. 2013;33(1):63–76.

[51] Simonyan K, Zisserman A. Very deep convolutional networks for large-scale image recognition [Internet]. arXiv; 2015 [cited 2024 Jul 4]. Available from: http://arxiv.org/abs/1409.1556

[52] López C, Callau C, Bosch R, Korzynska A, Jaén J, García-Rojo M, et al. Development of automated quantification methodologies of immunohistochemical markers to determine patterns of immune response in breast cancer: a retrospective cohort study. BMJ Open. 2014;4(8):e005643. doi: 10.1136/bmjopen-2014-005643 25091015 PMC4127922

[53] Lijo J, S SJ. Analysis of U-net and modified vgg16 technique for mitosis identification in histopathology images. In: 2024 International Conference on Wireless Communications Signal Processing and Networking (WiSPNET) [Internet]. 2024 [cited 2024 Jul 3]. p. 1–8. Available from: doi: 10.1109/wispnet61464.2024.10533123

[54] Korzyńska A, Zak J, Siemion K, Roszkowiak L, Pijanowska D. CNN support to diagnostics in Sjögren’s Syndrome. In: Korbicz J, Maniewski R, Patan K, Kowal M, editors. Current Trends in Biomedical Engineering and Bioimages Analysis. Cham: Springer International Publishing; 2020. p. 72–81.

[55] Ankita, Mittal S. Image classification of satellite using VGG16 model. In: 2024 2nd International Conference on Disruptive Technologies (ICDT) [Internet]. 2024 [cited 2024 Jul 3]. p. 401–4. Available from: https://ieeexplore.ieee.org/abstract/document/10489685

[56] Iakubovskii P. qubvel/classification_models [Internet]. 2024 [cited 2024 Jul 3]. Available from: https://github.com/qubvel/classification_models

[57] Buslaev A, Iglovikov VI, Khvedchenya E, Parinov A, Druzhinin M, Kalinin AA. Albumentations: fast and flexible image augmentations. Information. 2020;11(2):125.

[58] Nazir S, Dickson DM, Akram MU. Survey of explainable artificial intelligence techniques for biomedical imaging with deep neural networks. Comput Biol Med. 2023; 156:106668. 36863192 10.1016/j.compbiomed.2023.106668

[59] Selvaraju RR, Cogswell M, Das A, Vedantam R, Parikh D, Batra D. Grad-CAM: visual explanations from deep networks via gradient-based localization. In: 2017 IEEE International Conference on Computer Vision (ICCV) [Internet]. 2017 [cited 2024 Jul 3]. p. 618–26. Available from: https://ieeexplore.ieee.org/document/8237336.

[60] Lundberg SM, Lee SI. A unified approach to interpreting model predictions. In: Advances in Neural Information Processing Systems [Internet]. Curran Associates, Inc.; 2017 [cited 2024 Jul 3]. Available from: https://proceedings.neurips.cc/paper/2017/hash/8a20a8621978632d76c43dfd28b67767-Abstract.html.

