# Supplementary material for: Can explainable AI classify shrike (Laniidae) eggs by uncovering species-wide pigmentation patterns?
Source: PLoS One. 2025 May 2;20(5):e0321532. doi: 10.1371/journal.pone.0321532 (PMC12047758; doi:10.1371/journal.pone.0321532)

IMG_1519.JPG SHAP DeepExplainer major impact (Q3)

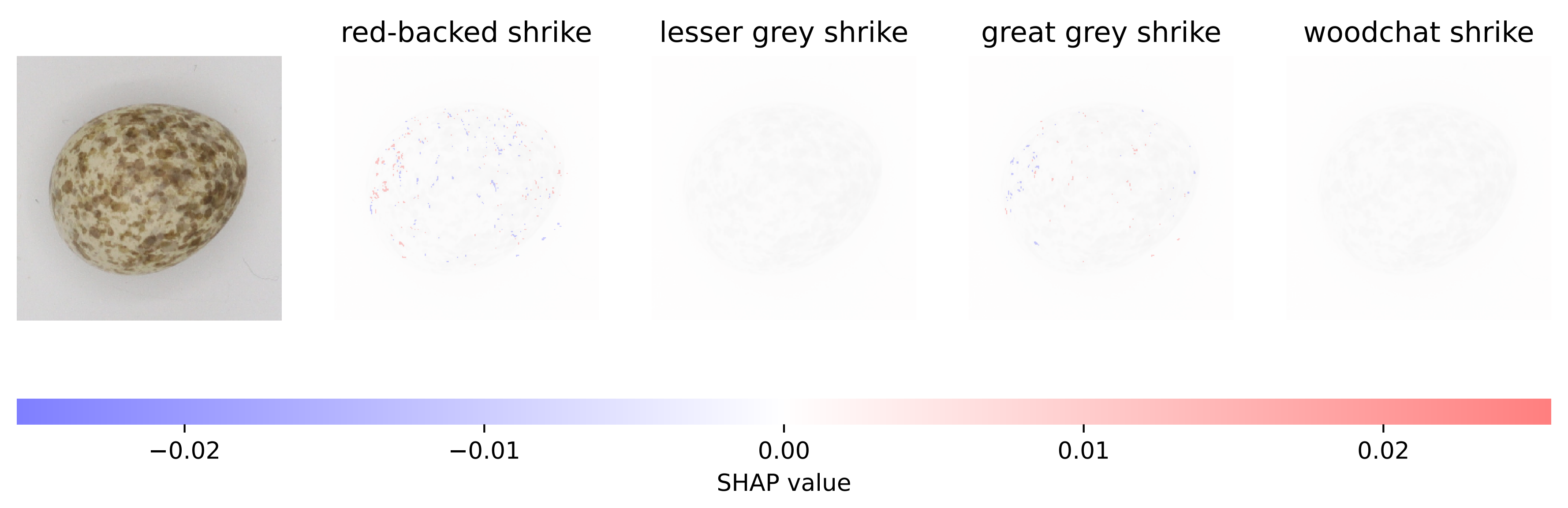

IMG_1522.JPG SHAP DeepExplainer major impact (Q3)

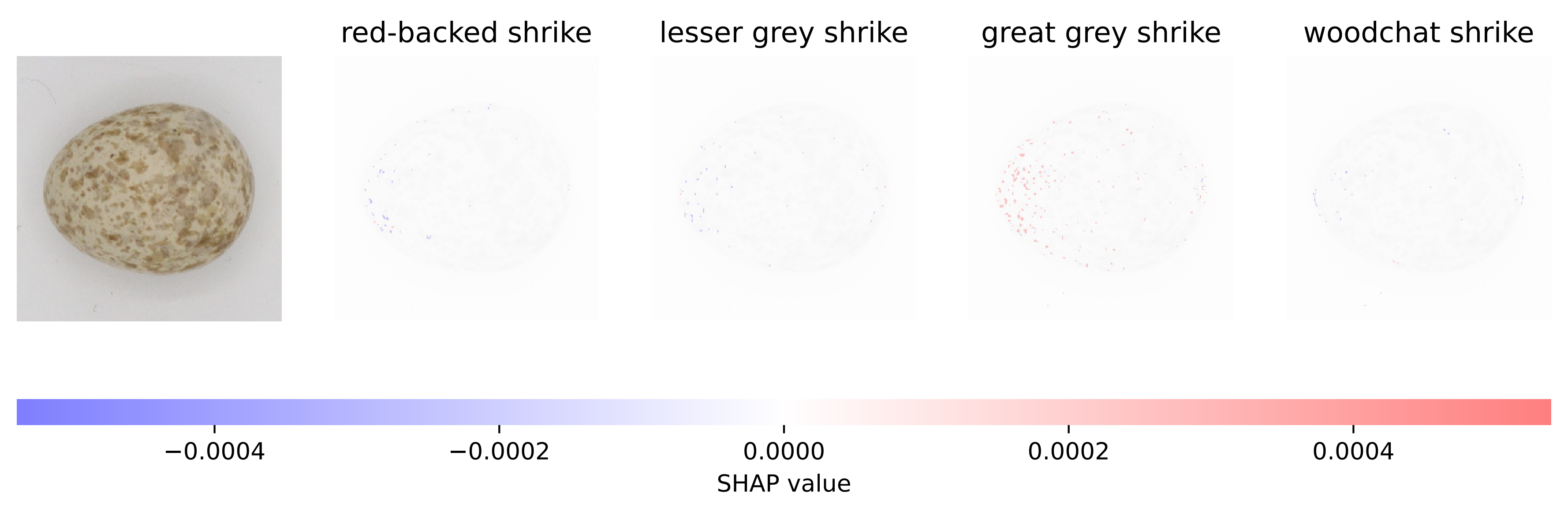

IMG_0968.JPG SHAP DeepExplainer major impact (Q3)

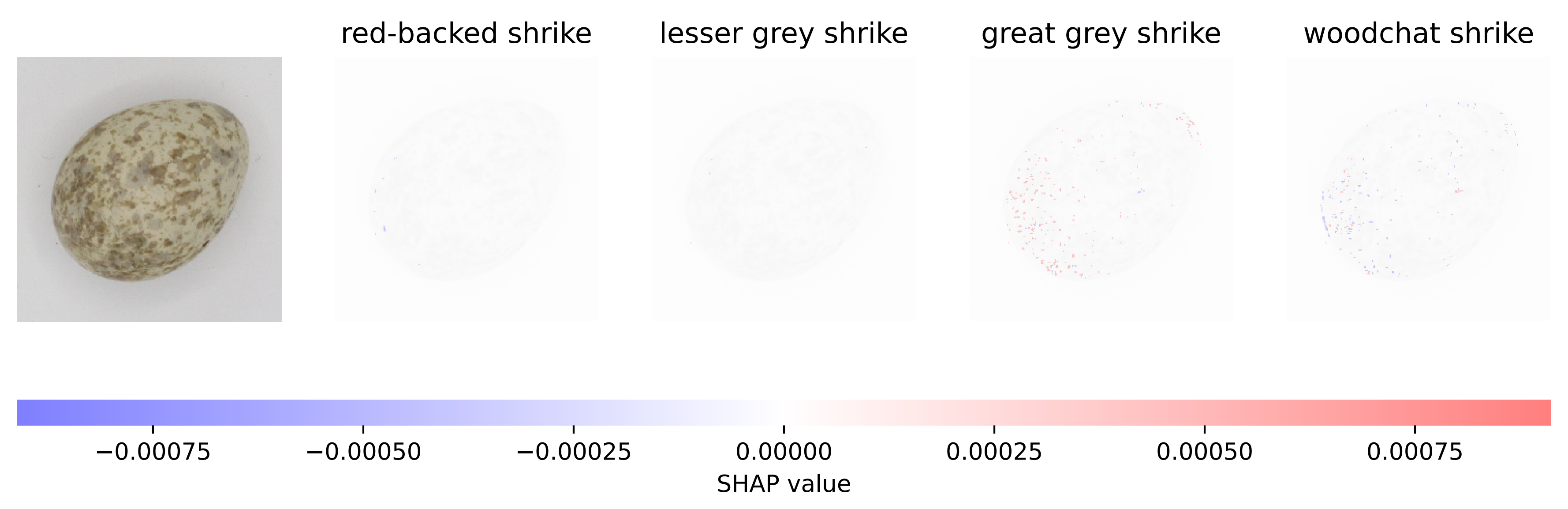

IMG_1104.JPG SHAP DeepExplainer major impact (Q3)

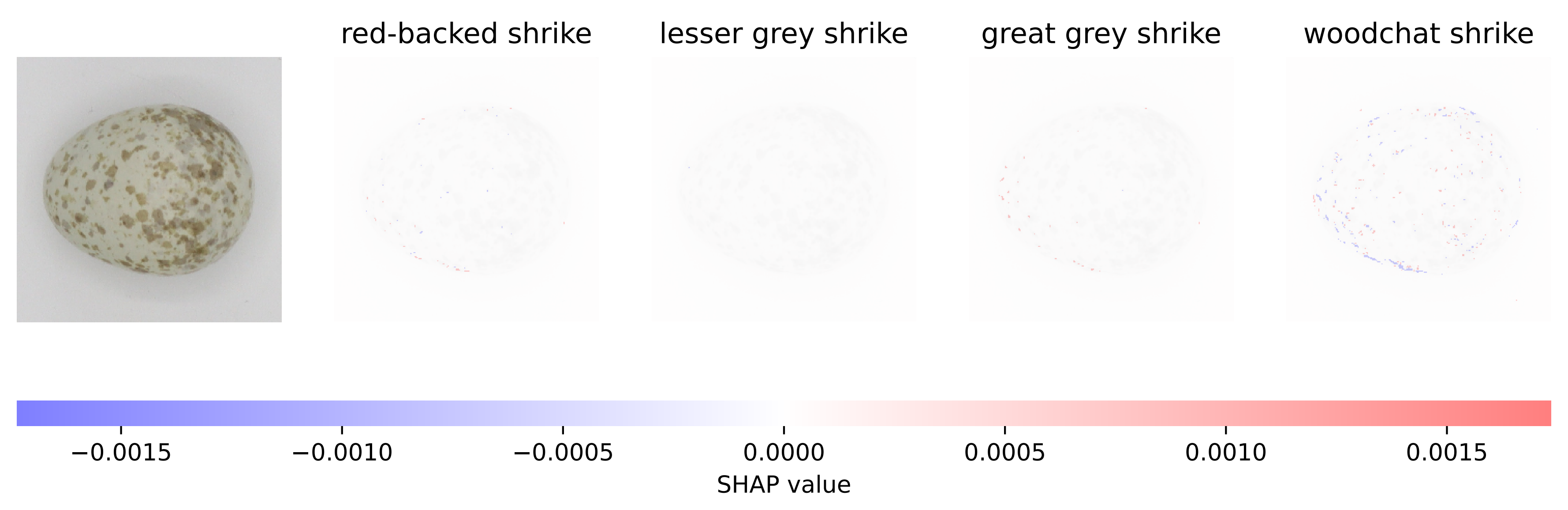

IMG_1126.JPG SHAP DeepExplainer major impact (Q3)

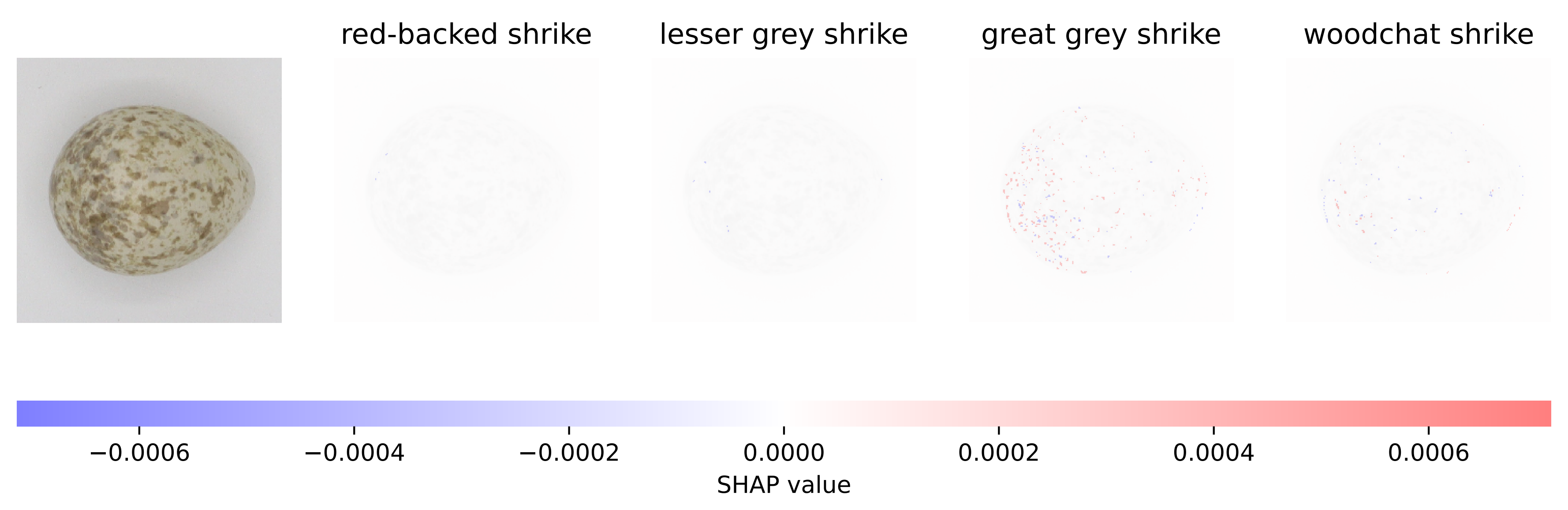

IMG_1187.JPG SHAP DeepExplainer major impact (Q3)

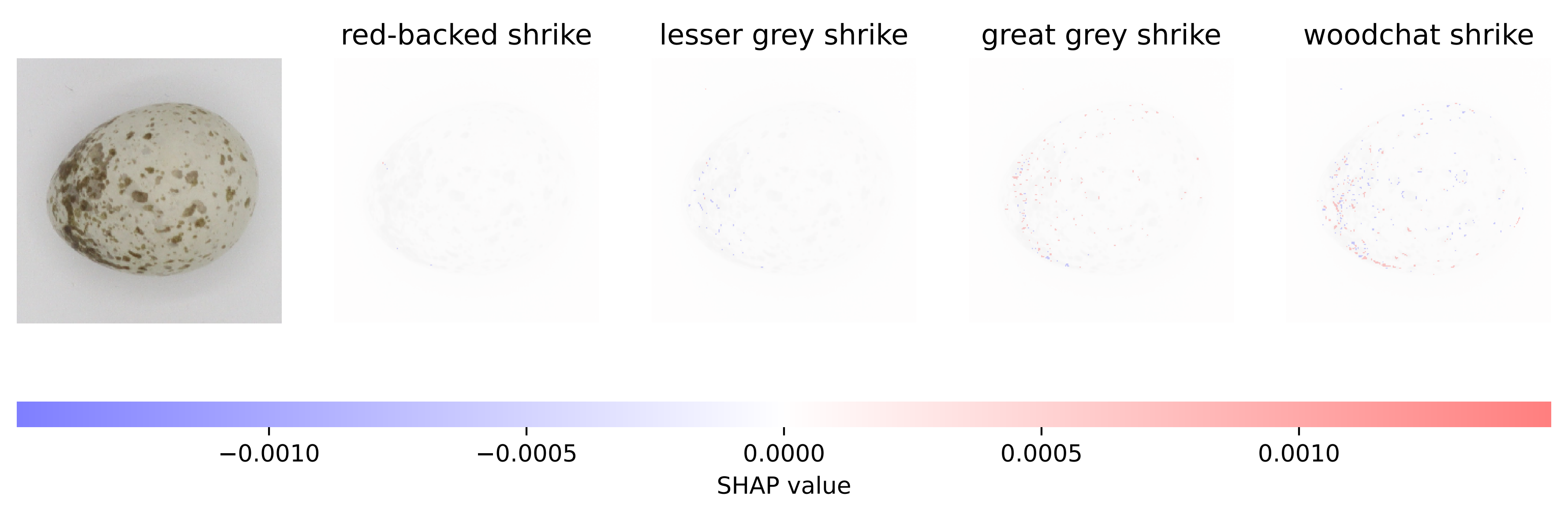

IMG_1211.JPG SHAP DeepExplainer major impact (Q3)

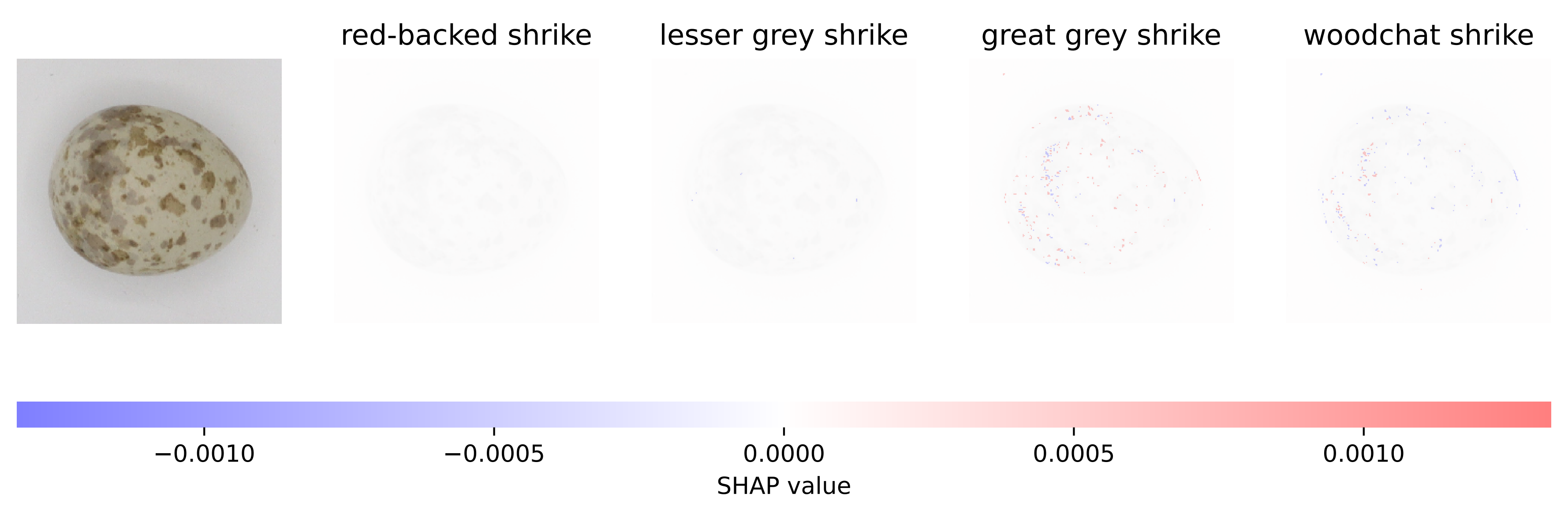

IMG_1312.JPG SHAP DeepExplainer major impact (Q3)

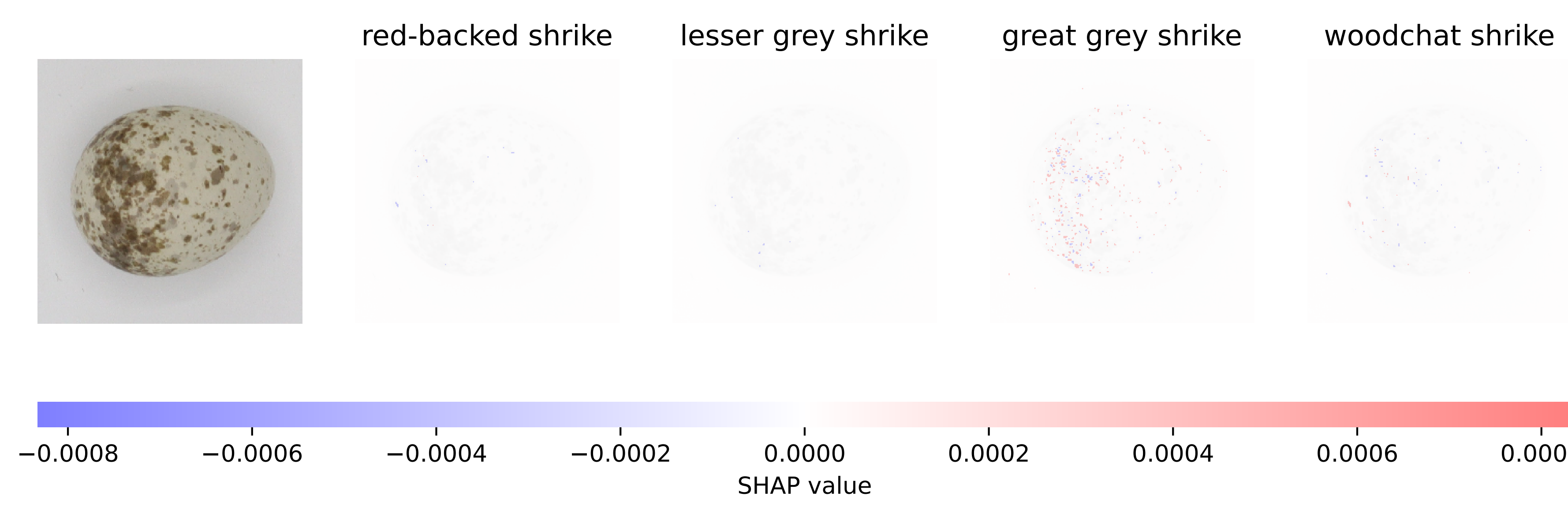

IMG_1313.JPG SHAP DeepExplainer major impact (Q3)

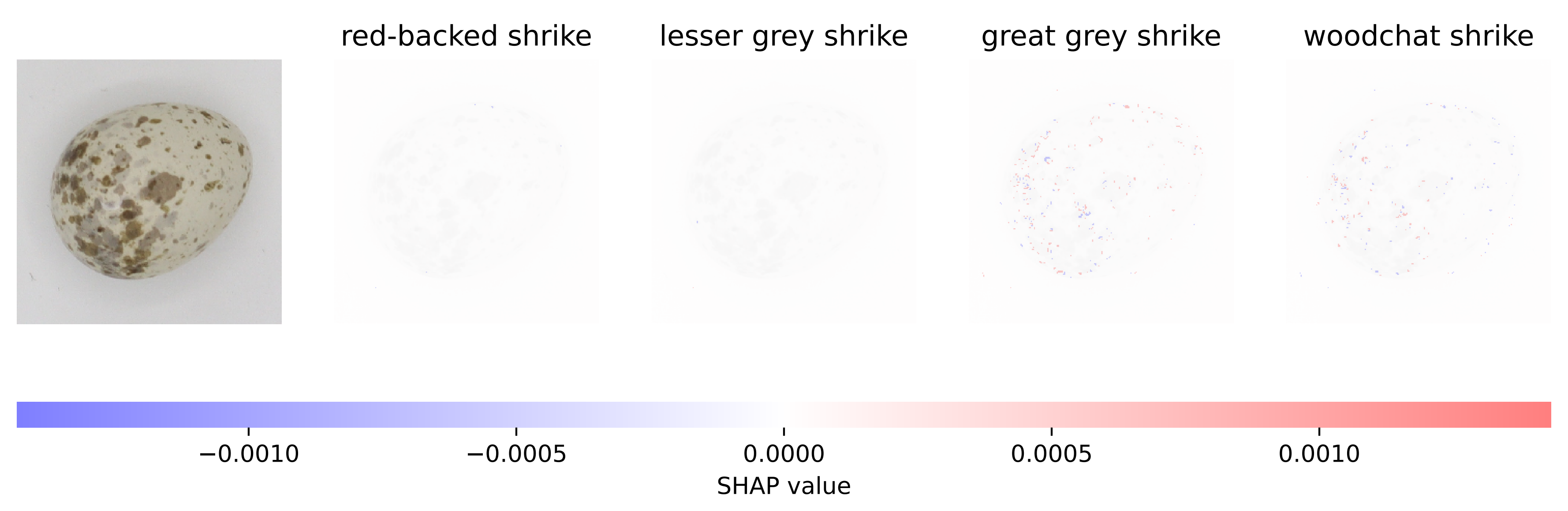

IMG_1318.JPG SHAP DeepExplainer major impact (Q3)

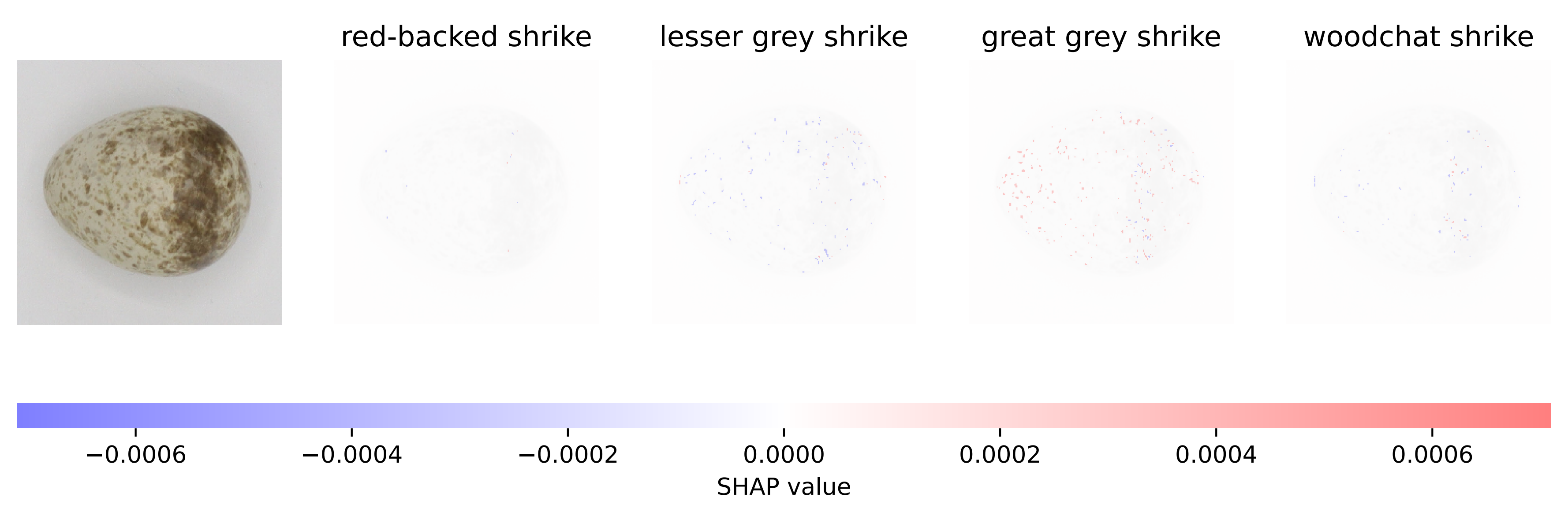

IMG_1417.JPG SHAP DeepExplainer major impact (Q3)

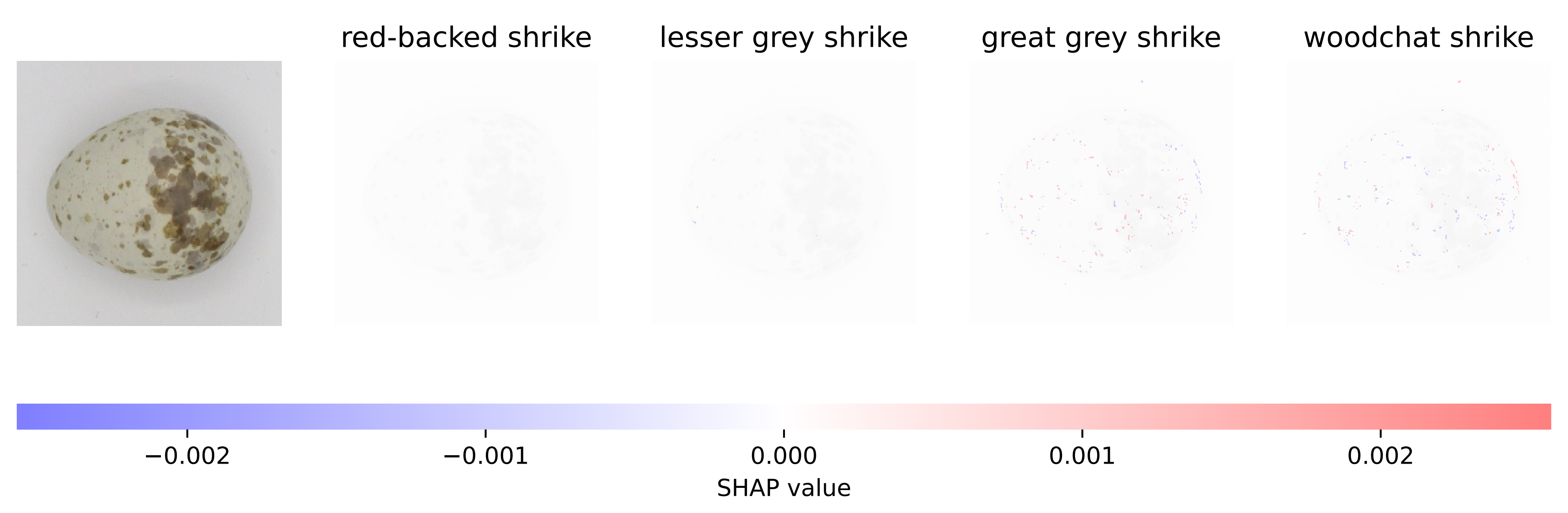

IMG_0267.JPG SHAP DeepExplainer major impact (Q3)

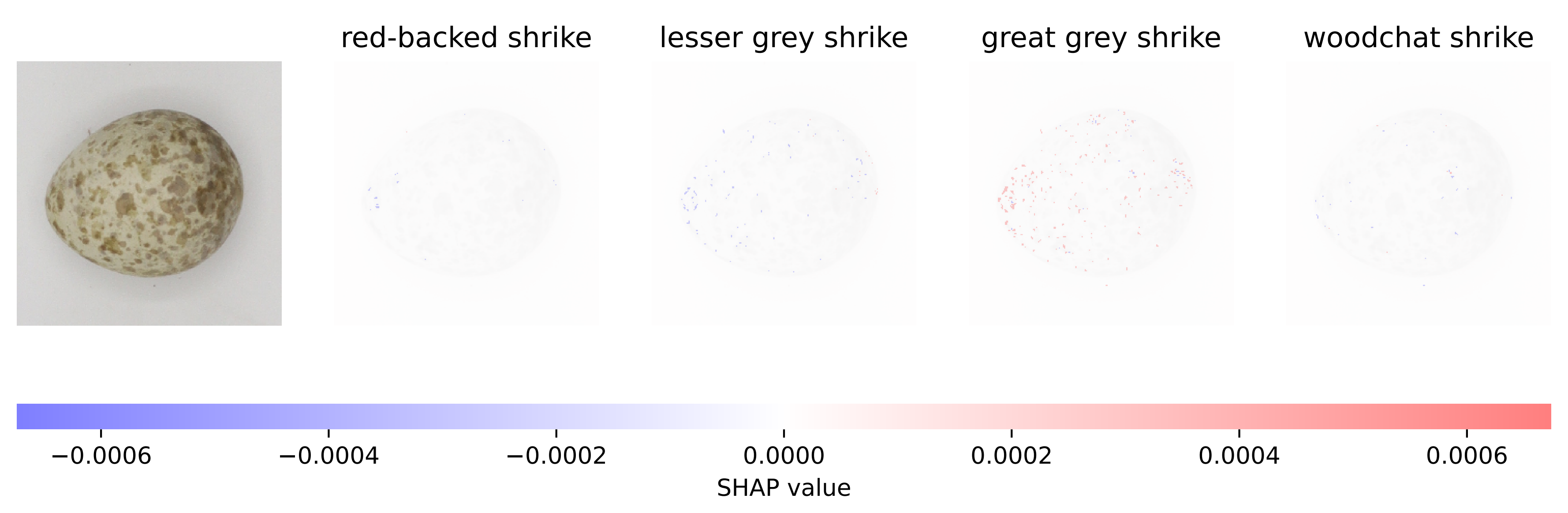

IMG_0273.JPG SHAP DeepExplainer major impact (Q3)

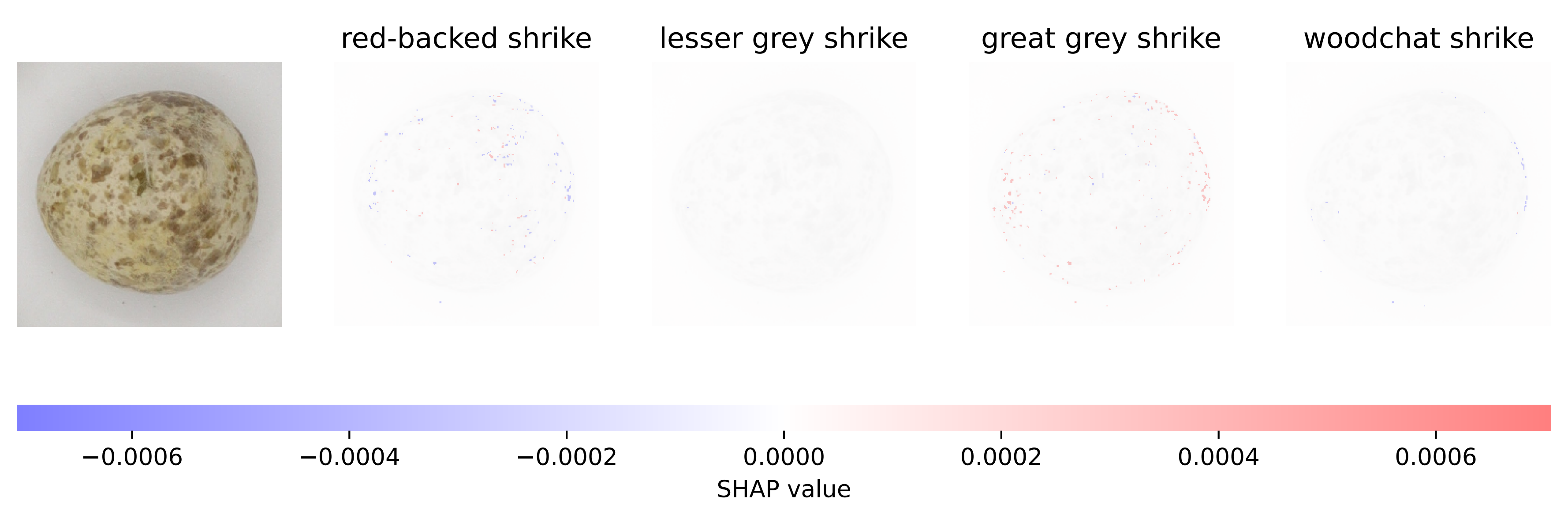

Supplement: S1 File — (ZIP) [file pone.0321532.s001.zip › S1-File-Class-predictions/shap - great grey shrike - mj.pdf]
