# Supplementary material for: Can explainable AI classify shrike (Laniidae) eggs by uncovering species-wide pigmentation patterns?
Source: PLoS One. 2025 May 2;20(5):e0321532. doi: 10.1371/journal.pone.0321532 (PMC12047758; doi:10.1371/journal.pone.0321532)

IMG_1595.JPG SHAP DeepExplainer major impact (Q3)

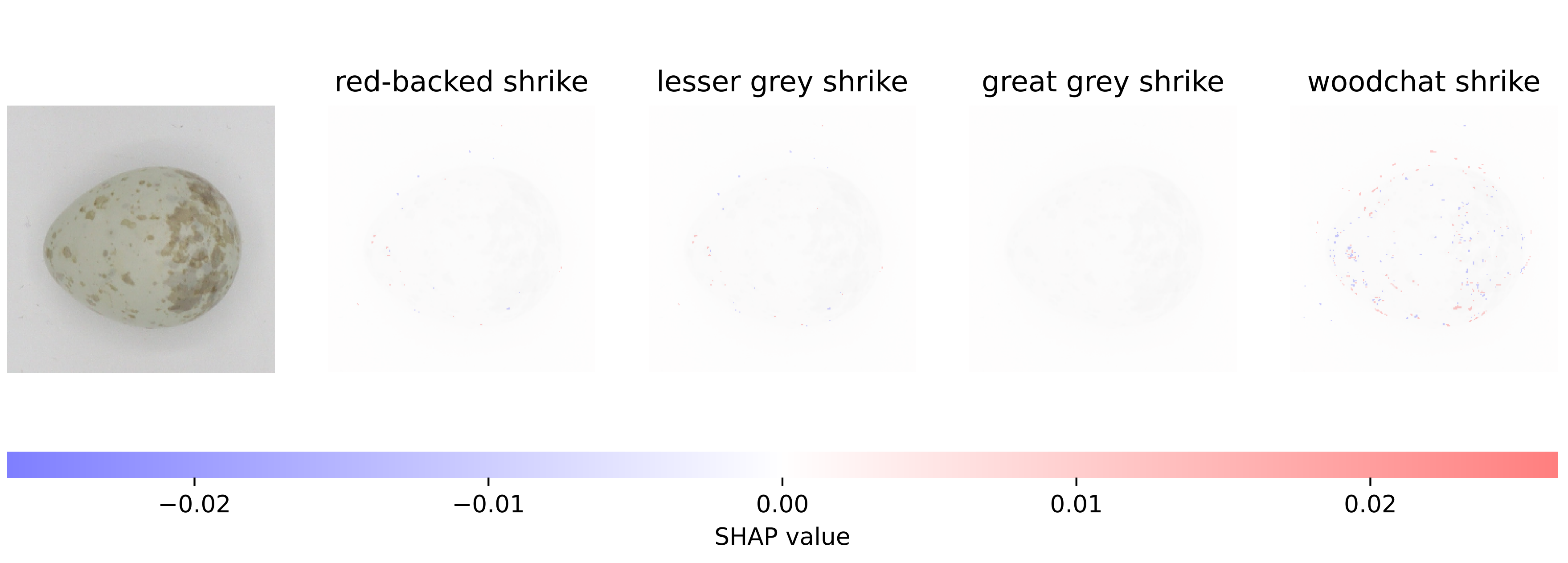

IMG_1666.JPG SHAP DeepExplainer major impact (Q3)

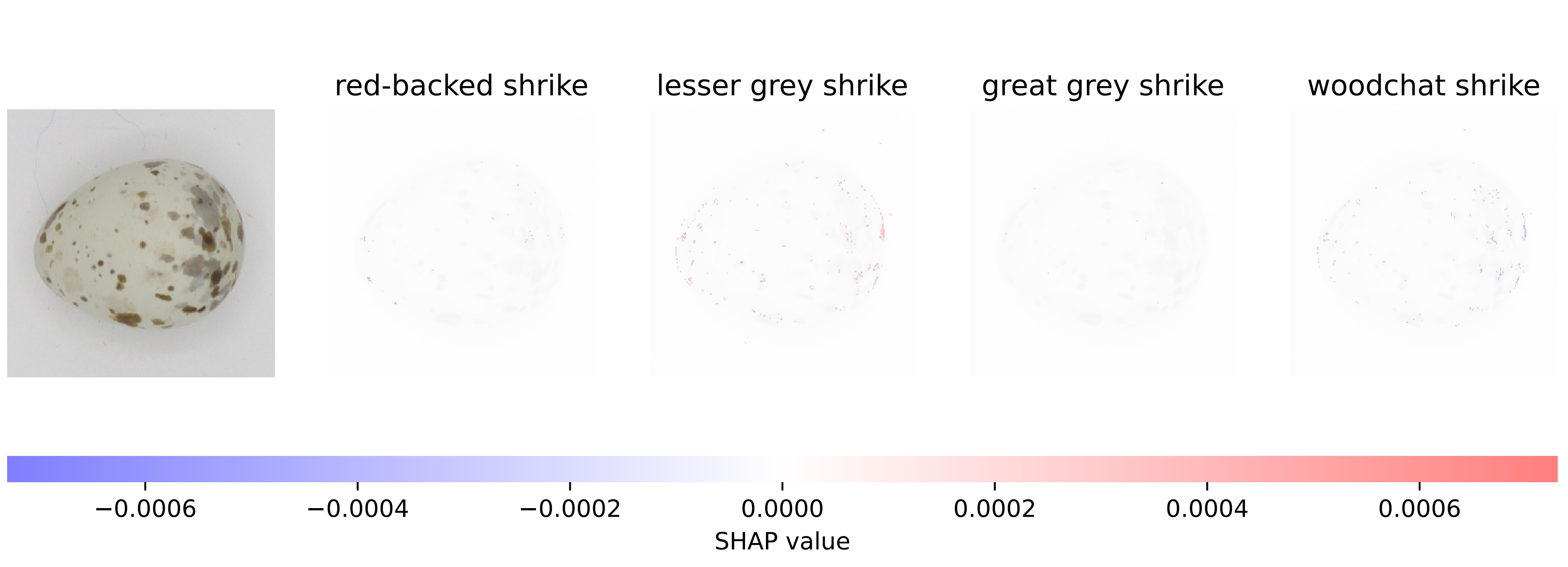

IMG_1675.JPG SHAP DeepExplainer major impact (Q3)

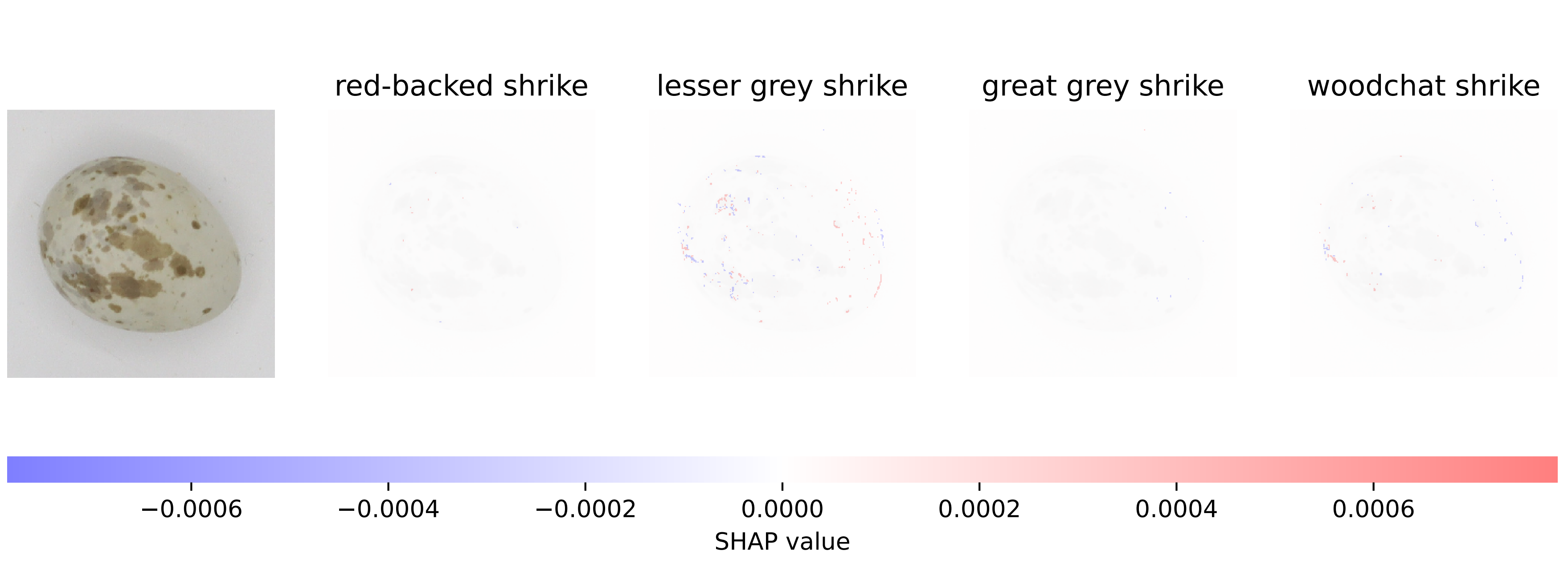

IMG_1678.JPG SHAP DeepExplainer major impact (Q3)

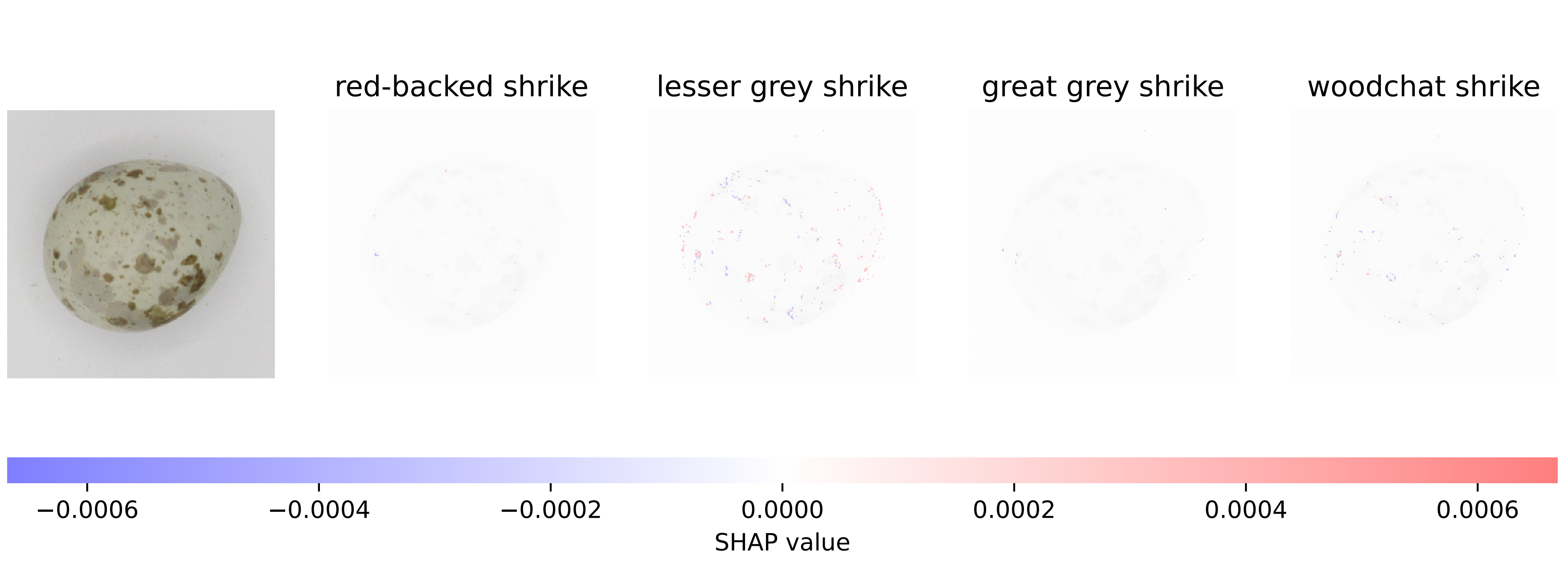

IMG_1723.JPG SHAP DeepExplainer major impact (Q3)

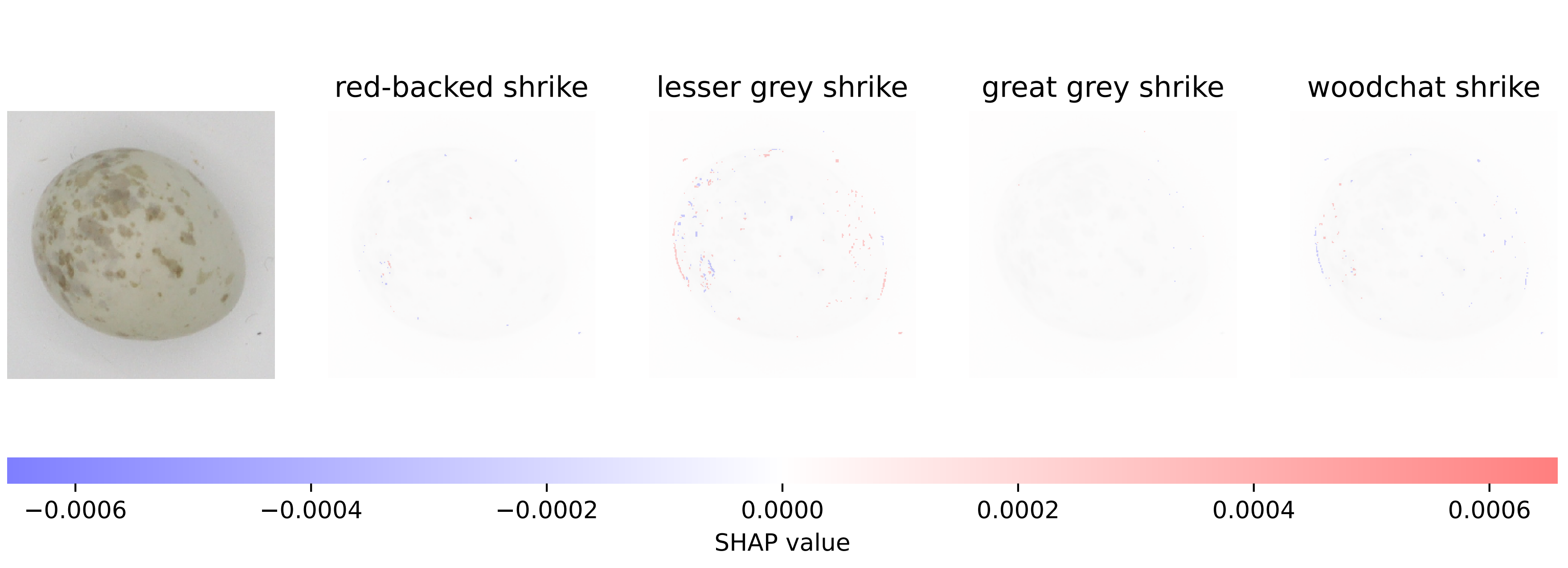

IMG_1749.JPG SHAP DeepExplainer major impact (Q3)

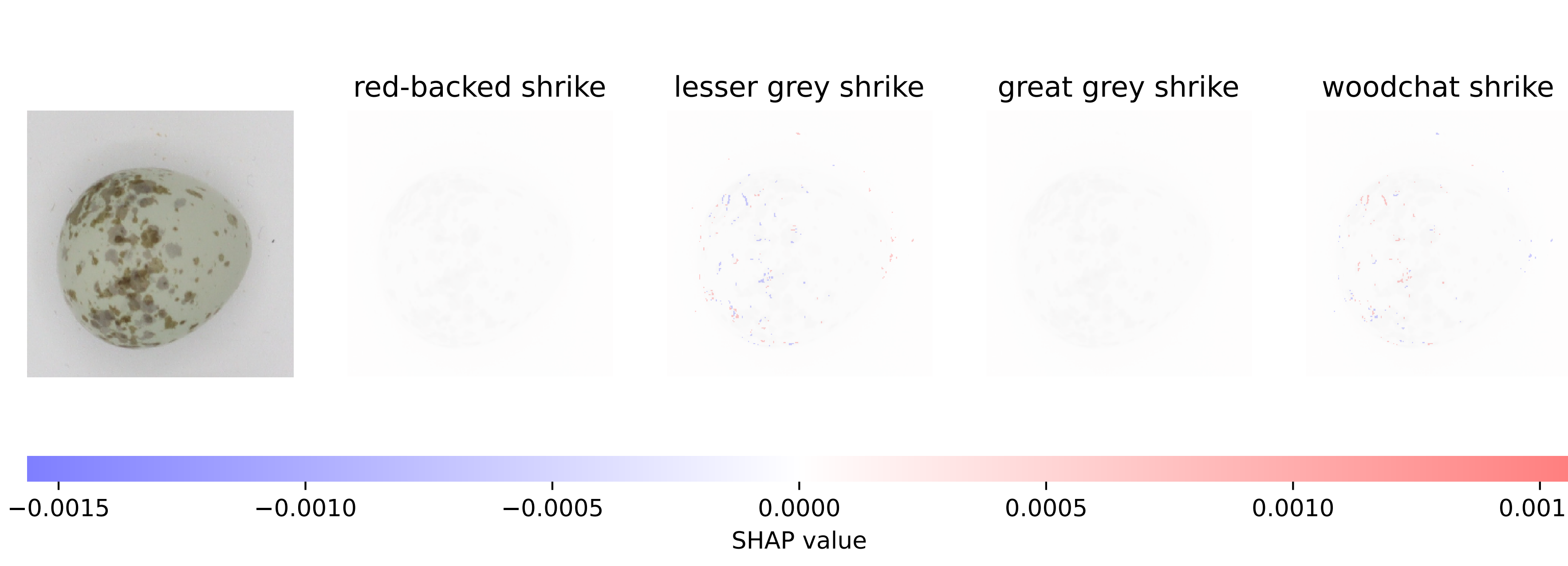

IMG_1783.JPG SHAP DeepExplainer major impact (Q3)

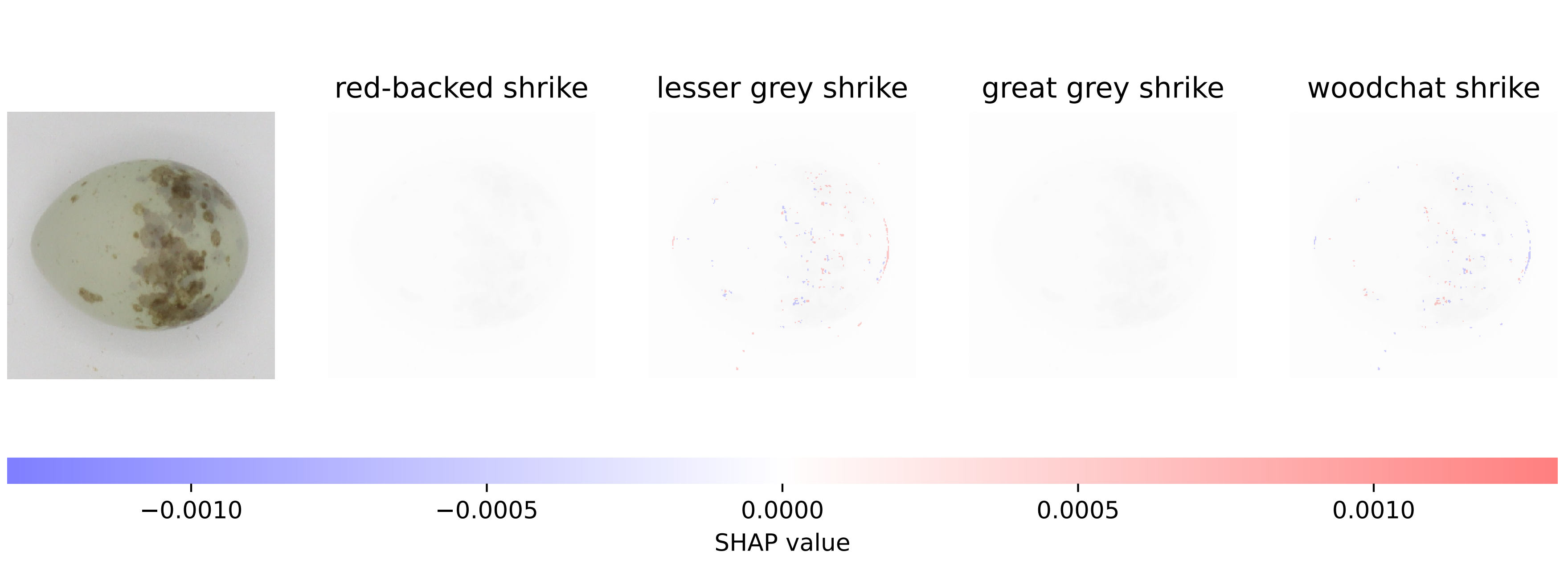

IMG_1786.JPG SHAP DeepExplainer major impact (Q3)

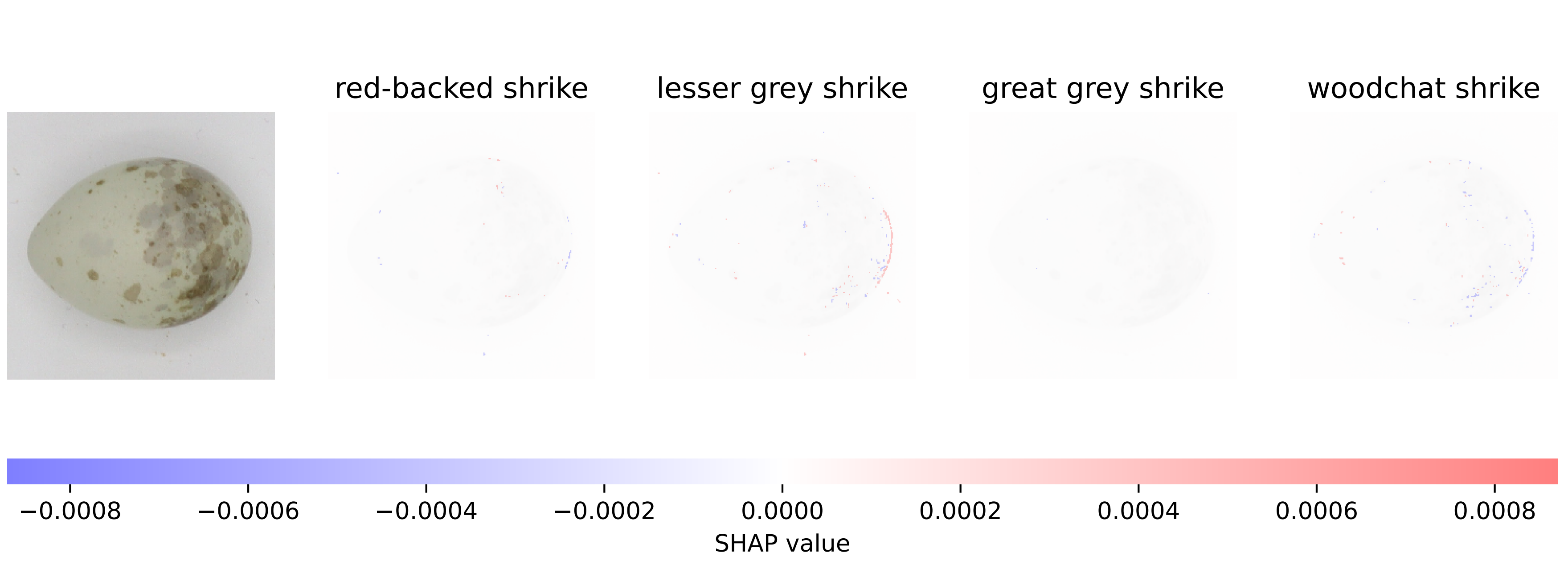

IMG_1845.JPG SHAP DeepExplainer major impact (Q3)

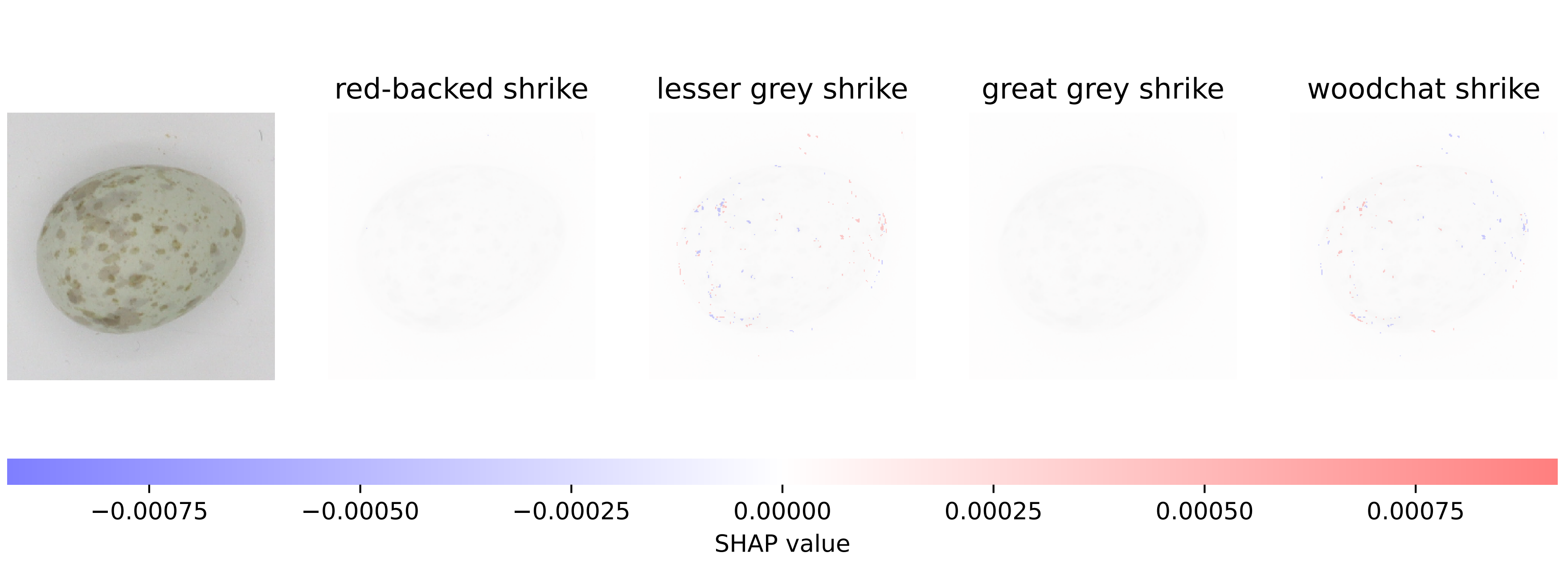

IMG_2663.JPG SHAP DeepExplainer major impact (Q3)

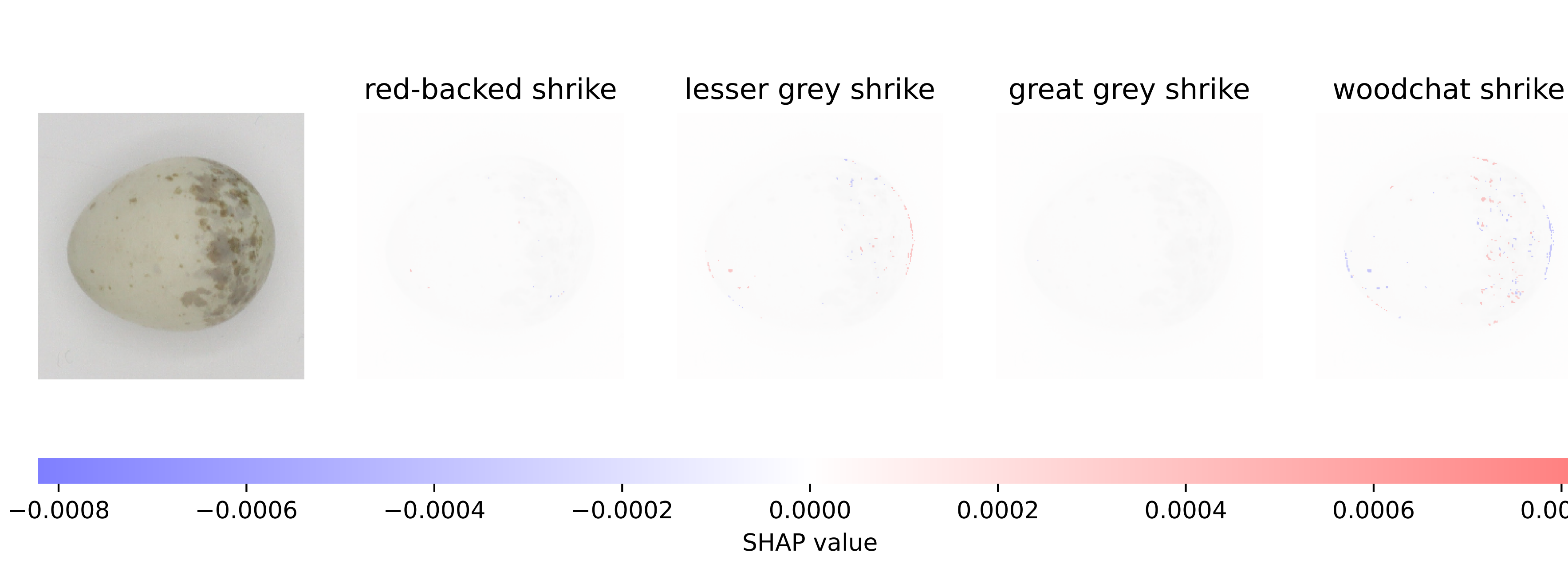

IMG_2668.JPG SHAP DeepExplainer major impact (Q3)

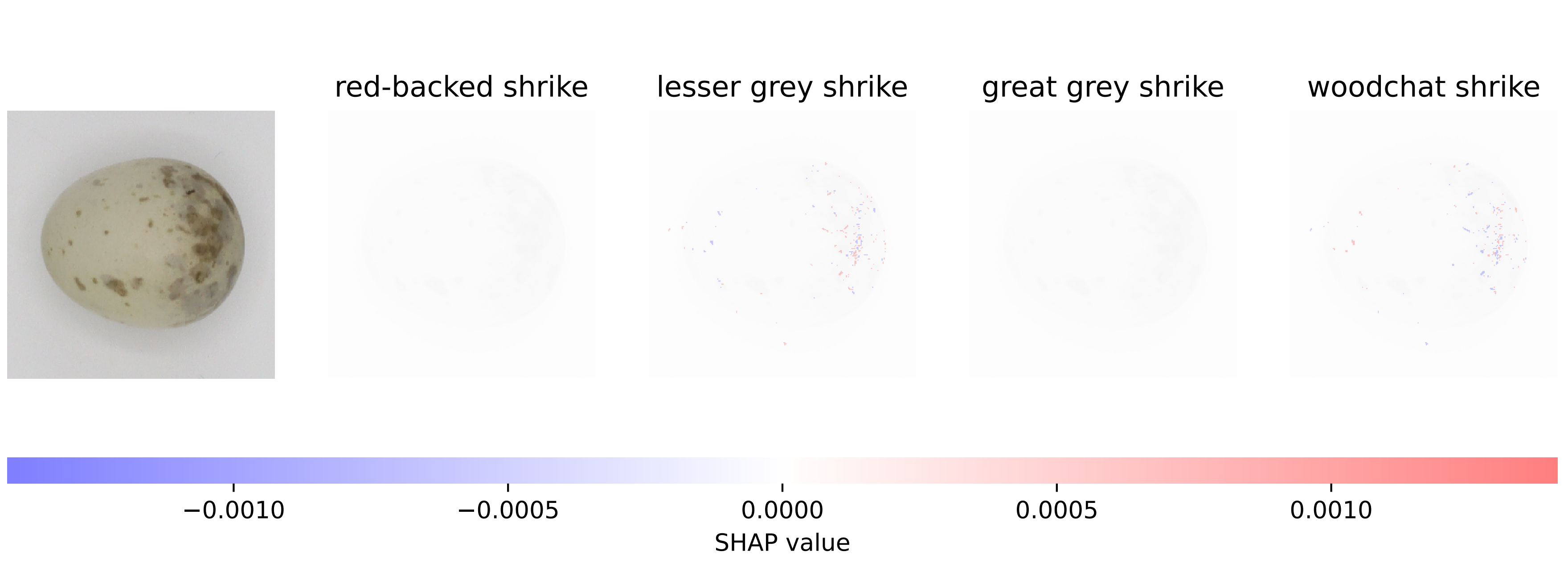

IMG_2008.JPG SHAP DeepExplainer major impact (Q3)

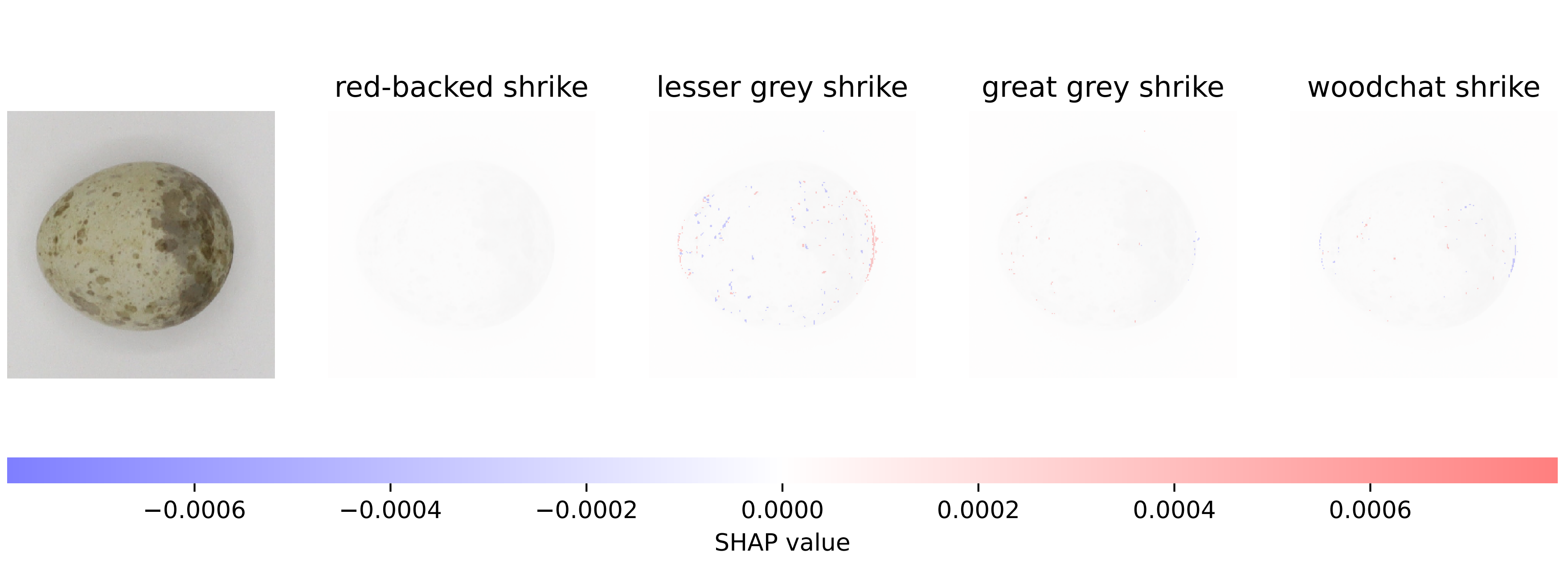

IMG_0319.JPG SHAP DeepExplainer major impact (Q3)

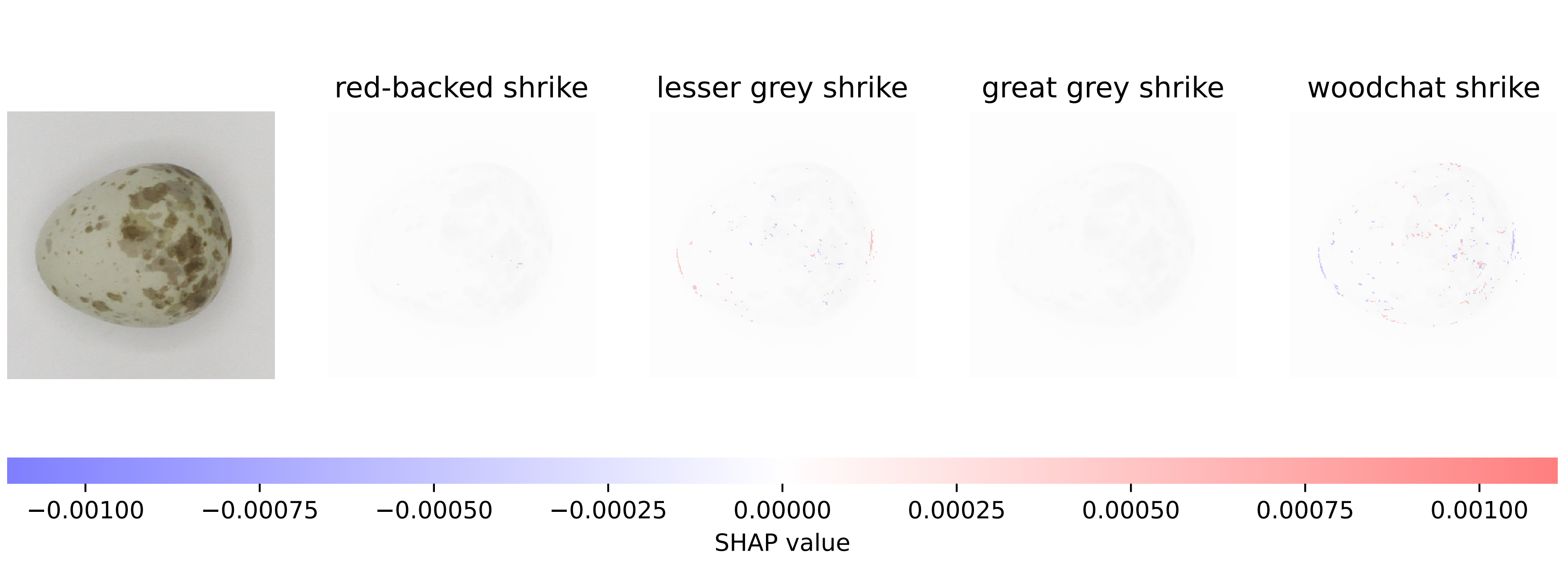

Supplement: S1 File — (ZIP) [file pone.0321532.s001.zip › S1-File-Class-predictions/shap - lesser grey shrike - mj.pdf]
