# Supplementary material for: Can explainable AI classify shrike (Laniidae) eggs by uncovering species-wide pigmentation patterns?
Source: PLoS One. 2025 May 2;20(5):e0321532. doi: 10.1371/journal.pone.0321532 (PMC12047758; doi:10.1371/journal.pone.0321532)
Supplement: S1 File — (ZIP) [file pone.0321532.s001.zip › S1-File-Class-predictions/shap - lesser grey shrike.pdf]

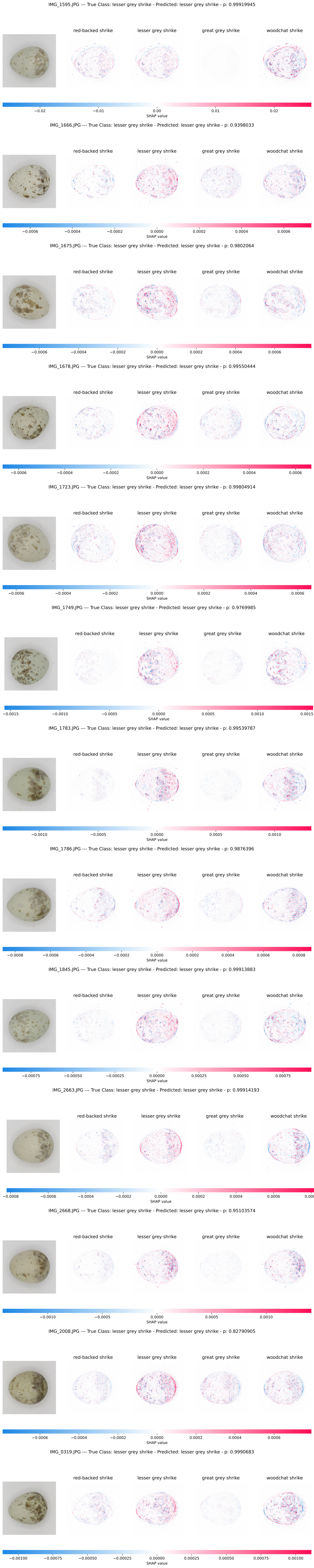
IMG_1595.JPG --- True Class: lesser grey shrike - Predicted: lesser grey shrike - p: 0.99919945
red-backed shrike
lesser grey shrike
great grey shrike
woodchat shrike
−0.02 −0.01 0.00 0.01 0.02
SHAP value
IMG_1666.JPG --- True Class: lesser grey shrike - Predicted: lesser grey shrike - p: 0.9398033
red-backed shrike
lesser grey shrike
great grey shrike
woodchat shrike
−0.0006 −0.0004 −0.0002 0.0000 0.0002 0.0004 0.0006
SHAP value
IMG_1675.JPG --- True Class: lesser grey shrike - Predicted: lesser grey shrike - p: 0.9802064
red-backed shrike
lesser grey shrike
great grey shrike
woodchat shrike
−0.0006 −0.0004 −0.0002 0.0000 0.0002 0.0004 0.0006
SHAP value
IMG_1678.JPG --- True Class: lesser grey shrike - Predicted: lesser grey shrike - p: 0.99550444
red-backed shrike
lesser grey shrike
great grey shrike
woodchat shrike
−0.0006 −0.0004 −0.0002 0.0000 0.0002 0.0004 0.0006
SHAP value
IMG_1723.JPG --- True Class: lesser grey shrike - Predicted: lesser grey shrike - p: 0.99804914
red-backed shrike
lesser grey shrike
great grey shrike
woodchat shrike
−0.0006 −0.0004 −0.0002 0.0000 0.0002 0.0004 0.0006
SHAP value
IMG_1749.JPG --- True Class: lesser grey shrike - Predicted: lesser grey shrike - p: 0.9769985
red-backed shrike
lesser grey shrike
great grey shrike
woodchat shrike
−0.0015 −0.0010 −0.0005 0.0000 0.0005 0.0010 0.0015
SHAP value
IMG_1783.JPG --- True Class: lesser grey shrike - Predicted: lesser grey shrike - p: 0.99539787
red-backed shrike
lesser grey shrike
great grey shrike
woodchat shrike
−0.0010 −0.0005 0.0000 0.0005 0.0010
SHAP value
IMG_1786.JPG --- True Class: lesser grey shrike - Predicted: lesser grey shrike - p: 0.9876396
red-backed shrike
lesser grey shrike
great grey shrike
woodchat shrike
−0.0008 −0.0006 −0.0004 −0.0002 0.0000 0.0002 0.0004 0.0006 0.0008
SHAP value
IMG_1845.JPG --- True Class: lesser grey shrike - Predicted: lesser grey shrike - p: 0.99913883
red-backed shrike
lesser grey shrike
great grey shrike
woodchat shrike
−0.00075 −0.00050 −0.00025 0.00000 0.00025 0.00050 0.00075
SHAP value
IMG_2663.JPG --- True Class: lesser grey shrike - Predicted: lesser grey shrike - p: 0.99914193
red-backed shrike
lesser grey shrike
great grey shrike
woodchat shrike
−0.0008 −0.0006 −0.0004 −0.0002 0.0000 0.0002 0.0004 0.0006 [illegible]
SHAP value
IMG_2668.JPG --- True Class: lesser grey shrike - Predicted: lesser grey shrike - p: 0.95103574
red-backed shrike
lesser grey shrike
great grey shrike
woodchat shrike
−0.0010 −0.0005 0.0000 0.0005 0.0010
SHAP value
IMG_2008.JPG --- True Class: lesser grey shrike - Predicted: lesser grey shrike - p: 0.82790905
red-backed shrike
lesser grey shrike
great grey shrike
woodchat shrike
−0.0006 −0.0004 −0.0002 0.0000 0.0002 0.0004 0.0006
SHAP value
IMG_0319.JPG --- True Class: lesser grey shrike - Predicted: lesser grey shrike - p: 0.9990683
red-backed shrike
lesser grey shrike
great grey shrike
woodchat shrike
−0.00100 −0.00075 −0.00050 −0.00025 0.00000 0.00025 0.00050 0.00075 0.00100
SHAP value
