# Supplementary material for: Can explainable AI classify shrike (Laniidae) eggs by uncovering species-wide pigmentation patterns?
Source: PLoS One. 2025 May 2;20(5):e0321532. doi: 10.1371/journal.pone.0321532 (PMC12047758; doi:10.1371/journal.pone.0321532)
Supplement: S1 File — (ZIP) [file pone.0321532.s001.zip › S1-File-Class-predictions/shap - red-backed shrike - mj.pdf]

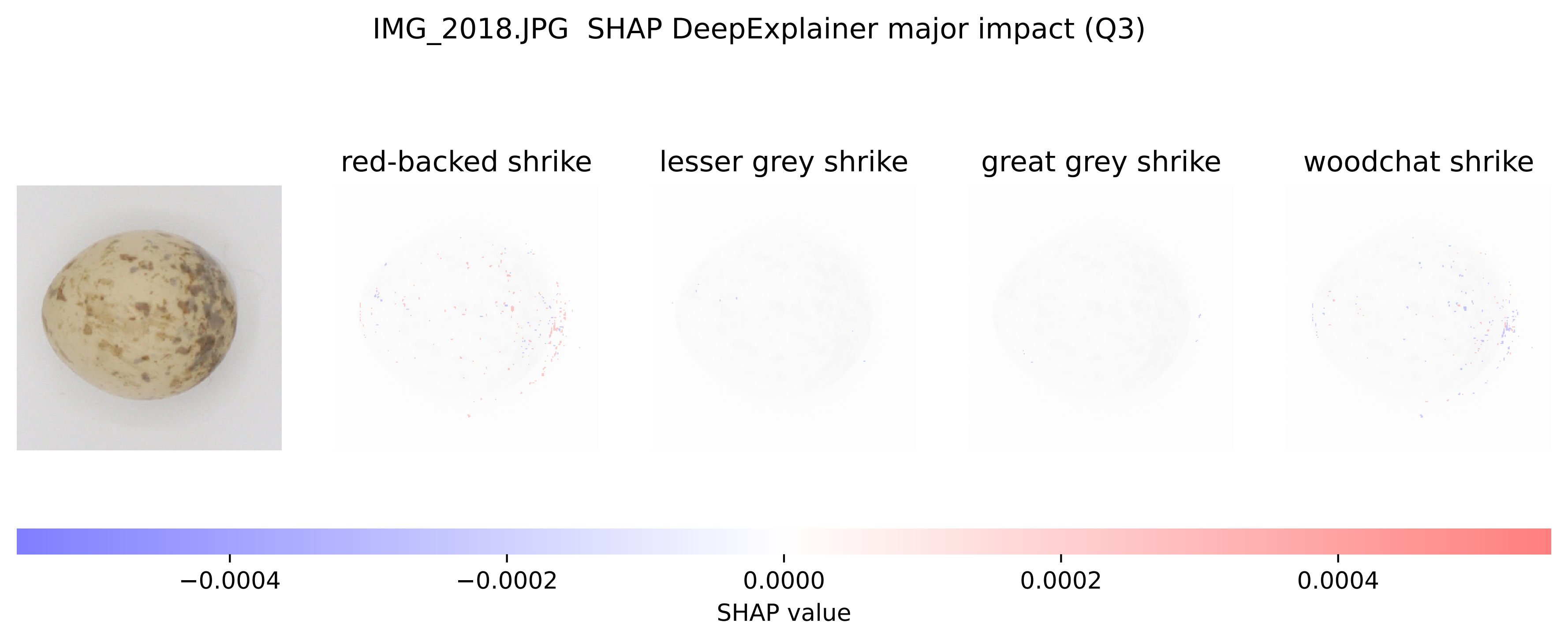
IMG_2018.JPG SHAP DeepExplainer major impact (Q3)
red-backed shrike
lesser grey shrike
great grey shrike
woodchat shrike
−0.0004
−0.0002
0.0000
0.0002
0.0004
SHAP value

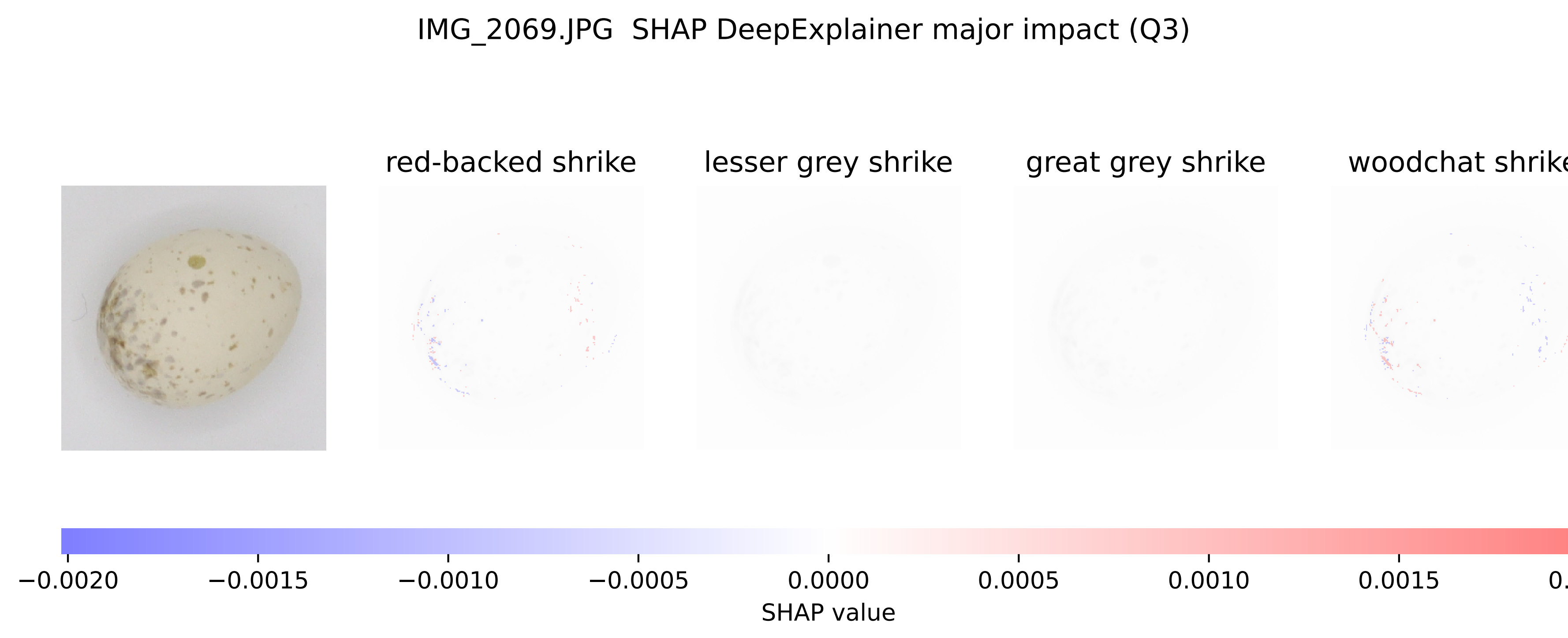
IMG_2069.JPG SHAP DeepExplainer major impact (Q3)
red-backed shrike
lesser grey shrike
great grey shrike
−0.0020
−0.0015
−0.0010
−0.0005
0.0000
0.0005
0.0010
0.0015
SHAP value

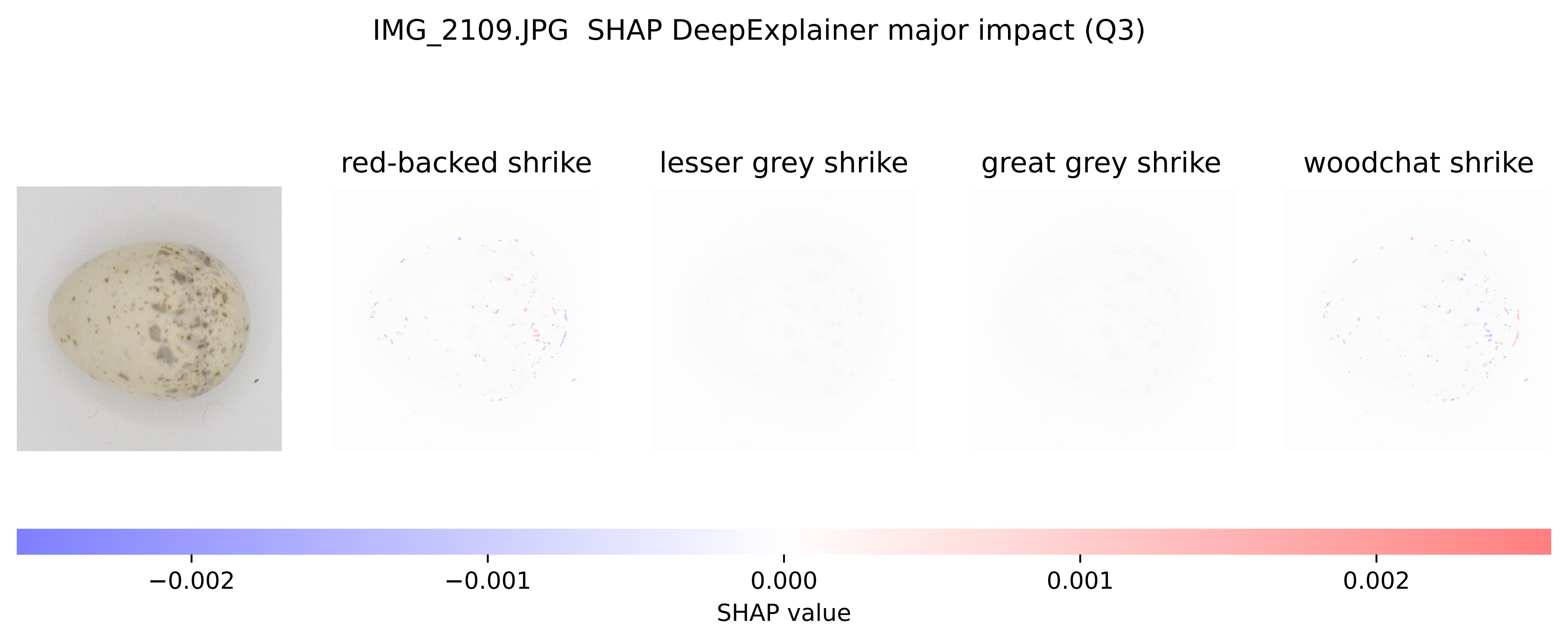
IMG_2109.JPG SHAP DeepExplainer major impact (Q3)
red-backed shrike
lesser grey shrike
great grey shrike
woodchat shrike
−0.002
−0.001
0.000
0.001
0.002
SHAP value

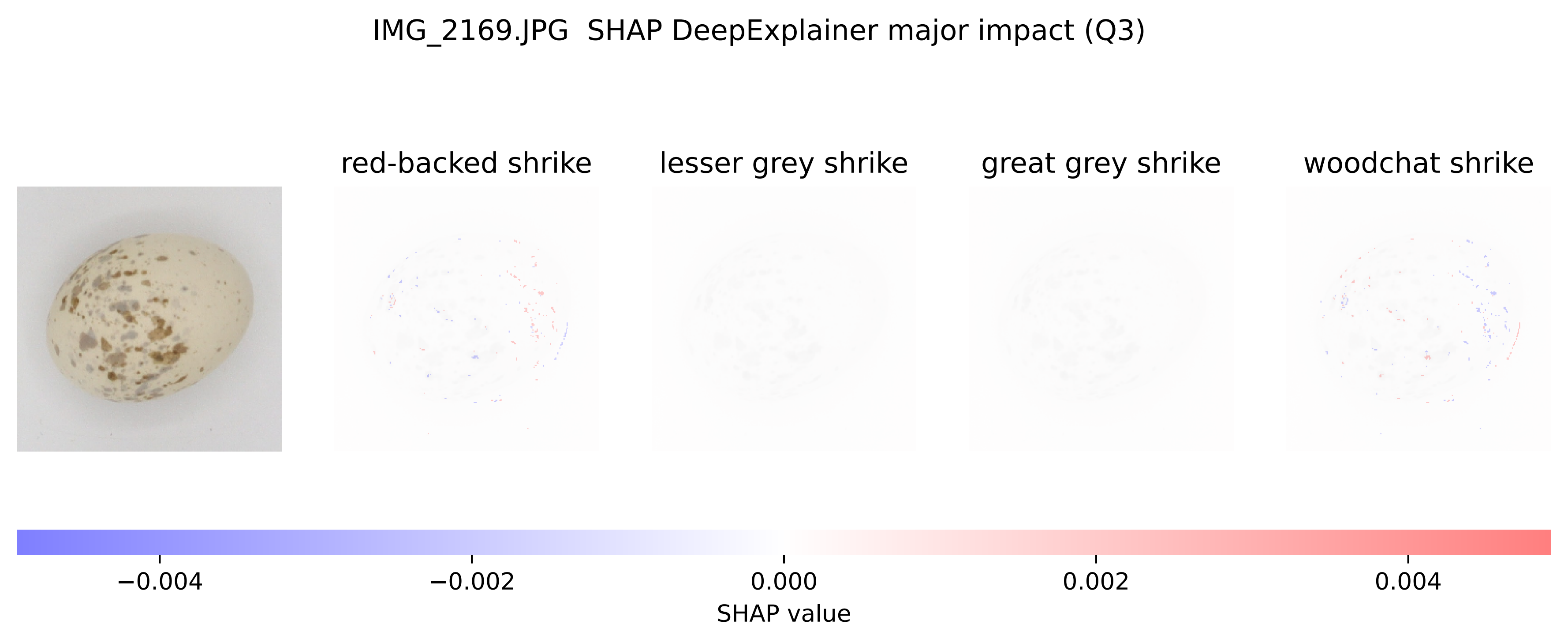
IMG_2169.JPG SHAP DeepExplainer major impact (Q3)
red-backed shrike
lesser grey shrike
great grey shrike
woodchat shrike
−0.004
−0.002
0.000
0.002
0.004
SHAP value

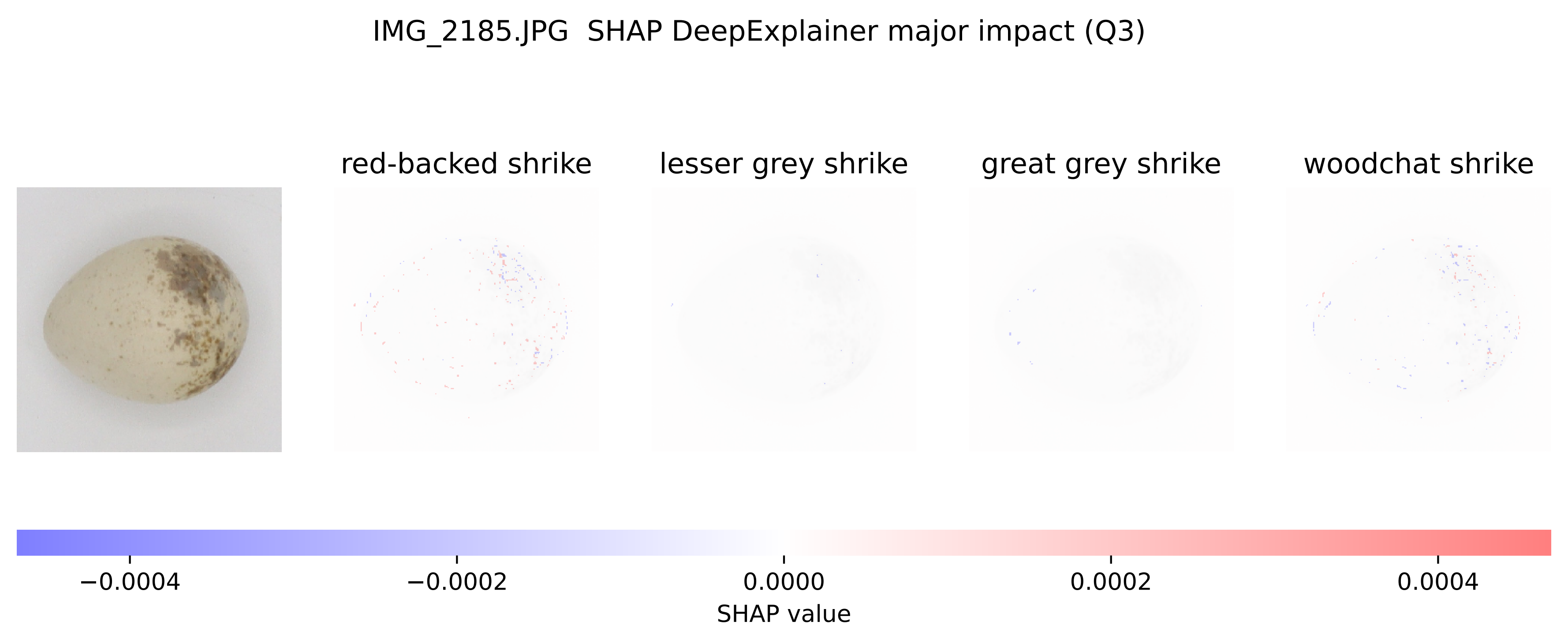
IMG_2185.JPG SHAP DeepExplainer major impact (Q3)
red-backed shrike
lesser grey shrike
great grey shrike
woodchat shrike
−0.0004
−0.0002
0.0000
0.0002
0.0004
SHAP value

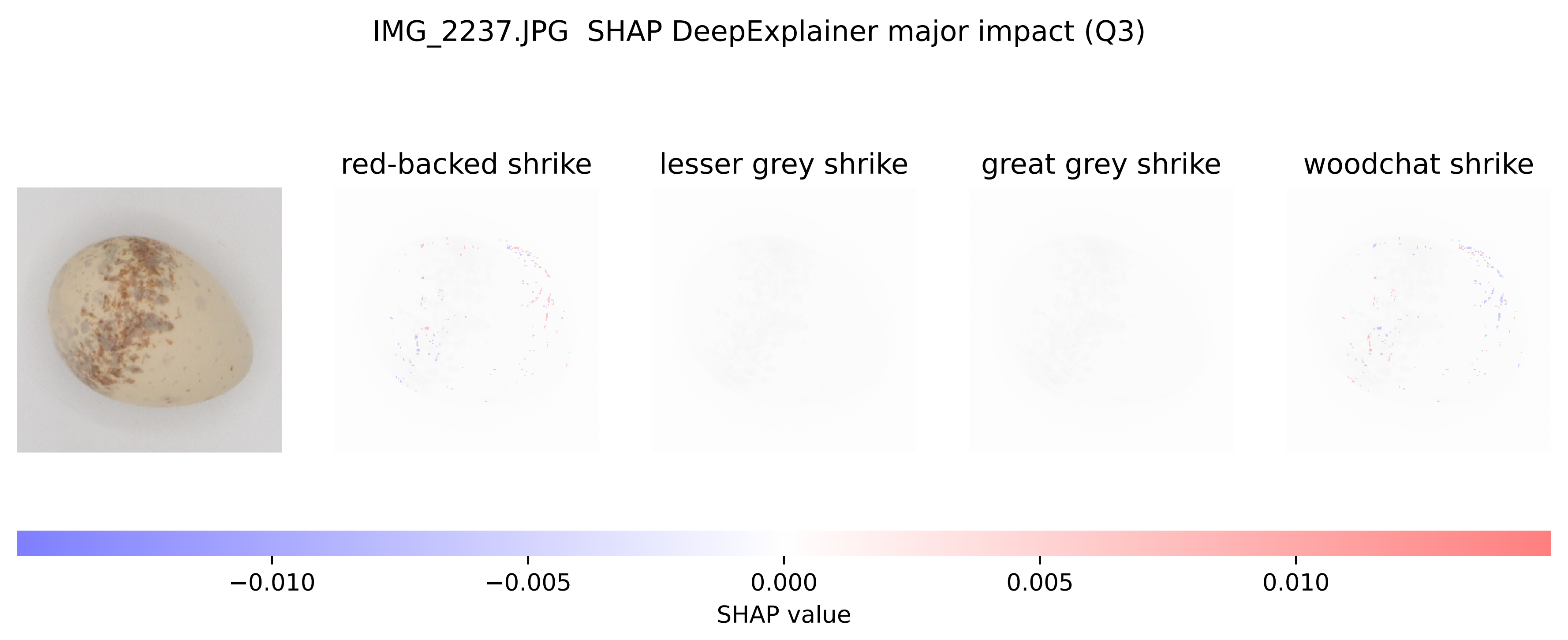
IMG_2237.JPG SHAP DeepExplainer major impact (Q3)
red-backed shrike
lesser grey shrike
great grey shrike
woodchat shrike
−0.010
−0.005
0.000
0.005
0.010
SHAP value

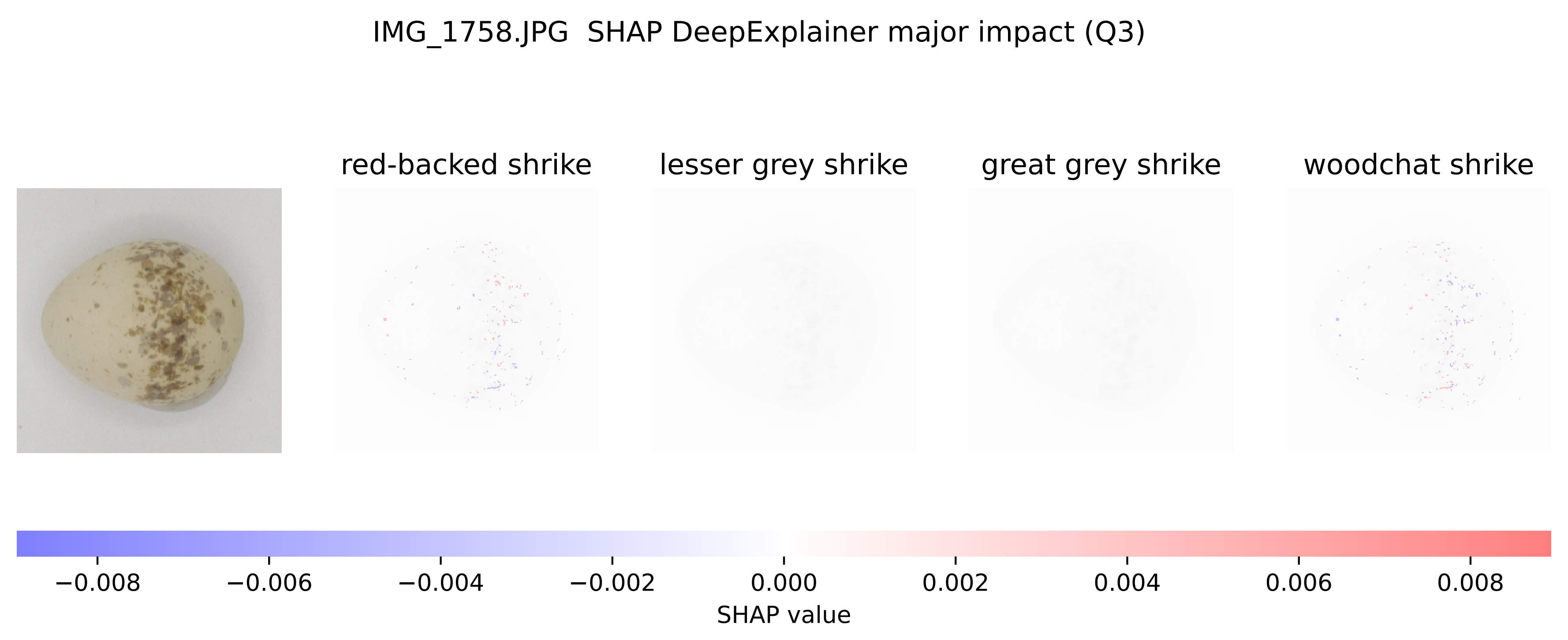
IMG_1758.JPG SHAP DeepExplainer major impact (Q3)
red-backed shrike
lesser grey shrike
great grey shrike
woodchat shrike
−0.008
−0.006
−0.004
−0.002
0.000
0.002
0.004
0.006
0.008
SHAP value

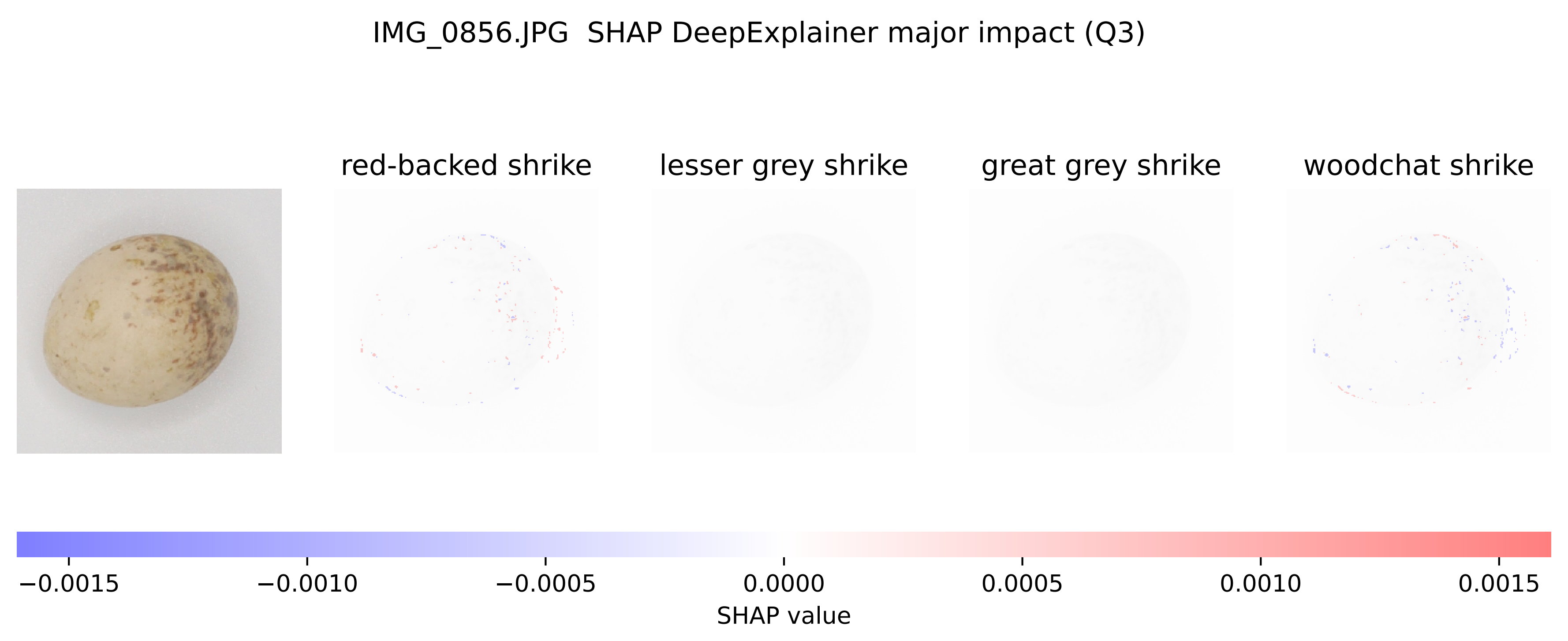
IMG_0856.JPG SHAP DeepExplainer major impact (Q3)
red-backed shrike
lesser grey shrike
great grey shrike
woodchat shrike
−0.0015
−0.0010
−0.0005
0.0000
0.0005
0.0010
0.0015
SHAP value

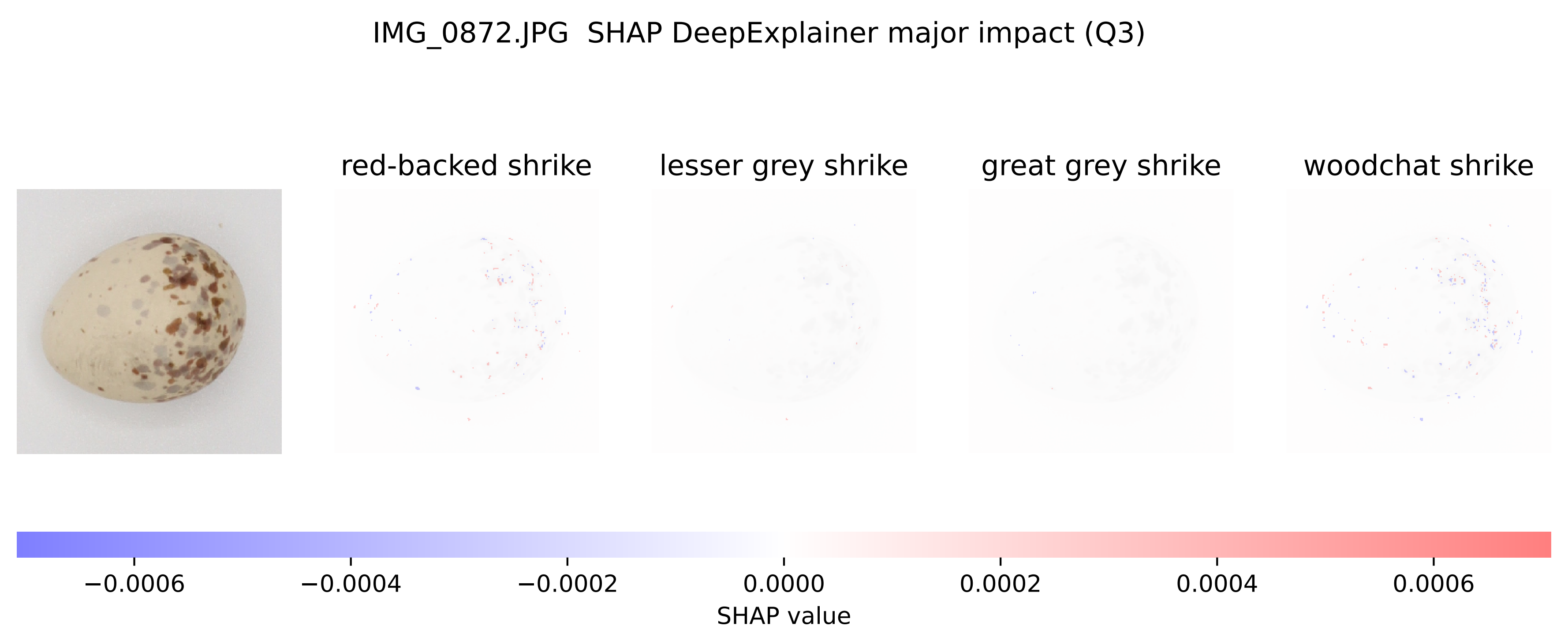
IMG_0872.JPG SHAP DeepExplainer major impact (Q3)
red-backed shrike
lesser grey shrike
great grey shrike
woodchat shrike
−0.0006
−0.0004
−0.0002
0.0000
0.0002
0.0004
0.0006
SHAP value

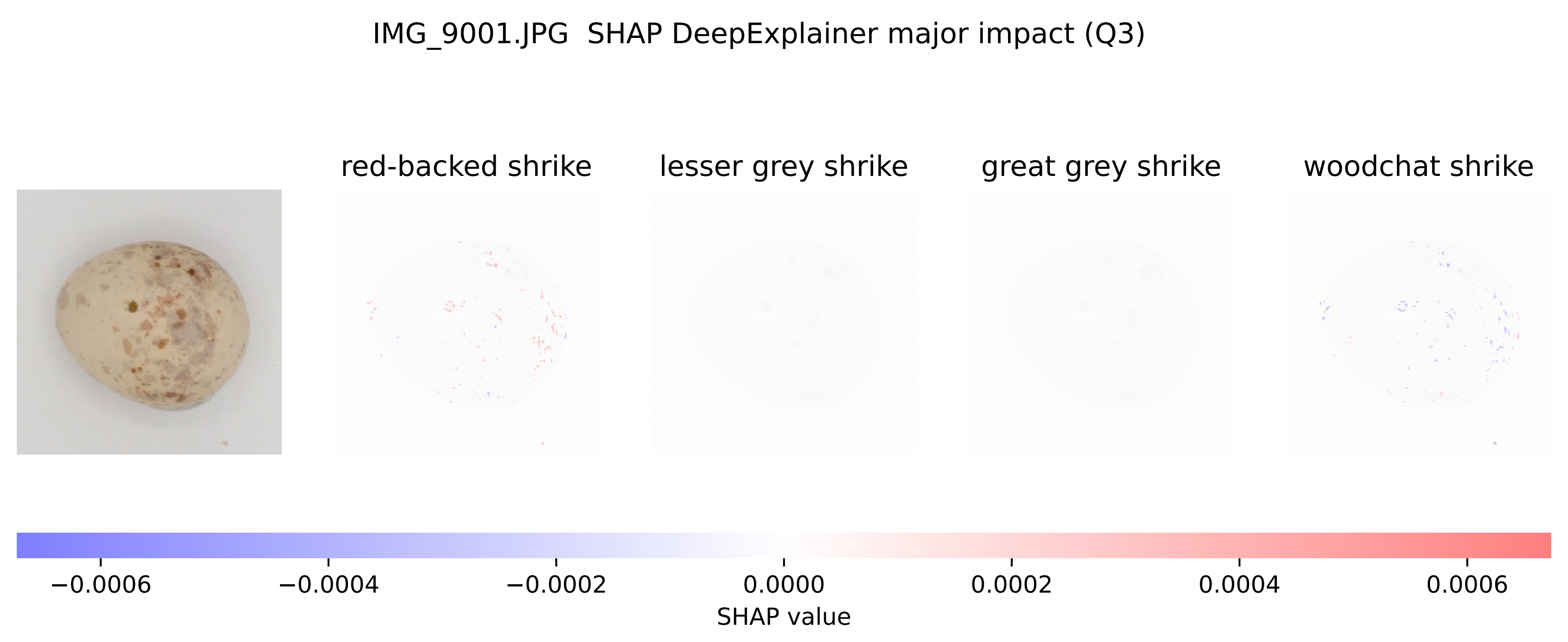
IMG_9001.JPG SHAP DeepExplainer major impact (Q3)
red-backed shrike
lesser grey shrike
great grey shrike
woodchat shrike
−0.0006
−0.0004
−0.0002
0.0000
0.0002
0.0004
0.0006
SHAP value

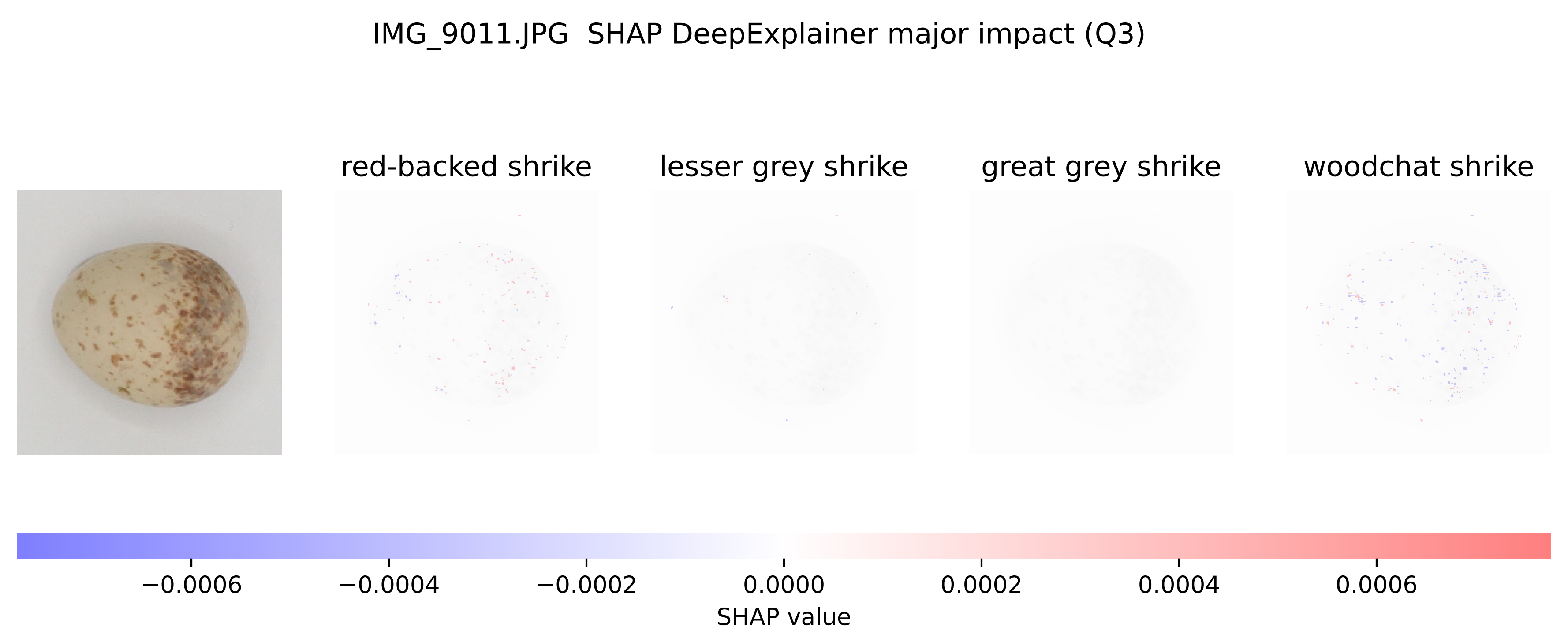
IMG_9011.JPG SHAP DeepExplainer major impact (Q3)
red-backed shrike
lesser grey shrike
great grey shrike
woodchat shrike
−0.0006
−0.0004
−0.0002
0.0000
0.0002
0.0004
0.0006
SHAP value

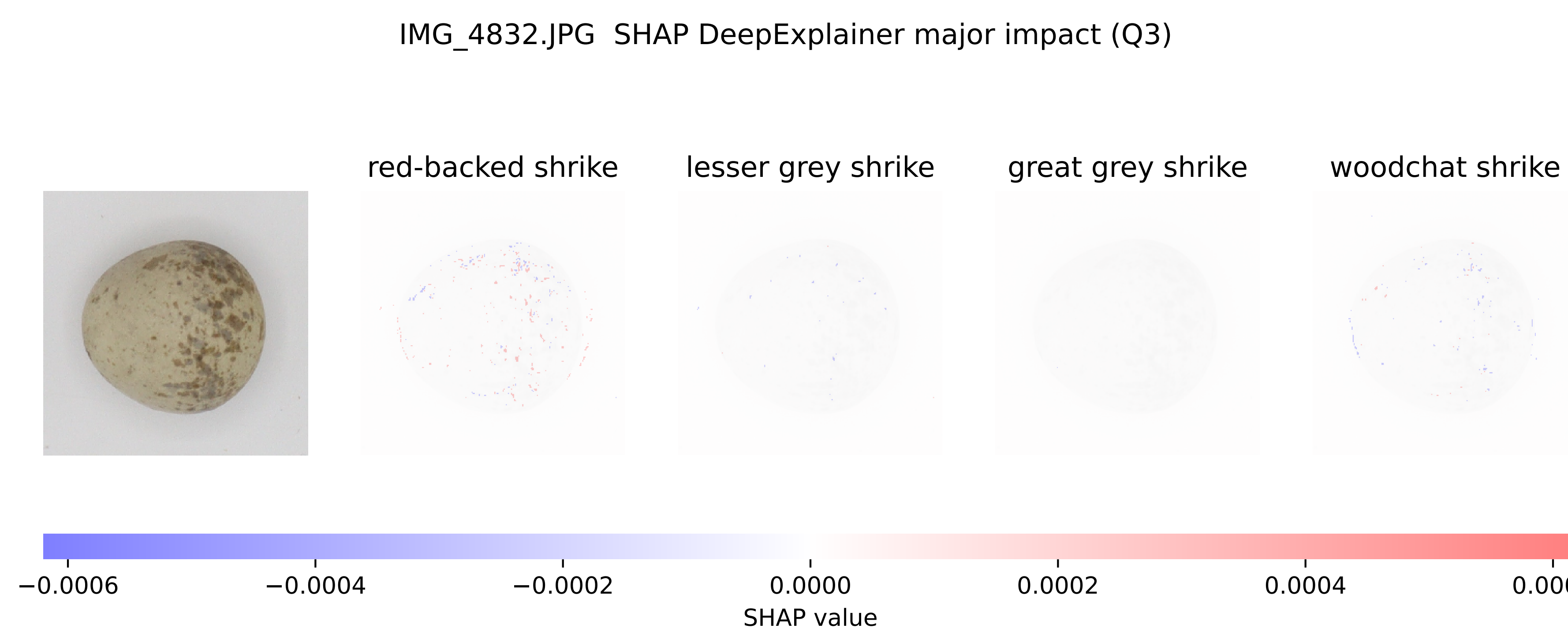
IMG_4832.JPG SHAP DeepExplainer major impact (Q3)
red-backed shrike
lesser grey shrike
great grey shrike
woodchat shrike
−0.0006
−0.0004
−0.0002
0.0000
0.0002
0.0004
SHAP value

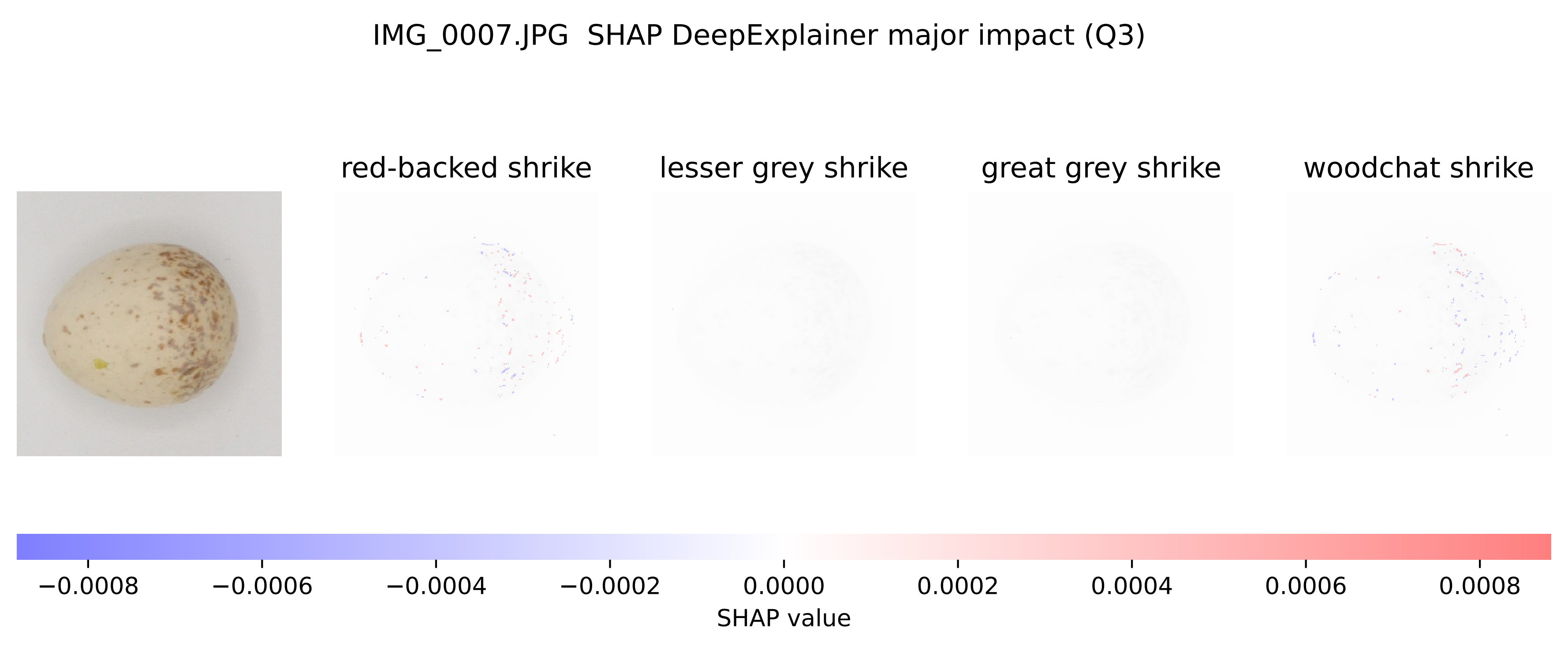
IMG_0007.JPG SHAP DeepExplainer major impact (Q3)
red-backed shrike
lesser grey shrike
great grey shrike
woodchat shrike
−0.0008
−0.0006
−0.0004
−0.0002
0.0000
0.0002
0.0004
0.0006
0.0008
SHAP value
