# Supplementary material for: Can explainable AI classify shrike (Laniidae) eggs by uncovering species-wide pigmentation patterns?
Source: PLoS One. 2025 May 2;20(5):e0321532. doi: 10.1371/journal.pone.0321532 (PMC12047758; doi:10.1371/journal.pone.0321532)
Supplement: S1 File — (ZIP) [file pone.0321532.s001.zip › S1-File-Class-predictions/shap - red-backed shrike.pdf]

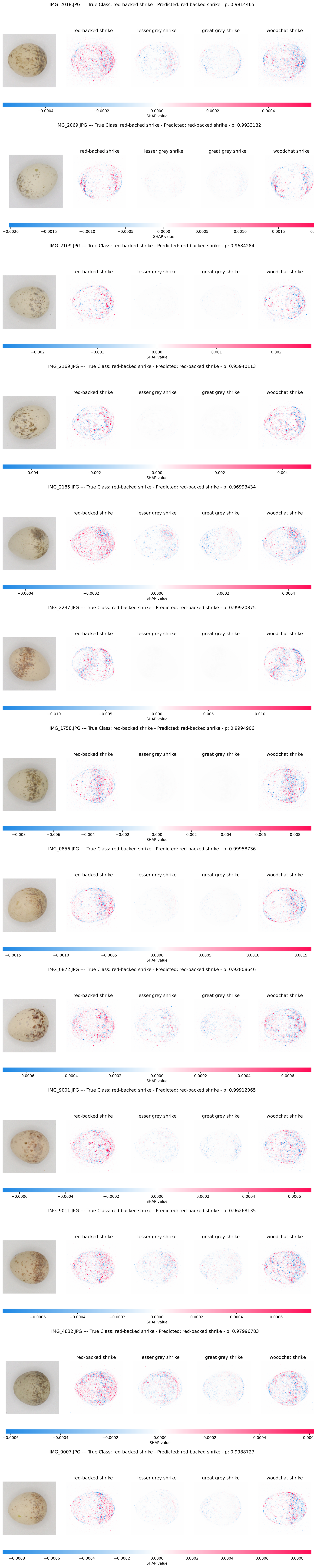
IMG_2018.JPG --- True Class: red-backed shrike - Predicted: red-backed shrike - p: 0.9814465
red-backed shrike
lesser grey shrike
great grey shrike
woodchat shrike
−0.0004 −0.0002 0.0000 0.0002 0.0004
SHAP value
IMG_2069.JPG --- True Class: red-backed shrike - Predicted: red-backed shrike - p: 0.9933182
red-backed shrike
lesser grey shrike
great grey shrike
woodchat shrike
−0.0020 −0.0015 −0.0010 −0.0005 0.0000 0.0005 0.0010 0.0015 0.
SHAP value
IMG_2109.JPG --- True Class: red-backed shrike - Predicted: red-backed shrike - p: 0.9684284
red-backed shrike
lesser grey shrike
great grey shrike
woodchat shrike
−0.002 −0.001 0.000 0.001 0.002
SHAP value
IMG_2169.JPG --- True Class: red-backed shrike - Predicted: red-backed shrike - p: 0.95940113
red-backed shrike
lesser grey shrike
great grey shrike
woodchat shrike
−0.004 −0.002 0.000 0.002 0.004
SHAP value
IMG_2185.JPG --- True Class: red-backed shrike - Predicted: red-backed shrike - p: 0.96993434
red-backed shrike
lesser grey shrike
great grey shrike
woodchat shrike
−0.0004 −0.0002 0.0000 0.0002 0.0004
SHAP value
IMG_2237.JPG --- True Class: red-backed shrike - Predicted: red-backed shrike - p: 0.99920875
red-backed shrike
lesser grey shrike
great grey shrike
woodchat shrike
−0.010 −0.005 0.000 0.005 0.010
SHAP value
IMG_1758.JPG --- True Class: red-backed shrike - Predicted: red-backed shrike - p: 0.9994906
red-backed shrike
lesser grey shrike
great grey shrike
woodchat shrike
−0.008 −0.006 −0.004 −0.002 0.000 0.002 0.004 0.006 0.008
SHAP value
IMG_0856.JPG --- True Class: red-backed shrike - Predicted: red-backed shrike - p: 0.99958736
red-backed shrike
lesser grey shrike
great grey shrike
woodchat shrike
−0.0015 −0.0010 −0.0005 0.0000 0.0005 0.0010 0.0015
SHAP value
IMG_0872.JPG --- True Class: red-backed shrike - Predicted: red-backed shrike - p: 0.92808646
red-backed shrike
lesser grey shrike
great grey shrike
woodchat shrike
−0.0006 −0.0004 −0.0002 0.0000 0.0002 0.0004 0.0006
SHAP value
IMG_9001.JPG --- True Class: red-backed shrike - Predicted: red-backed shrike - p: 0.99912065
red-backed shrike
lesser grey shrike
great grey shrike
woodchat shrike
−0.0006 −0.0004 −0.0002 0.0000 0.0002 0.0004 0.0006
SHAP value
IMG_9011.JPG --- True Class: red-backed shrike - Predicted: red-backed shrike - p: 0.96268135
red-backed shrike
lesser grey shrike
great grey shrike
woodchat shrike
−0.0006 −0.0004 −0.0002 0.0000 0.0002 0.0004 0.0006
SHAP value
IMG_4832.JPG --- True Class: red-backed shrike - Predicted: red-backed shrike - p: 0.97996783
red-backed shrike
lesser grey shrike
great grey shrike
woodchat shrike
−0.0006 −0.0004 −0.0002 0.0000 0.0002 0.0004 0.000
SHAP value
IMG_0007.JPG --- True Class: red-backed shrike - Predicted: red-backed shrike - p: 0.9988727
red-backed shrike
lesser grey shrike
great grey shrike
woodchat shrike
−0.0008 −0.0006 −0.0004 −0.0002 0.0000 0.0002 0.0004 0.0006 0.0008
SHAP value
