# Supplementary material for: Can explainable AI classify shrike (Laniidae) eggs by uncovering species-wide pigmentation patterns?
Source: PLoS One. 2025 May 2;20(5):e0321532. doi: 10.1371/journal.pone.0321532 (PMC12047758; doi:10.1371/journal.pone.0321532)

IMG_0316.JPG SHAP DeepExplainer major impact (Q3)

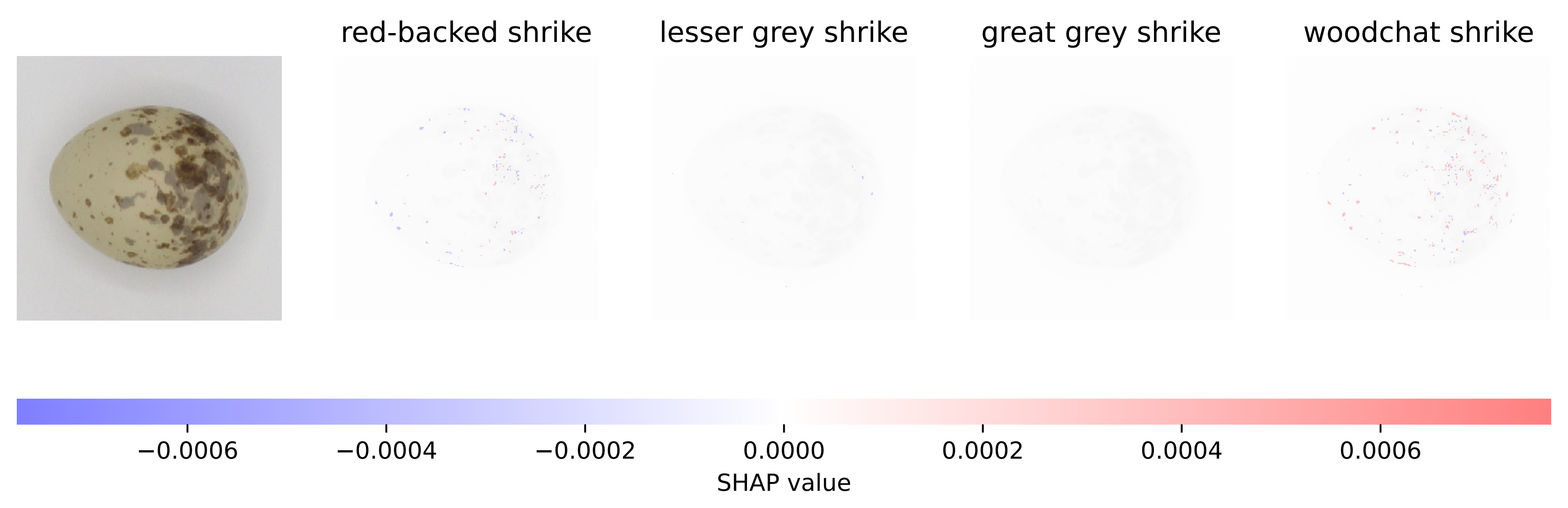

IMG_0320.JPG SHAP DeepExplainer major impact (Q3)

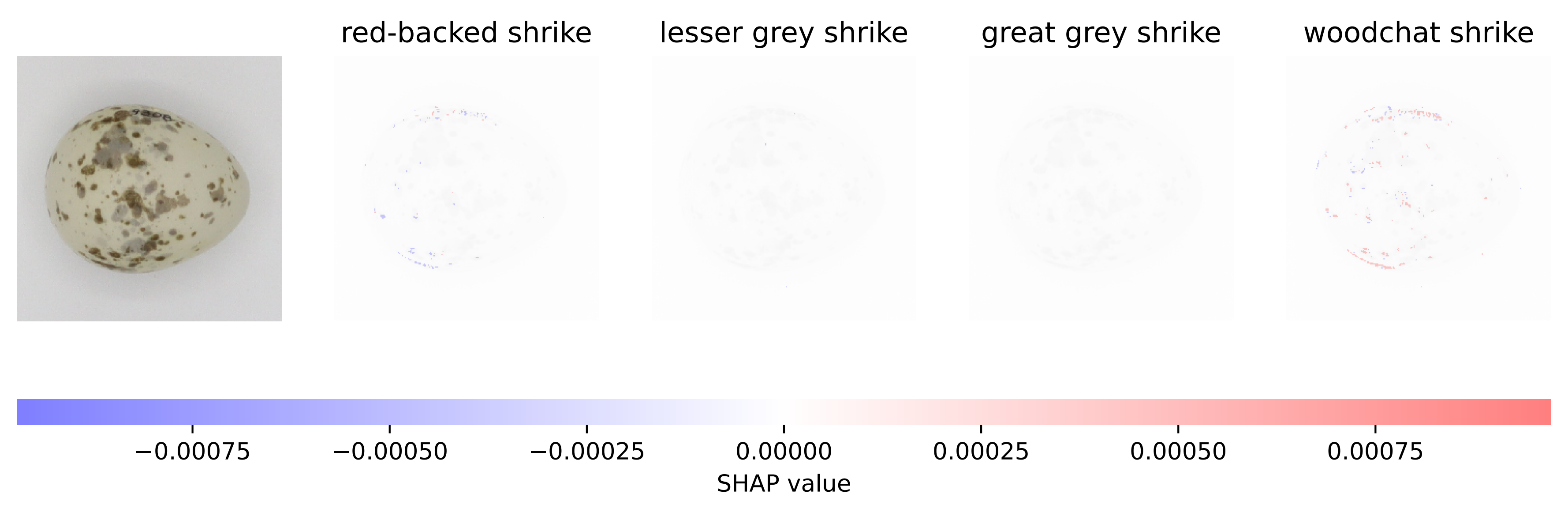

IMG_0324.JPG SHAP DeepExplainer major impact (Q3)

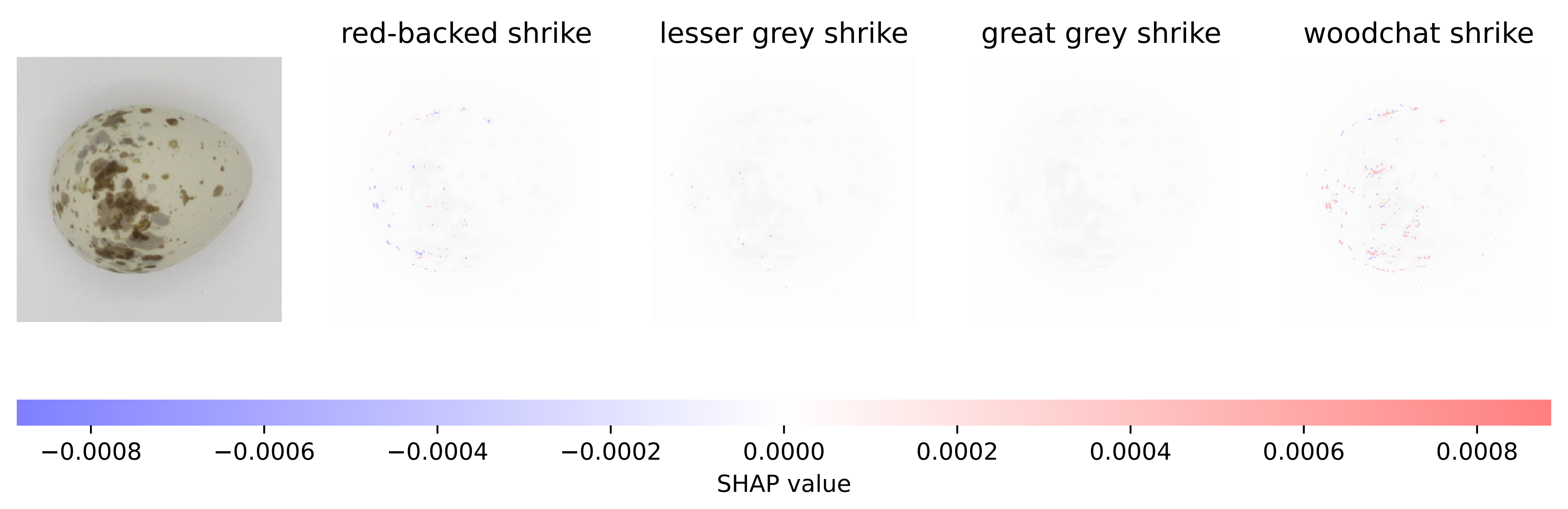

IMG_0331.JPG SHAP DeepExplainer major impact (Q3)

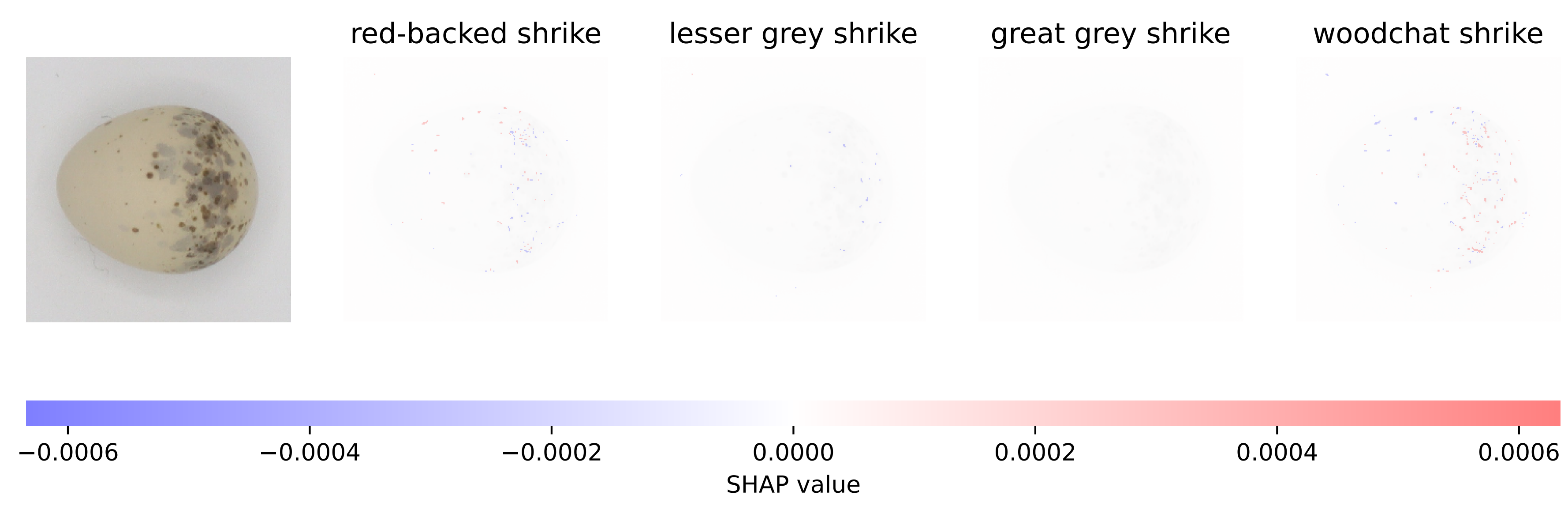

IMG_0482.JPG SHAP DeepExplainer major impact (Q3)

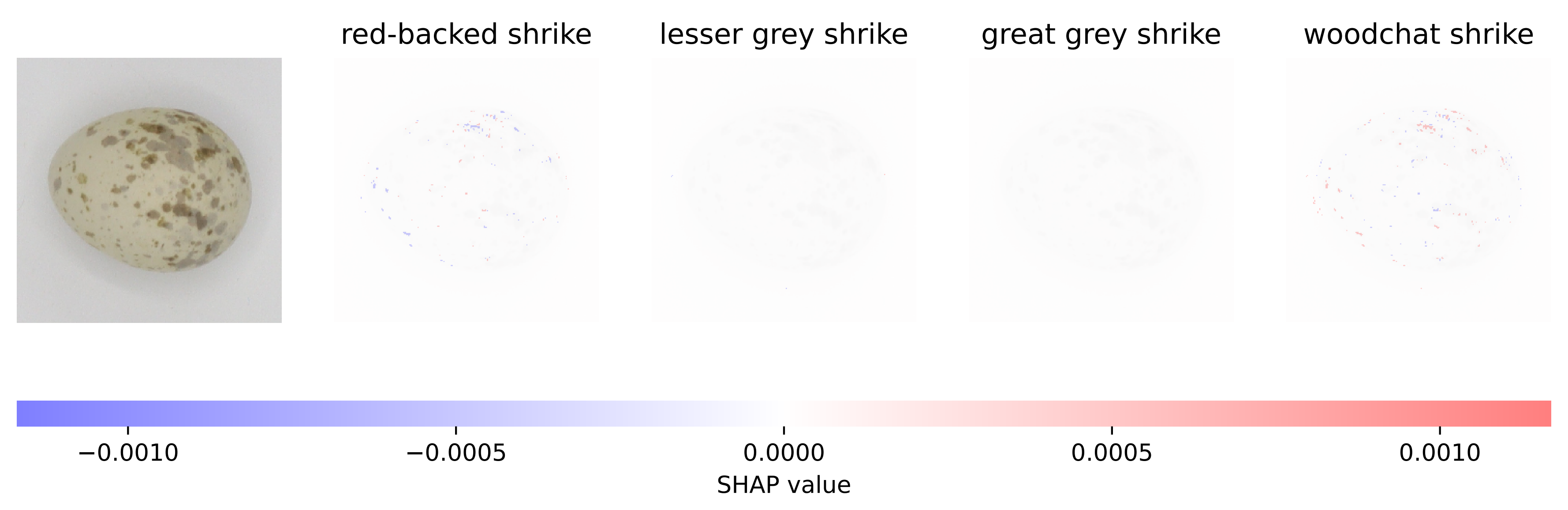

IMG_0567.JPG SHAP DeepExplainer major impact (Q3)

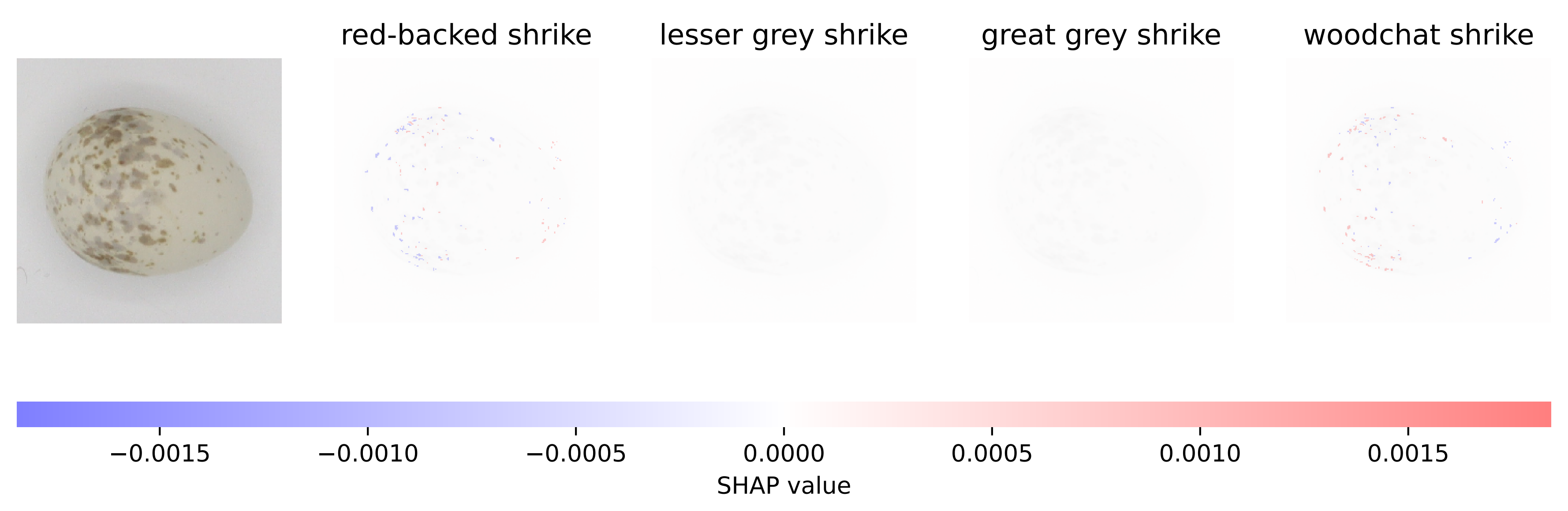

IMG_0610.JPG SHAP DeepExplainer major impact (Q3)

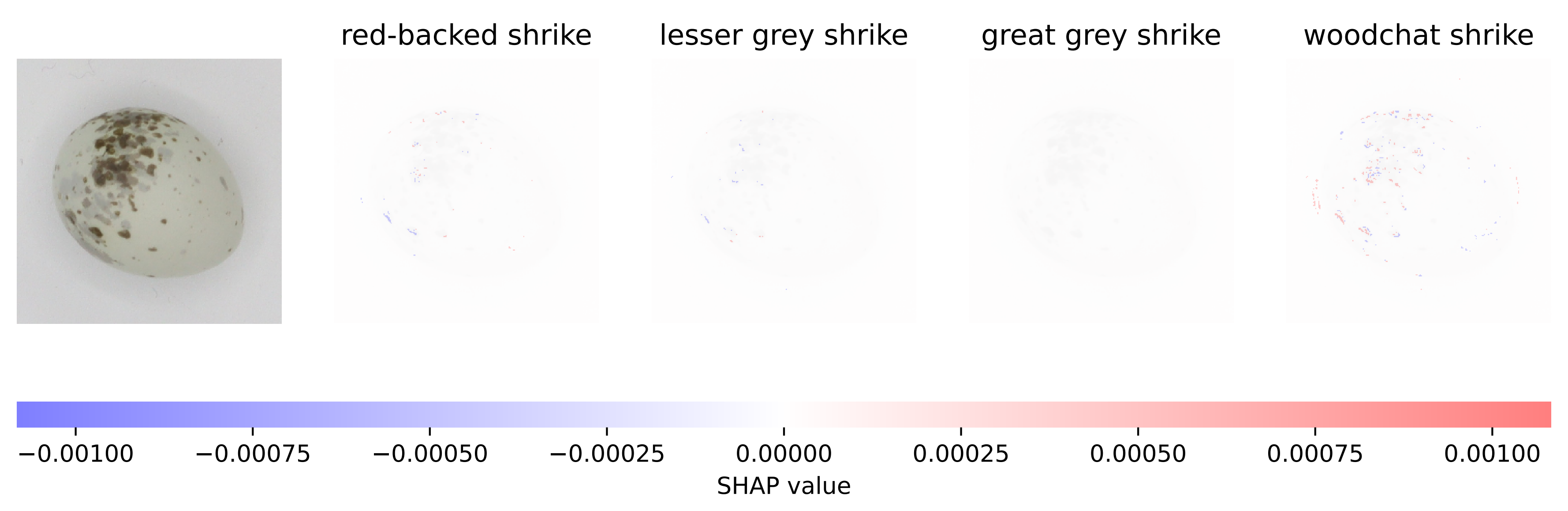

IMG_0705.JPG SHAP DeepExplainer major impact (Q3)

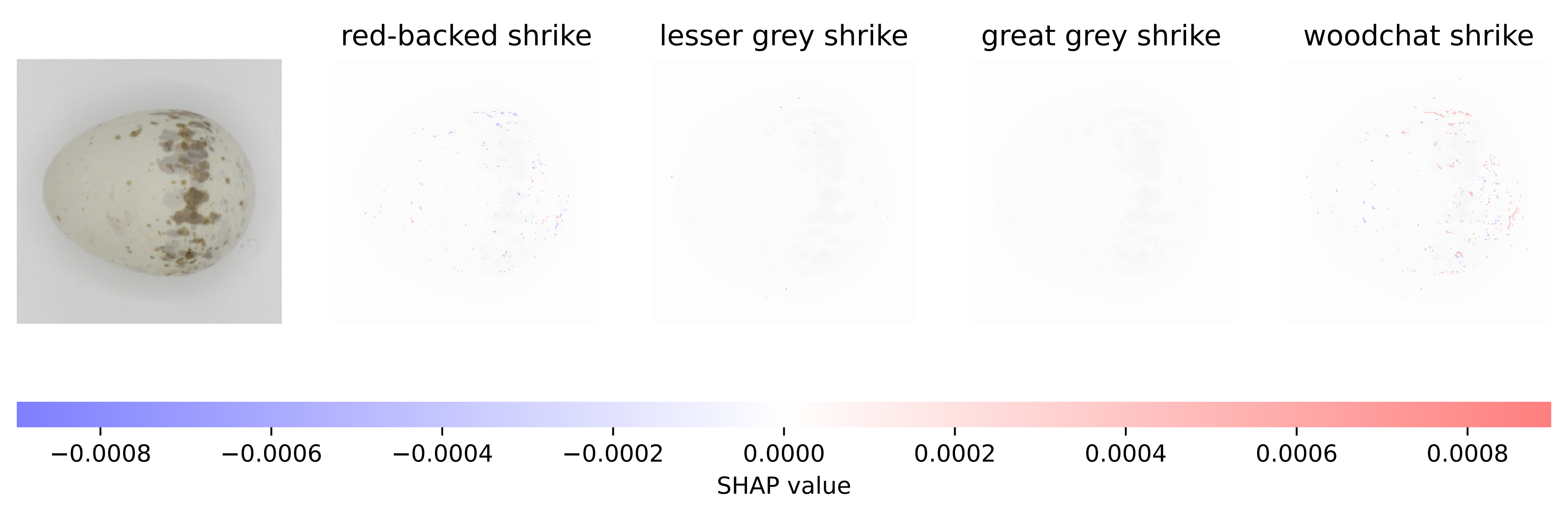

IMG_0720.JPG SHAP DeepExplainer major impact (Q3)

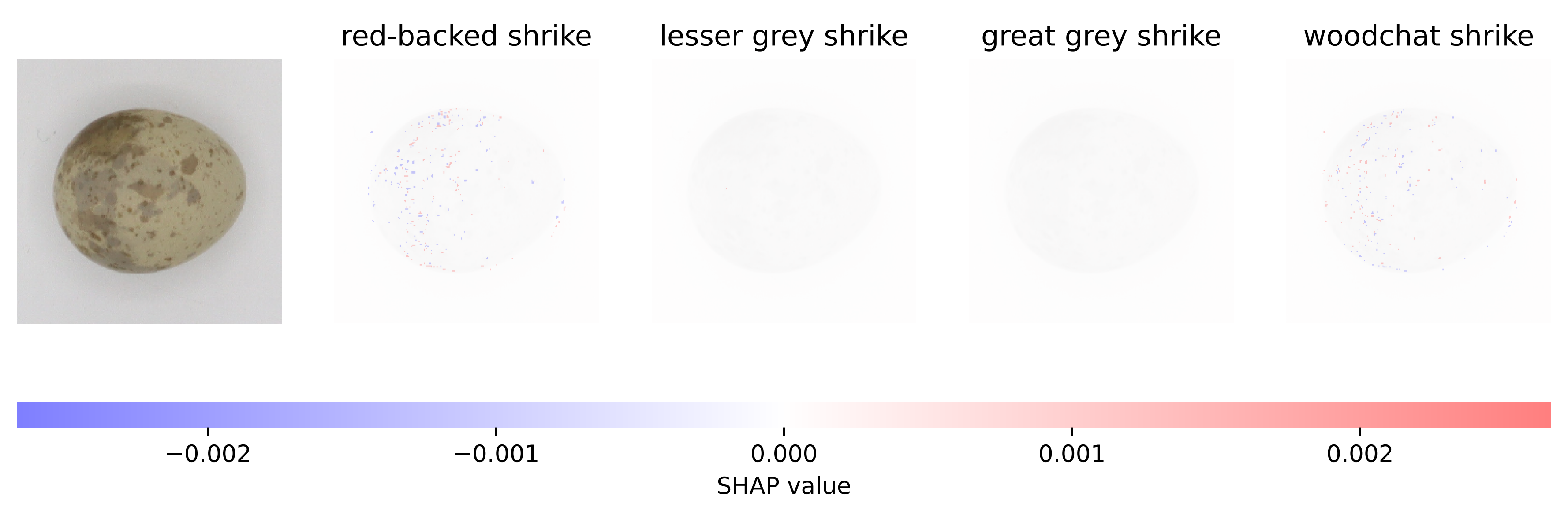

IMG_0780.JPG SHAP DeepExplainer major impact (Q3)

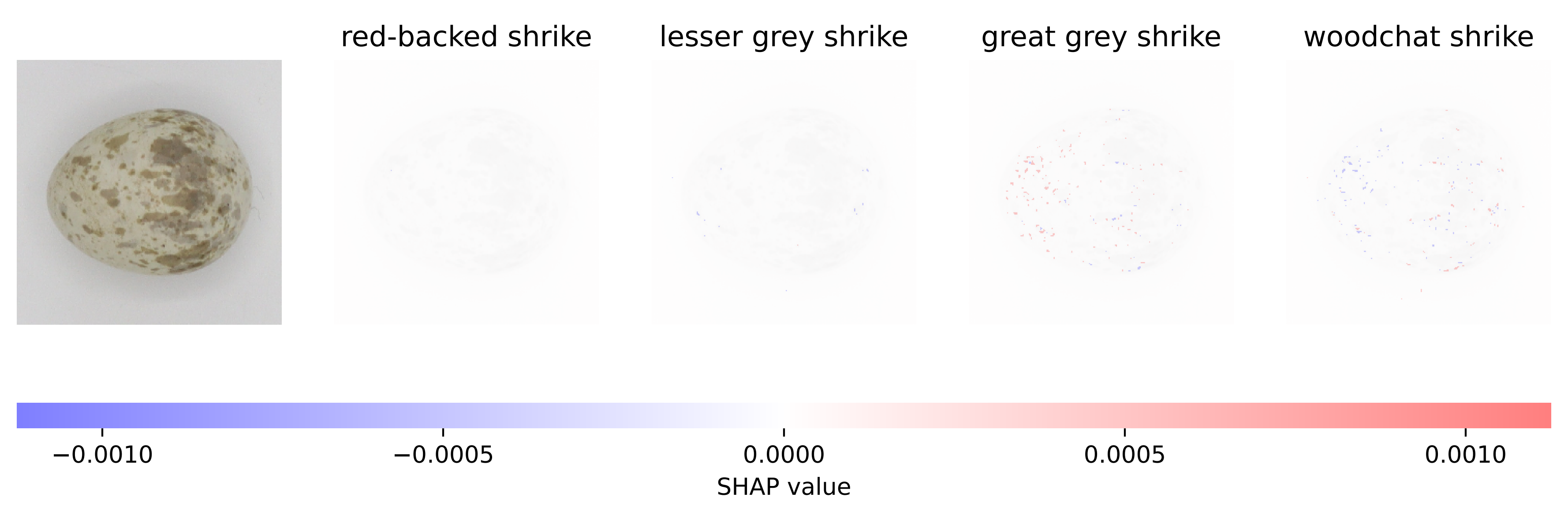

IMG_0849.JPG SHAP DeepExplainer major impact (Q3)

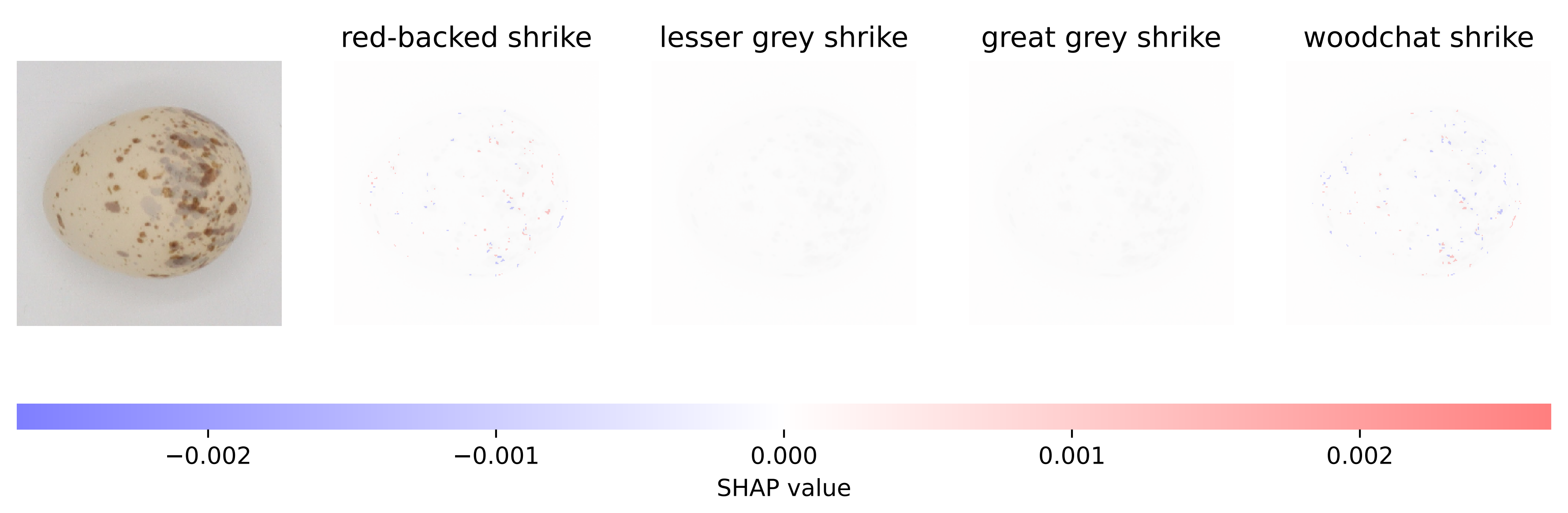

IMG_0850.JPG SHAP DeepExplainer major impact (Q3)

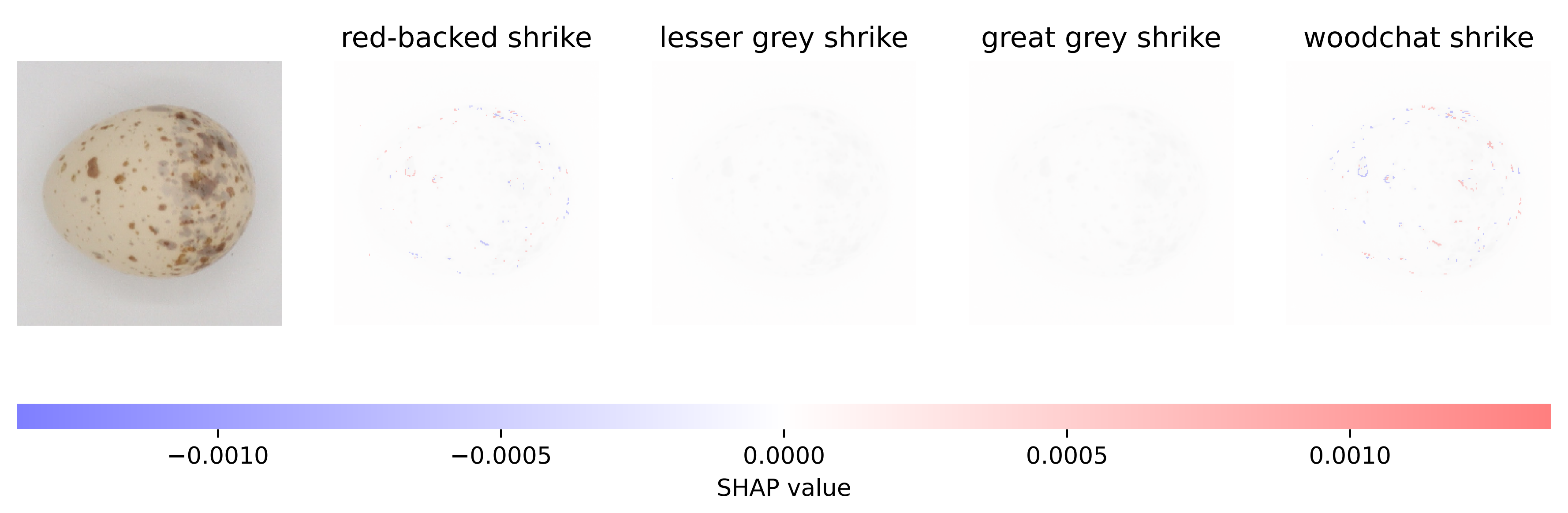

IMG_0988.JPG SHAP DeepExplainer major impact (Q3)

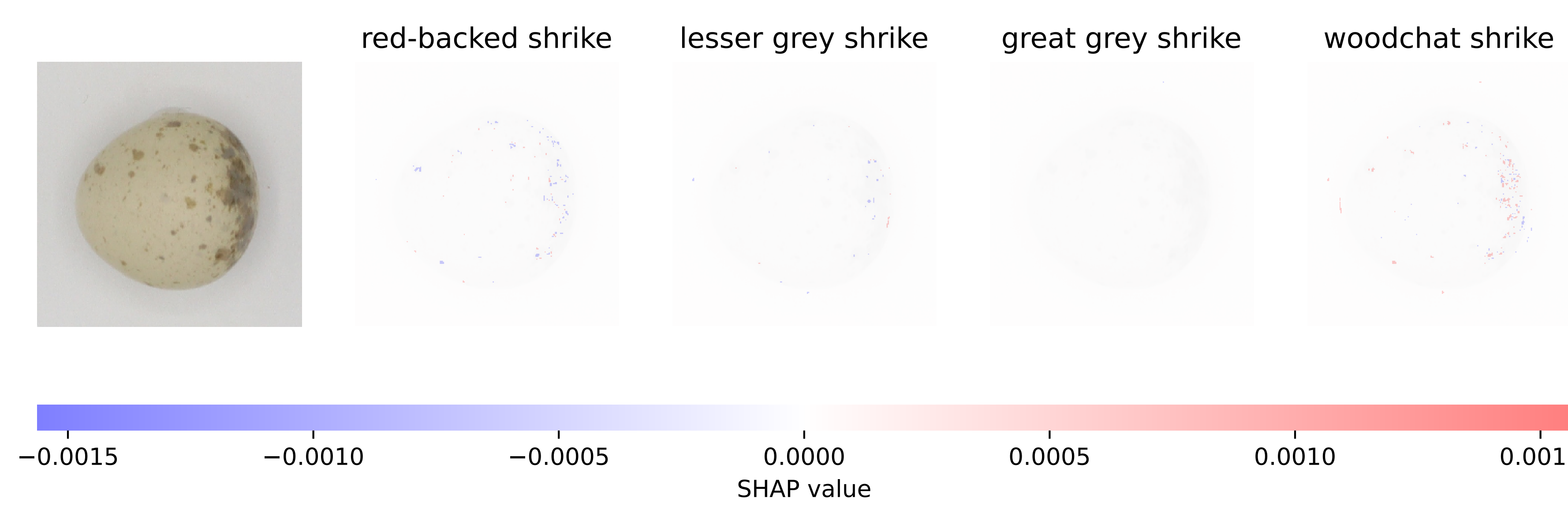

Supplement: S1 File — (ZIP) [file pone.0321532.s001.zip › S1-File-Class-predictions/shap - woodchat shrike - mj.pdf]
