# Supplementary material for: Can explainable AI classify shrike (Laniidae) eggs by uncovering species-wide pigmentation patterns?
Source: PLoS One. 2025 May 2;20(5):e0321532. doi: 10.1371/journal.pone.0321532 (PMC12047758; doi:10.1371/journal.pone.0321532)

IMG_0316.JPG --- True Class: woodchat shrike - Predicted: woodchat shrike - p: 0.9990398

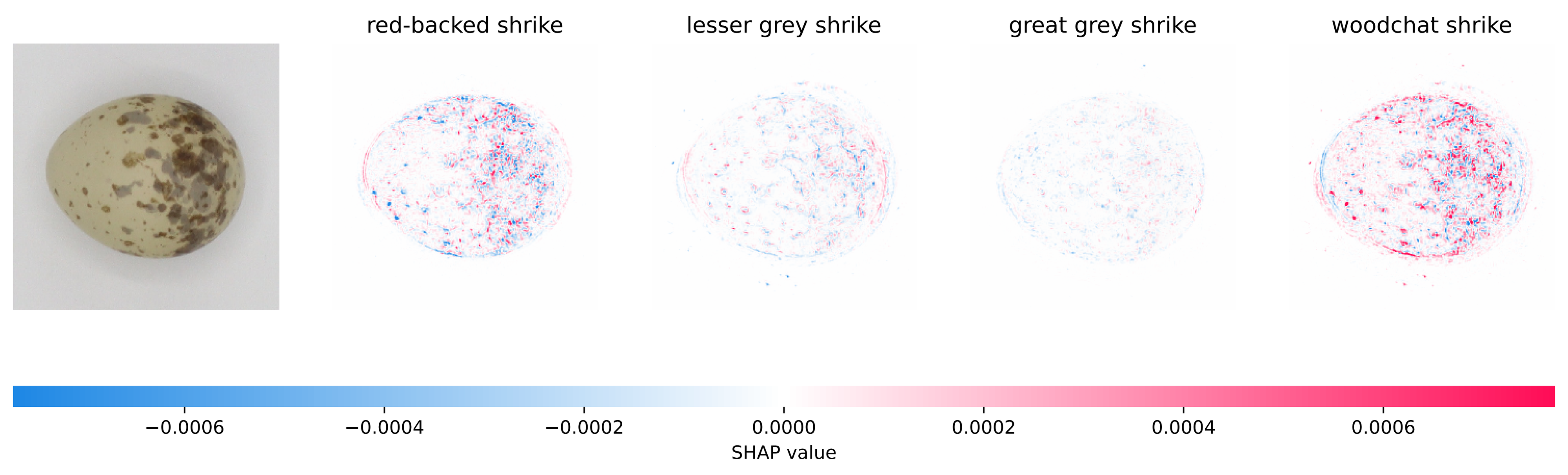

IMG_0320.JPG --- True Class: woodchat shrike - Predicted: woodchat shrike - p: 0.99793684

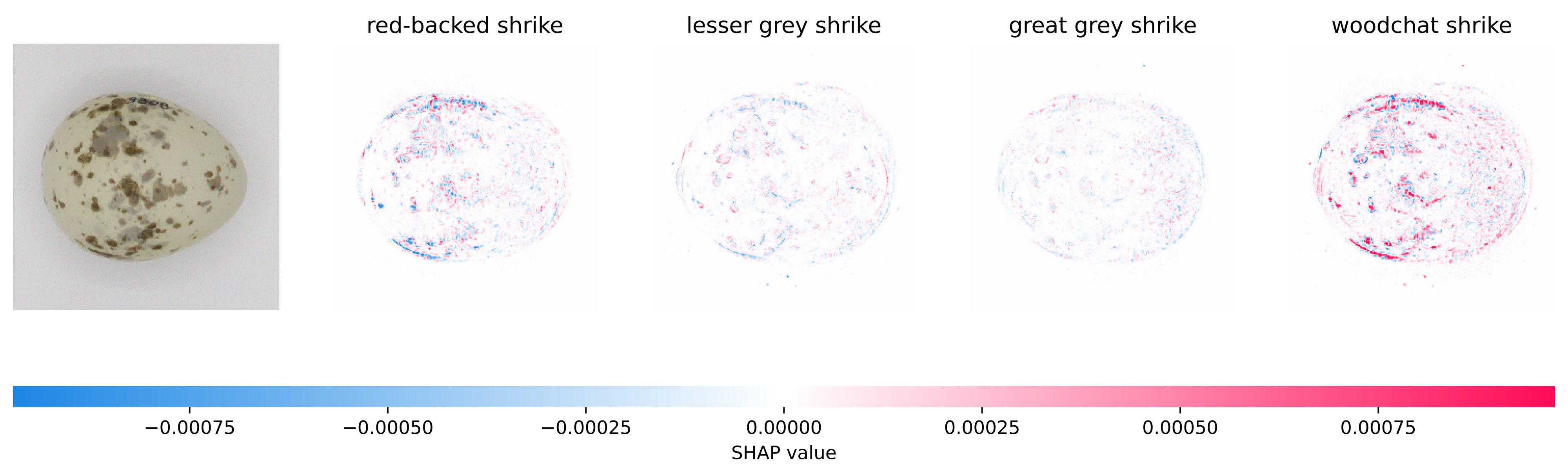

IMG_0324.JPG --- True Class: woodchat shrike - Predicted: woodchat shrike - p: 0.9988034

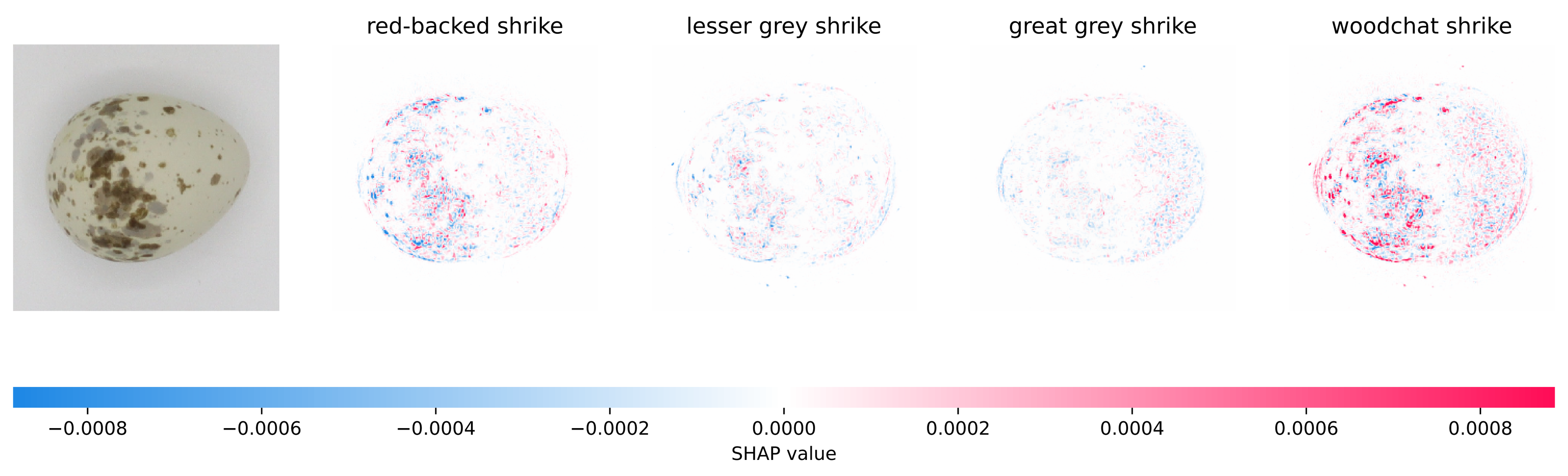

IMG_0331.JPG --- True Class: woodchat shrike - Predicted: woodchat shrike - p: 0.9634352

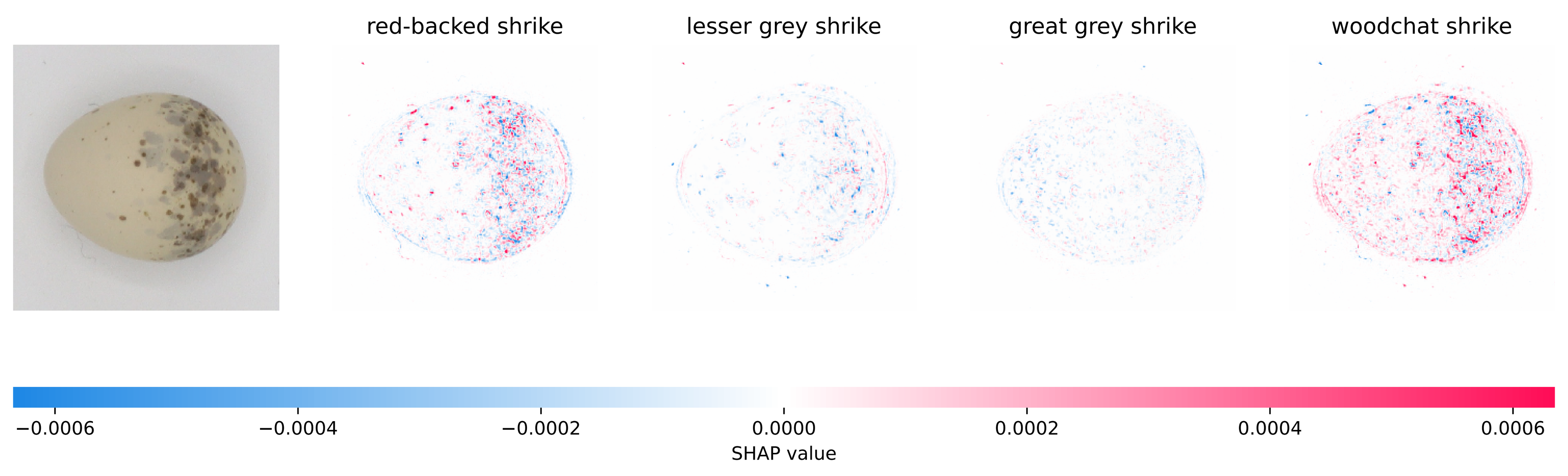

IMG_0482.JPG --- True Class: woodchat shrike - Predicted: woodchat shrike - p: 0.9832358

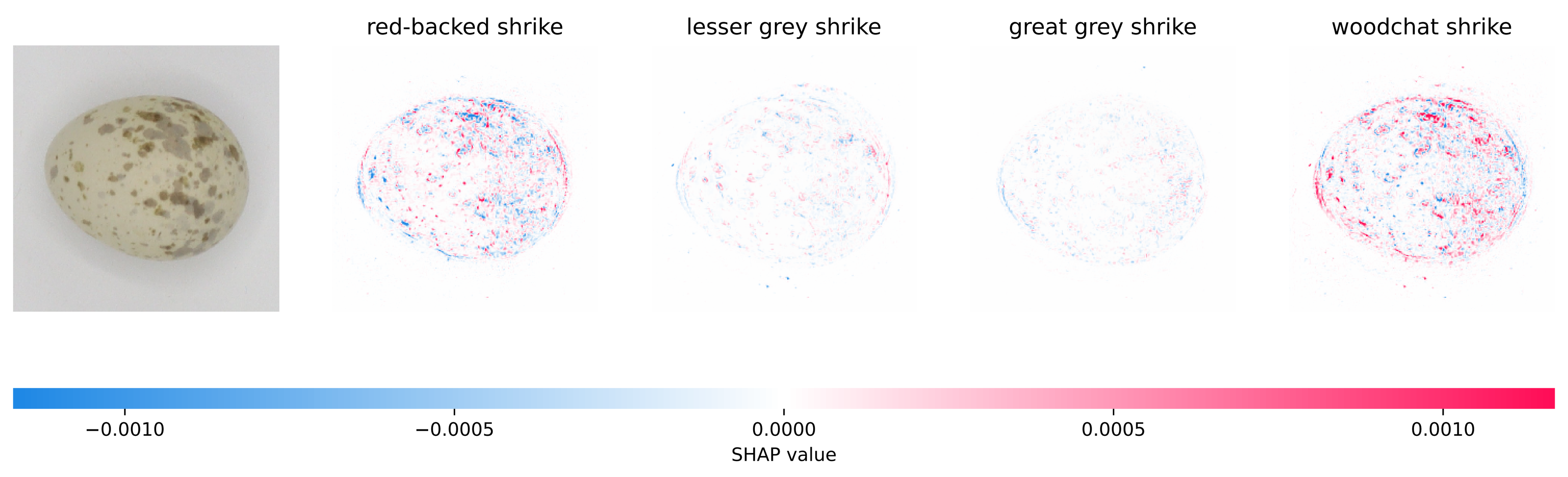

IMG_0567.JPG --- True Class: woodchat shrike - Predicted: woodchat shrike - p: 0.9827053

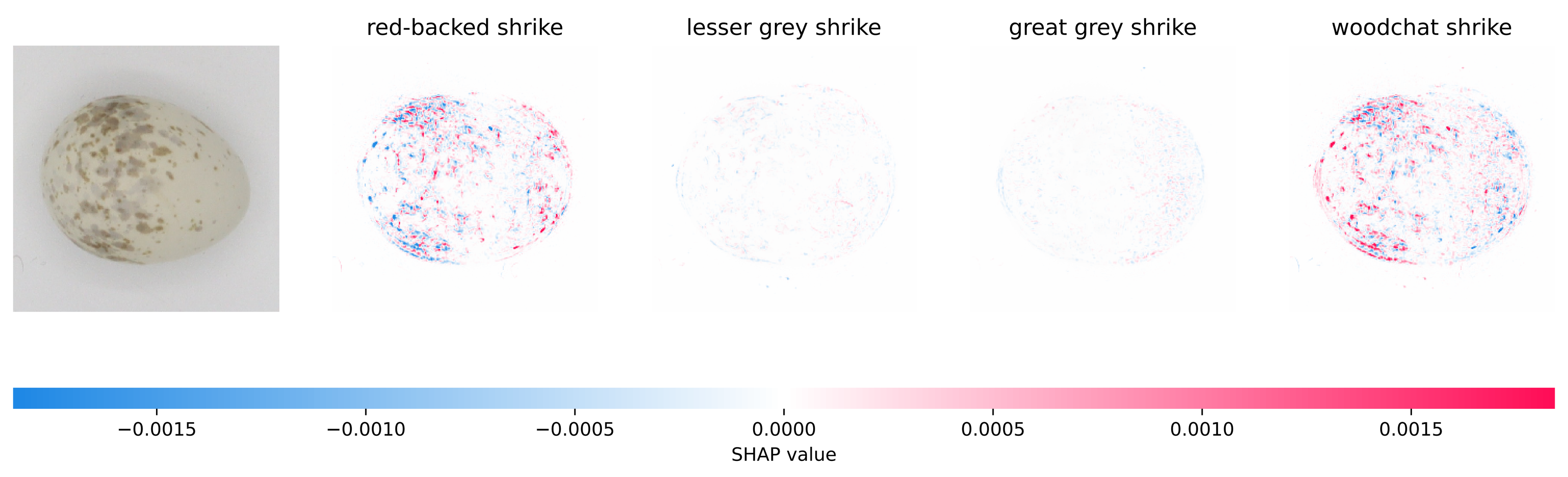

IMG_0610.JPG --- True Class: woodchat shrike - Predicted: woodchat shrike - p: 0.8729479

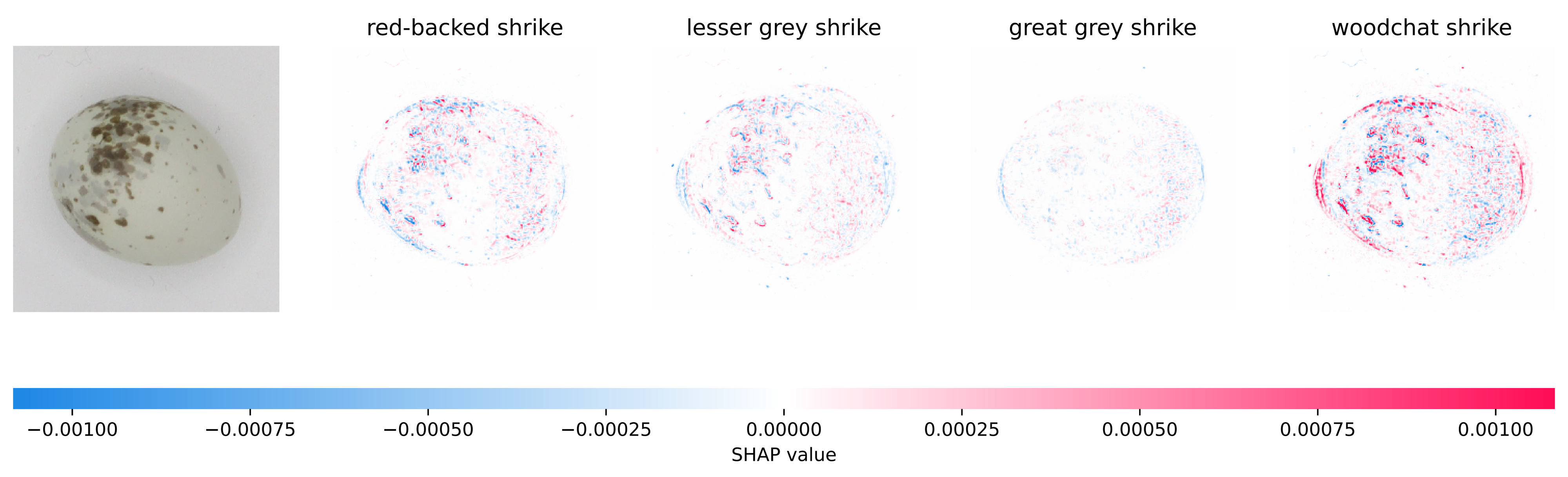

IMG_0705.JPG --- True Class: woodchat shrike - Predicted: woodchat shrike - p: 0.9733357

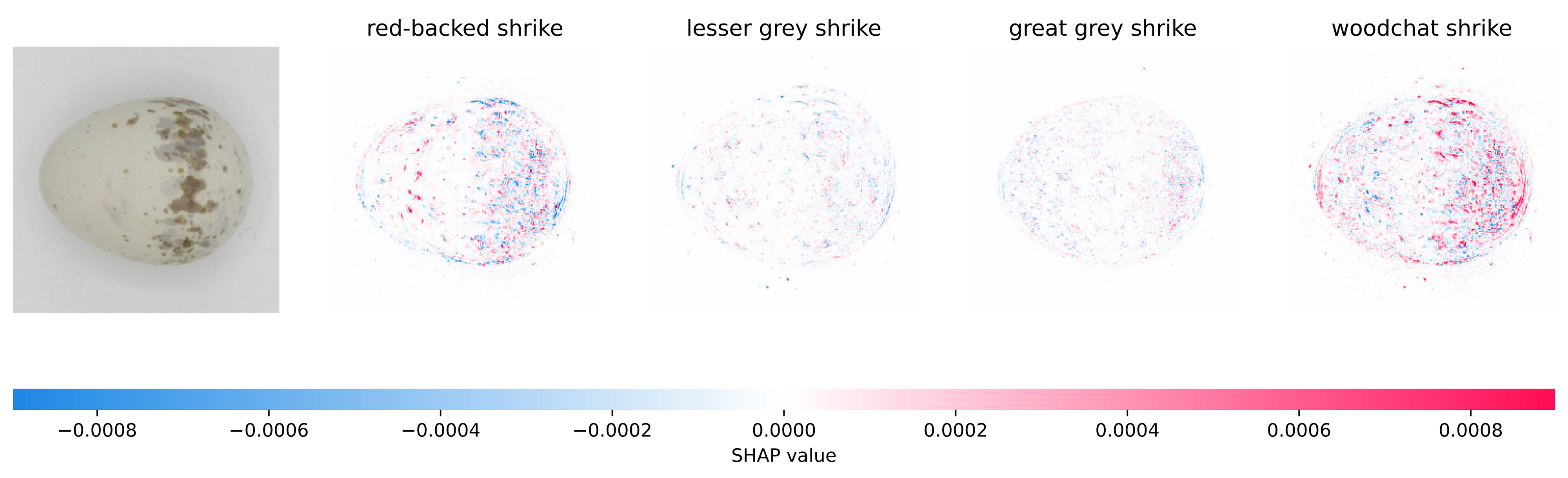

IMG_0720.JPG --- True Class: woodchat shrike - Predicted: woodchat shrike - p: 0.9645799

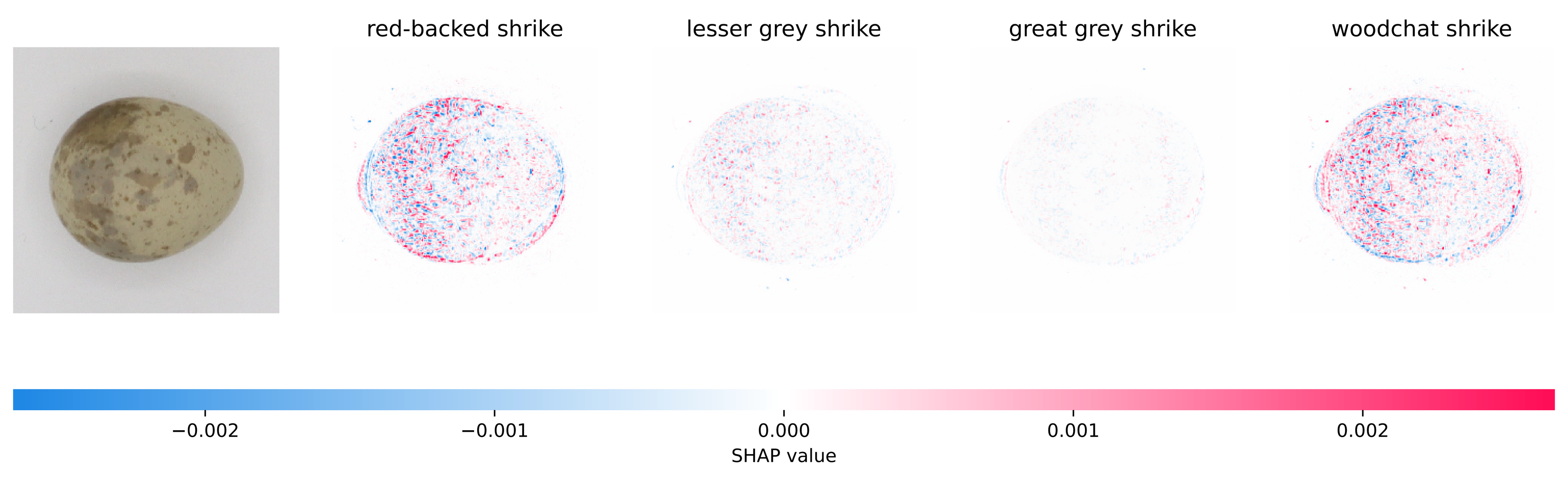

IMG_0780.JPG --- True Class: woodchat shrike - Predicted: great grey shrike - p: 0.6523799

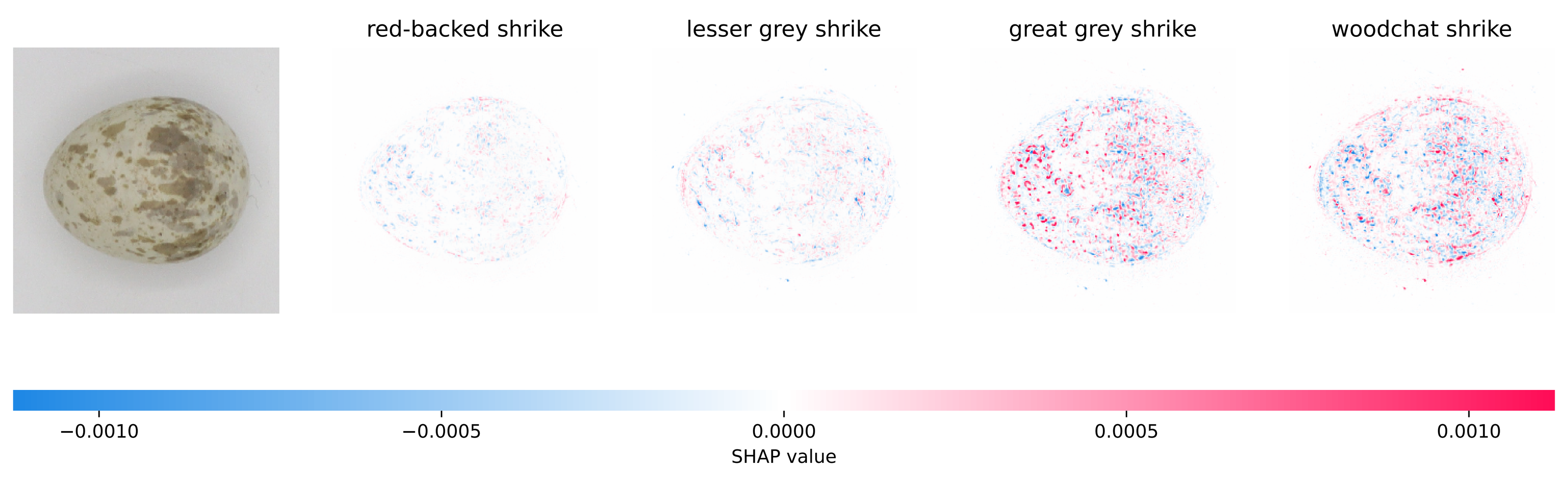

IMG_0849.JPG --- True Class: woodchat shrike - Predicted: red-backed shrike - p: 0.69240195

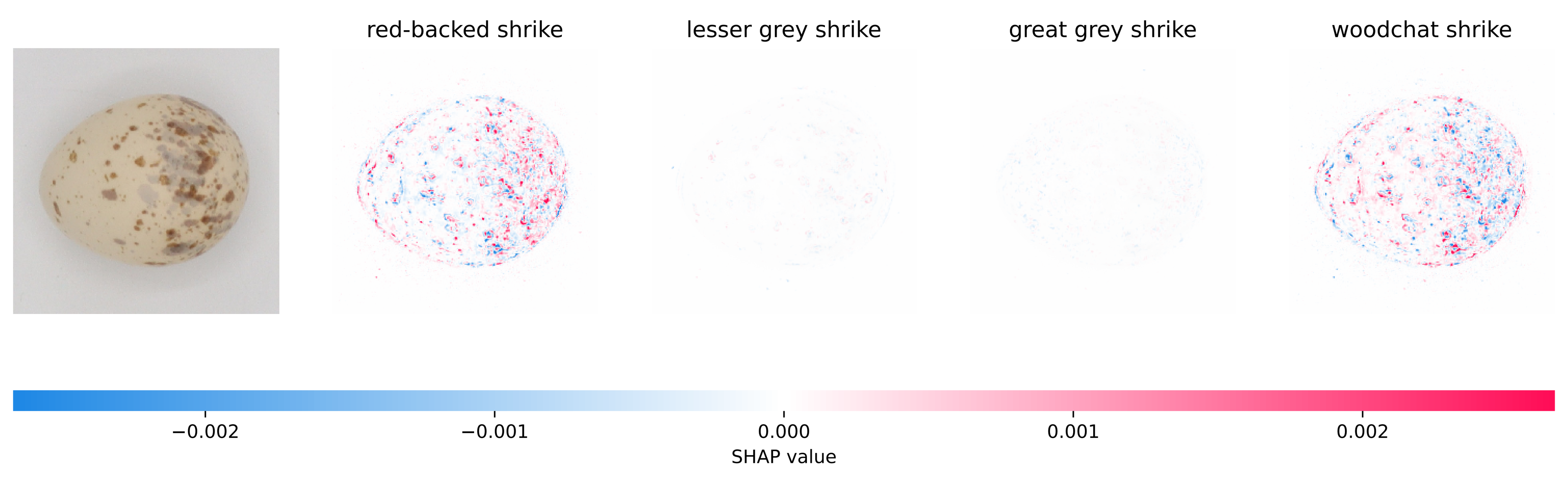

IMG_0850.JPG --- True Class: woodchat shrike - Predicted: woodchat shrike - p: 0.6175761

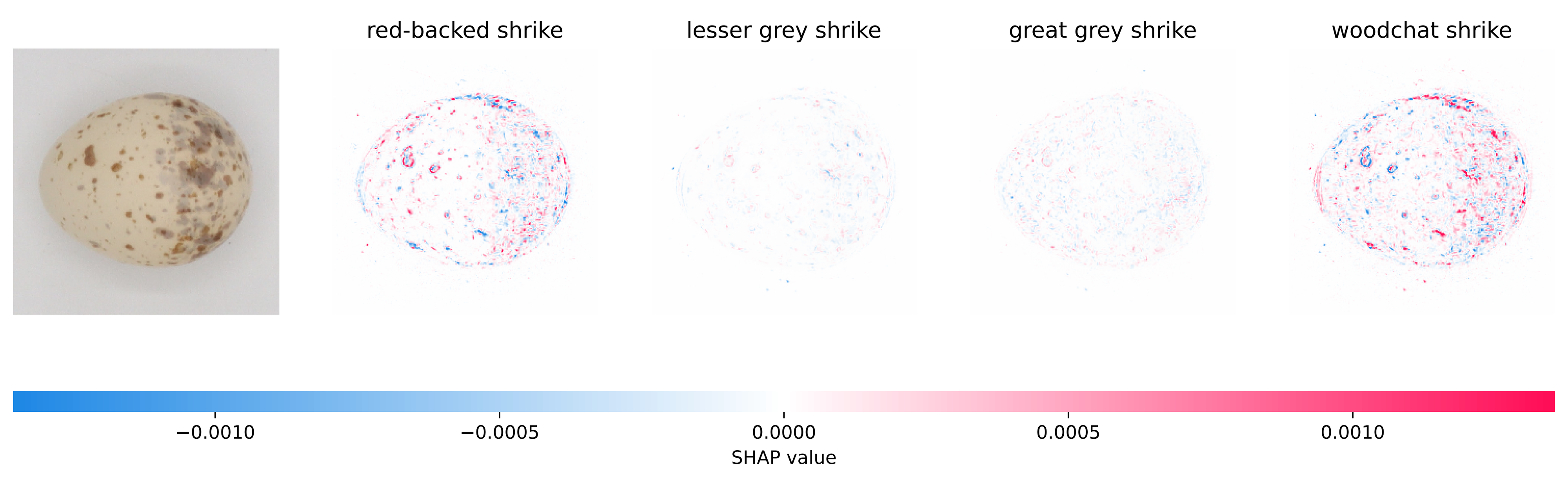

IMG_0988.JPG --- True Class: woodchat shrike - Predicted: woodchat shrike - p: 0.74054664

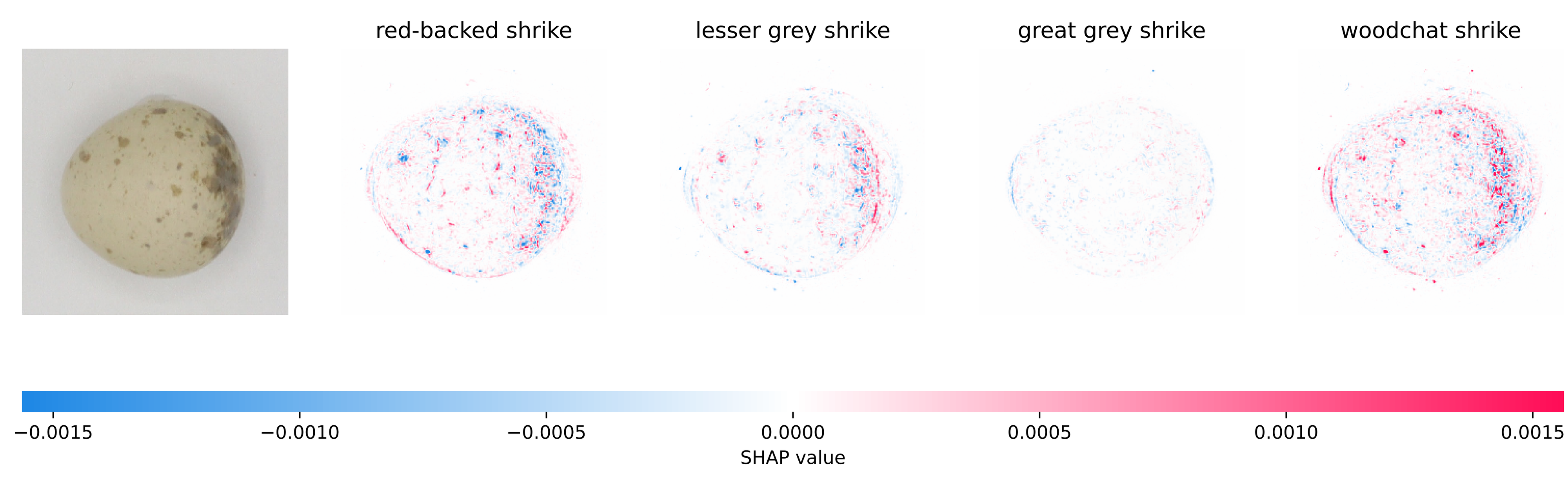

Supplement: S1 File — (ZIP) [file pone.0321532.s001.zip › S1-File-Class-predictions/shap - woodchat shrike.pdf]
