# Supplementary figures and images for: Can explainable AI classify shrike (Laniidae) eggs by uncovering species-wide pigmentation patterns?
Source: PLoS One. 2025 May 2;20(5):e0321532. doi: 10.1371/journal.pone.0321532 (PMC12047758; doi:10.1371/journal.pone.0321532)

IMG\_1519.JPG --- True Class: great grey shrike - Predicted: great grey shrike - p: 0.9917408

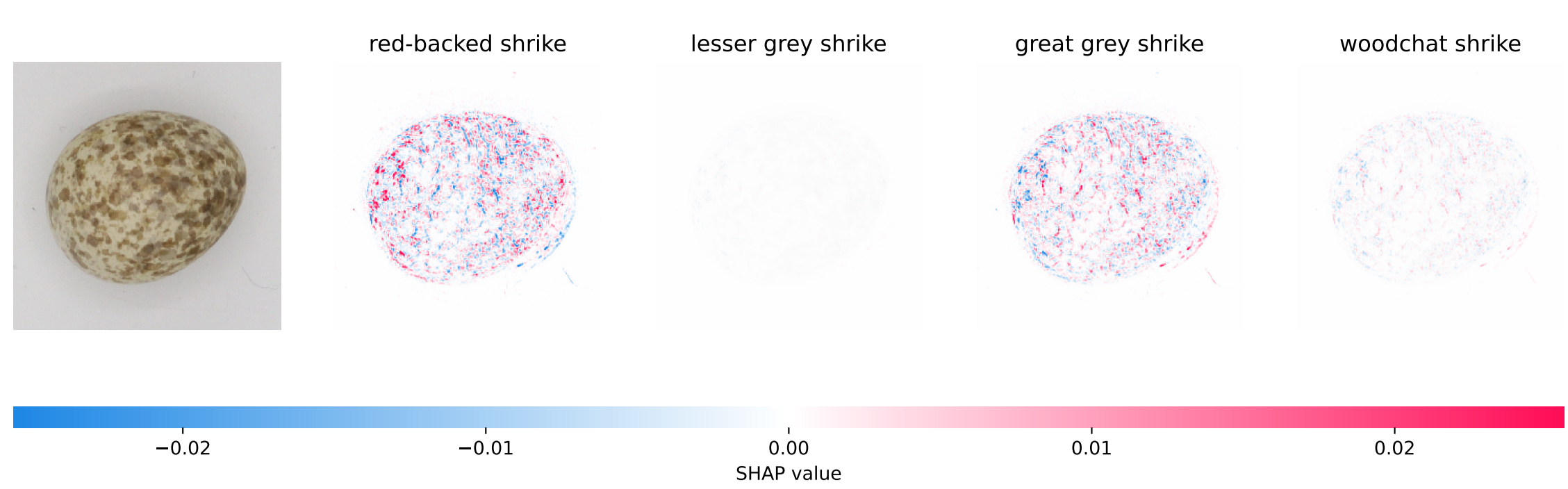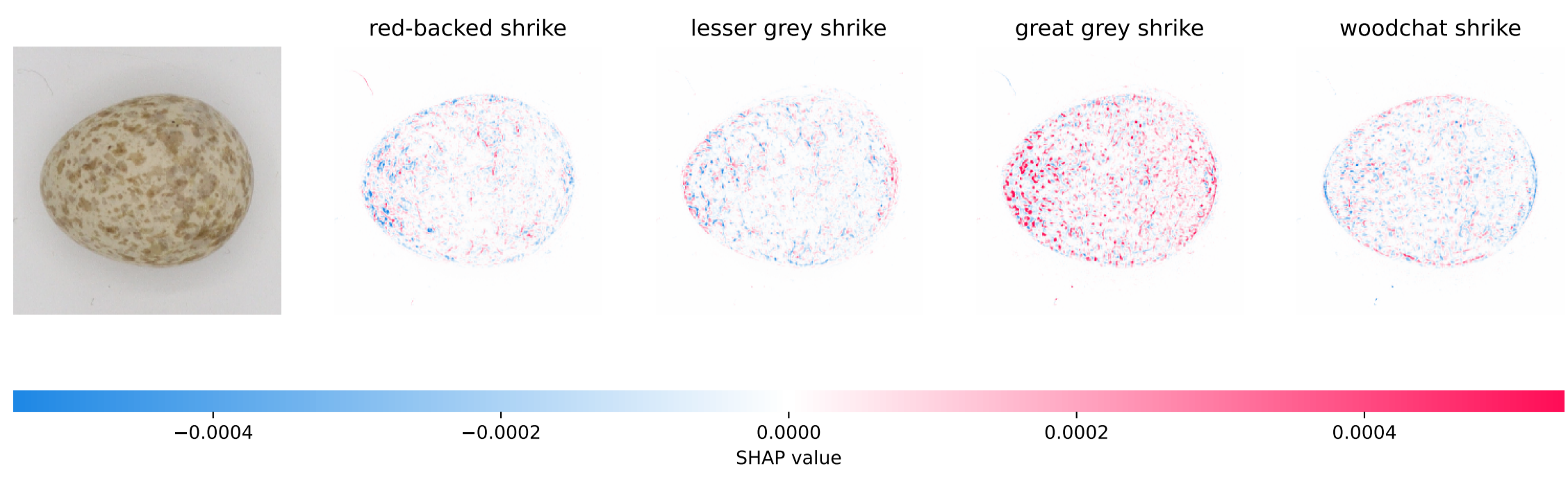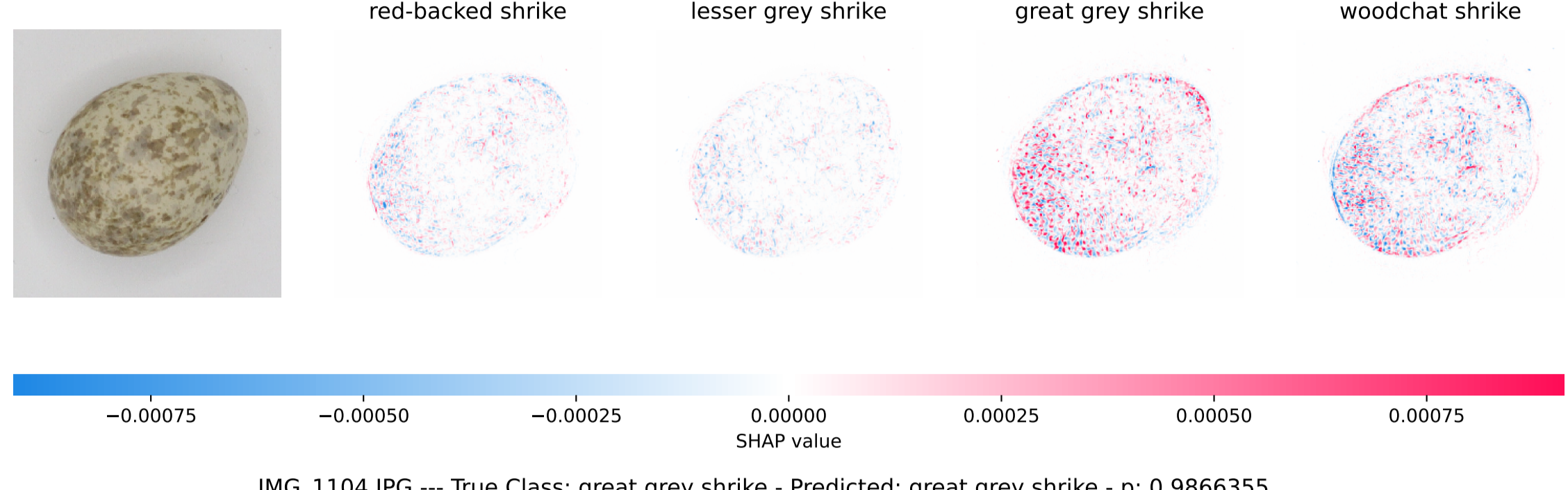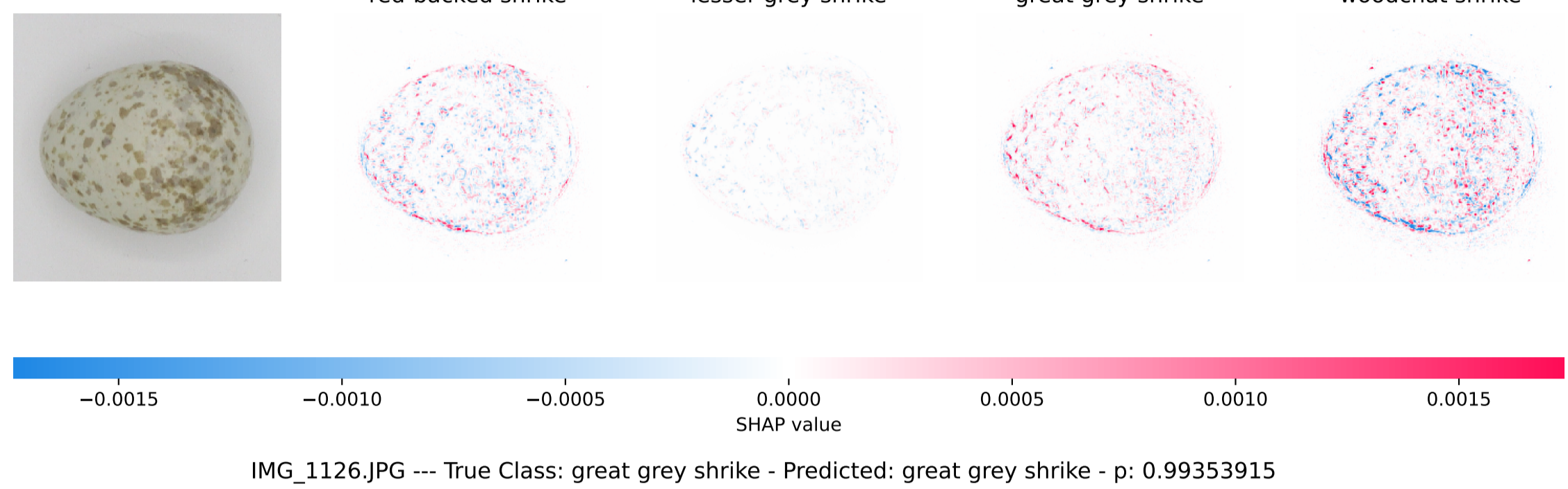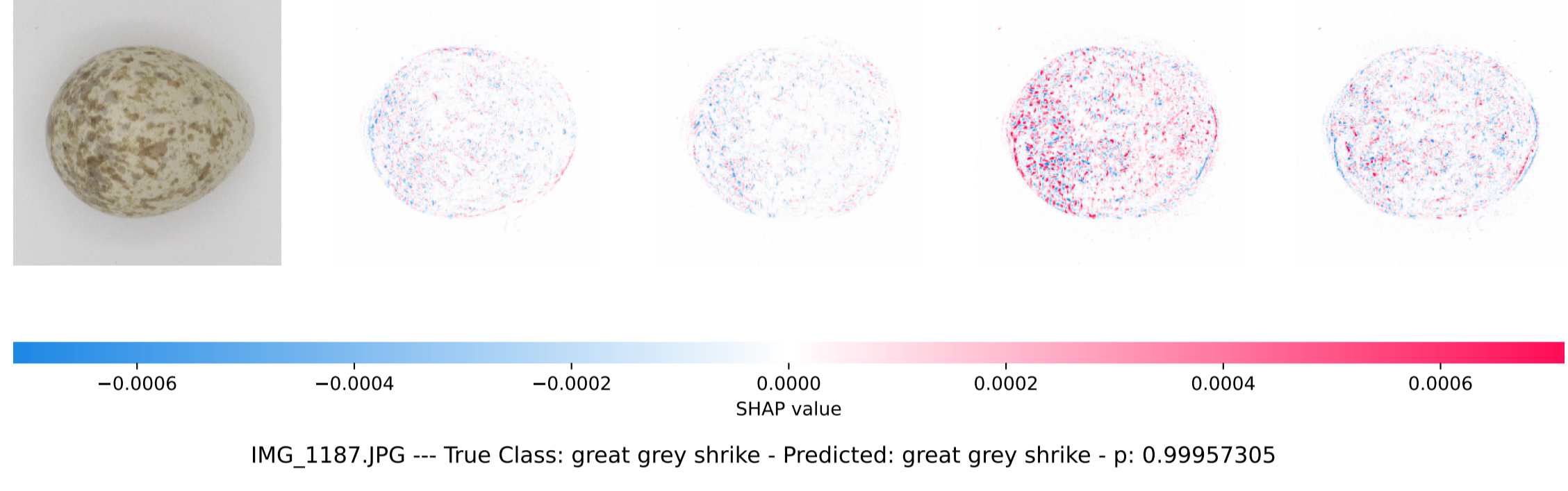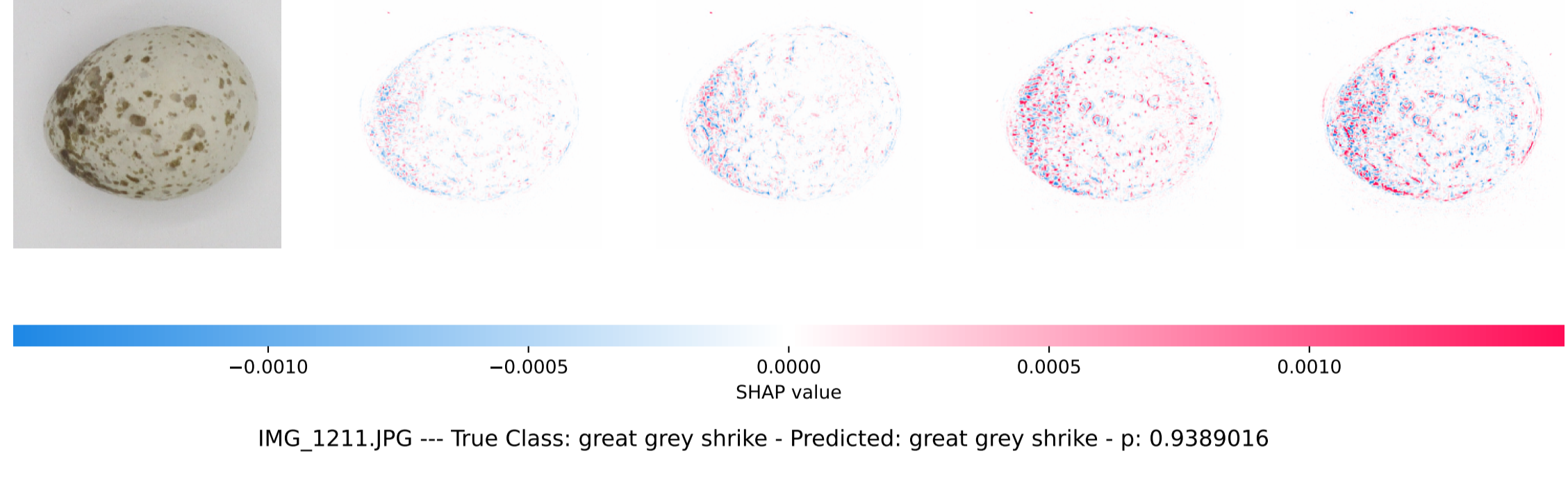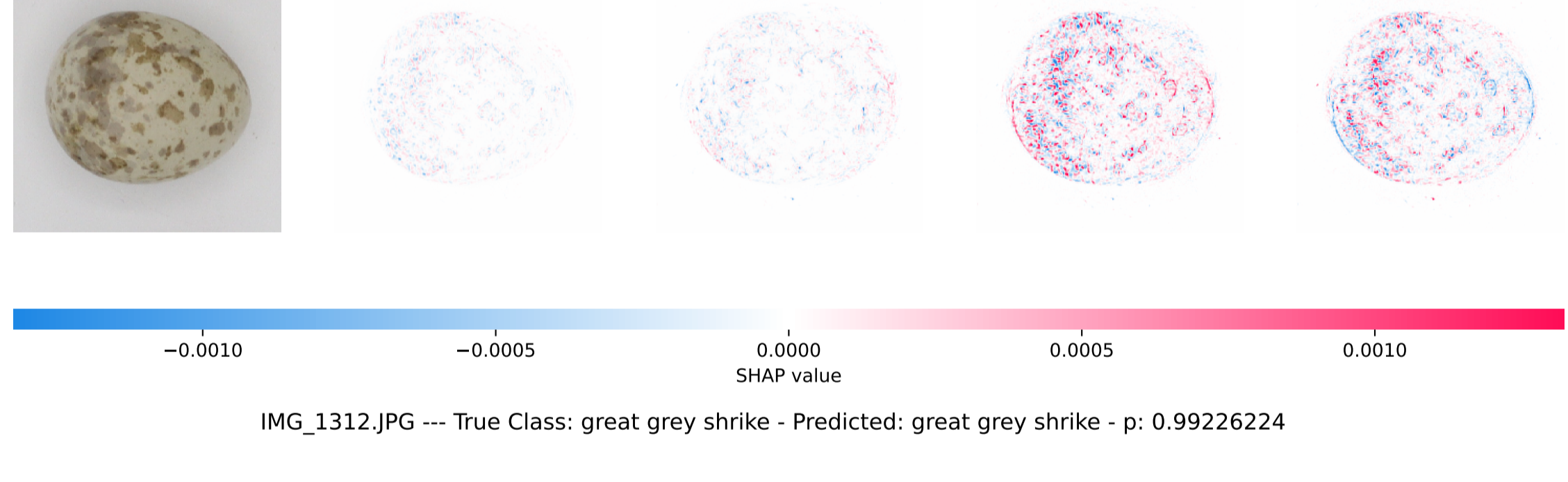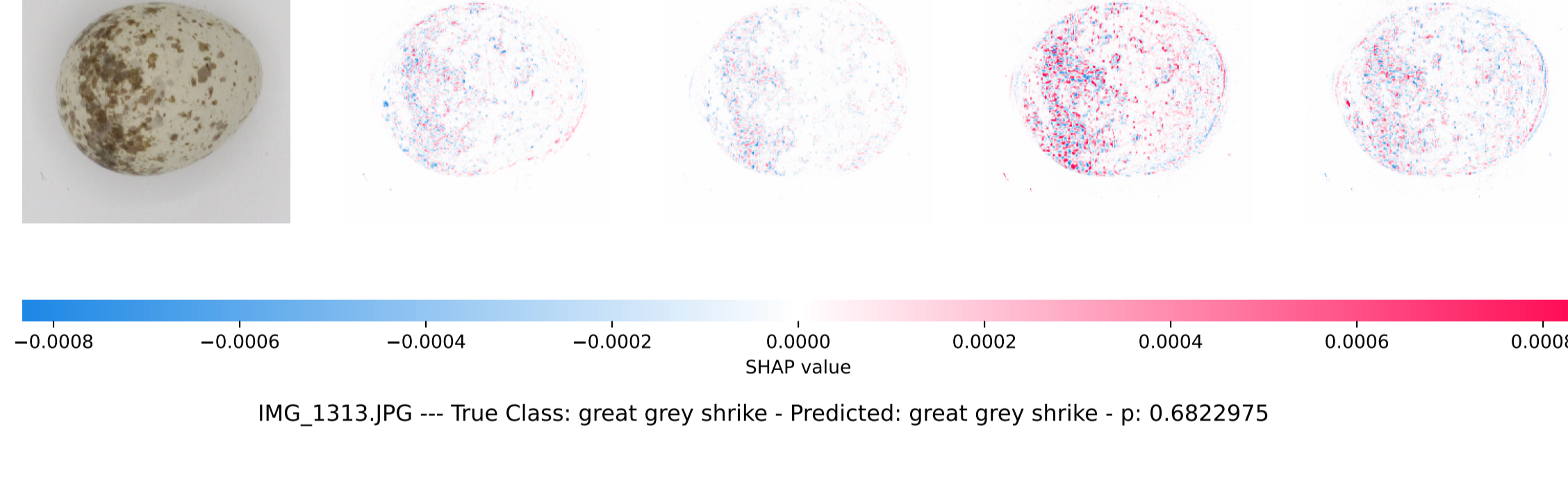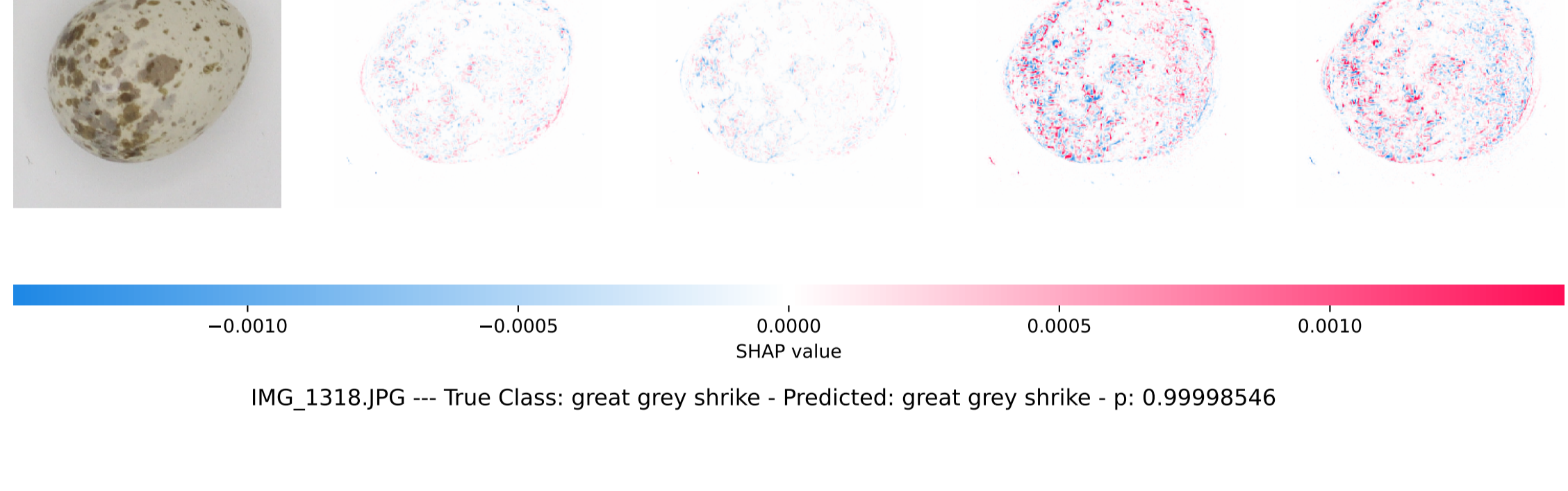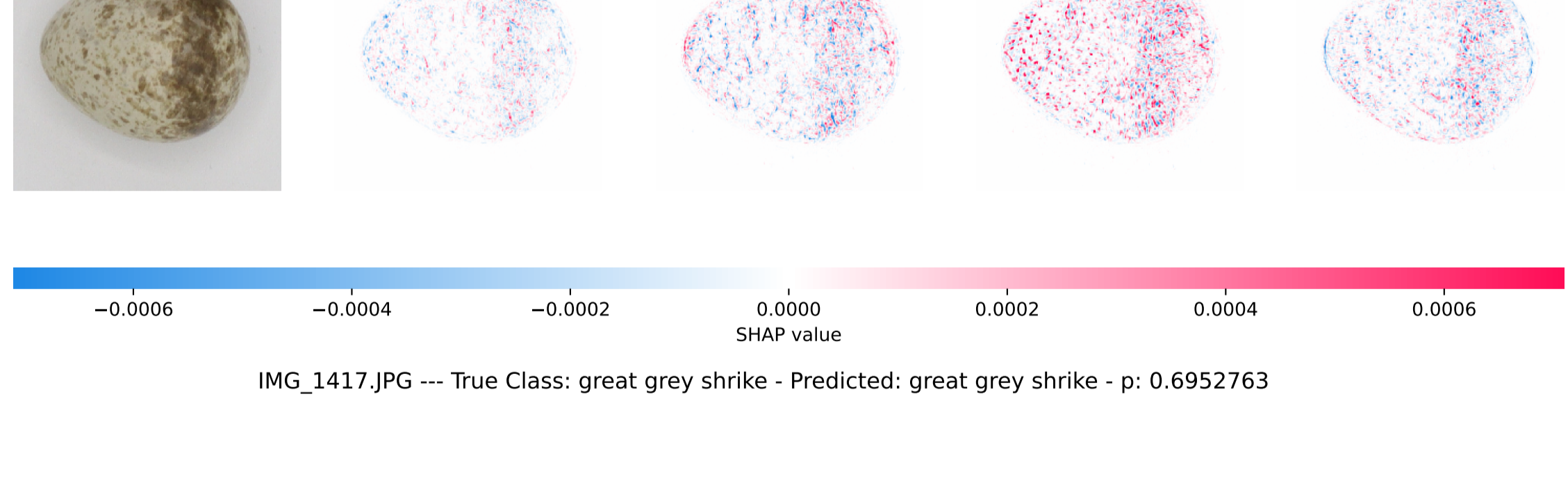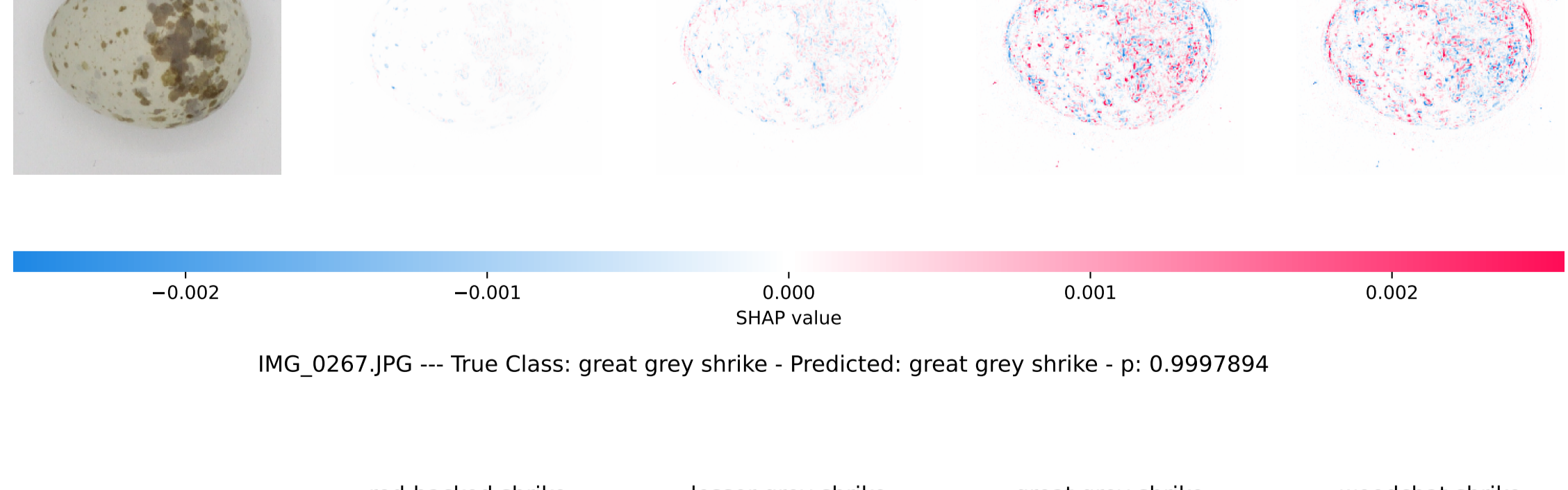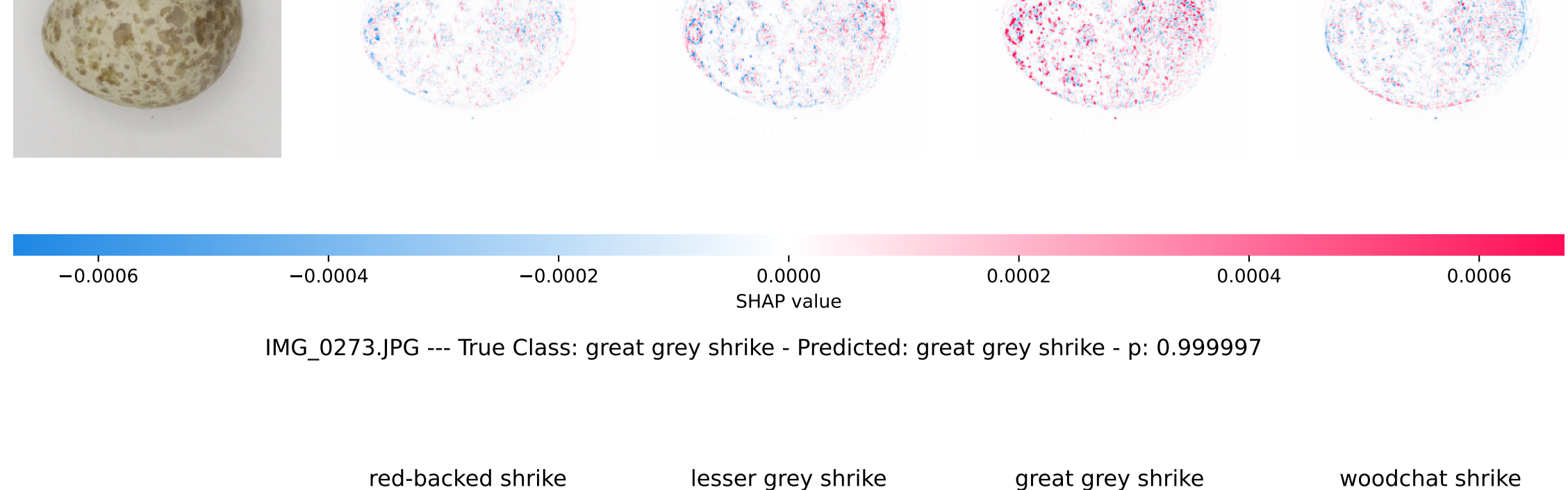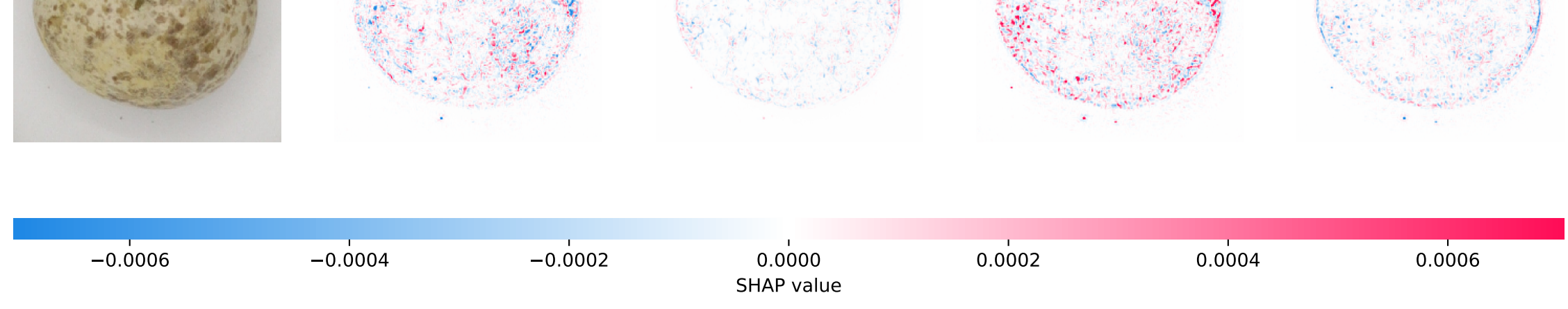

Supplement: S1 File — (ZIP) [file pone.0321532.s001.zip › S1-File-Class-predictions/shap - great grey shrike.pdf]
